# Biological Activities and Chemical Constituents of Essential Oils from *Piper cubeba* Bojer and *Piper nigrum* L.

**DOI:** 10.3390/molecules24101876

**Published:** 2019-05-15

**Authors:** Yusuf Andriana, Tran Dang Xuan, Tran Ngoc Quy, Hoang-Dung Tran, Quang-Tri Le

**Affiliations:** 1 Graduate School for International Development and Cooperation, Hiroshima University, Hiroshima 739-8529, Japan; yusufandriana@yahoo.com (Y.A.); tnquy@ctu.edu.vn (T.N.Q.); 2 Research Center for Appropriate Technology, Indonesian Institute of Sciences, Jl. KS. Tubun No. 5, Subang 41213, Indonesia; 3 Faculty of Biotechnology, Nguyen Tat Thanh University, 298A-300A Nguyen Tat Thanh Street, Ward 13, District 4, Ho Chi Minh City 72820, Vietnam; thdung@ntt.edu.vn; 4 Department of Orthopedic, 7A Military Hospital, 466 Nguyen Trai Street, Ward 8, District 5, Ho Chi Minh City 72706, Vietnam; bstridongnai@yahoo.com.vn

**Keywords:** *Piper cubeba*, *Piper nigrum*, essential oil, antioxidant, antihyperuricemia, *Bidens pilosa*, *Echinochloa crus-galli*

## Abstract

In this study, we evaluated antioxidant, antihyperuricemic, and herbicidal activities of essential oils (EOs) from *Piper cubeba* Bojer and *Piper nigrum* L.; two pepper species widely distributed in tropics, and examined their chemical compositions. Dried berries of *P. cubeba* and *P. nigrum* were hydro-distilled to yield essential oil (EO) of 1.23 and 1.11% dry weight, respectively. In the antioxidant assay, the radical scavenging capacities of *P. cubeba* EO against DPPH and ABTS free radicals were 28.69 and 24.13% greater than *P. nigrum*, respectively. In the antihyperuricemic activity, *P. cubeba* EO also exhibited stronger inhibitory effects on xanthine oxidase (IC_50_ = 54.87 µg/mL) than *P. nigrum* EO (IC_50_ = 77.11 µg/mL). In the herbicidal activity, *P. cubeba* EO showed greater inhibition on germination and growth of *Bidens pilosa* and *Echinochloa crus-galli* than *P. nigrum* EO. Besides, *P. cubeba* EO decreased 15.98–73.00% of photosynthesis pigments of *B. pilosa* and *E. crus-galli*, while electrolyte leakages, lipid peroxidations, prolines, phenolics, and flavonoids contents were increased 10.82–80.82% at 1.93 mg/mL dose. Gas chromatography-mass spectrometry (GC-MS) and liquid chromatography-electrospray ionization-mass spectrometry (LC-ESI-MS) analyses revealed that *P. nigrum* and *P. cubeba* EOs principally possessed complex mixtures of monoterpenes and sesquiterpenes. Terpinen-4-ol (42.41%), α-copaene (20.04%), and γ-elemene (17.68%) were the major components of *P. cubeba* EO, whereas β-caryophyllene (51.12%) and β-thujene (20.58%) were the dominant components of *P. nigrum* EO. Findings of this study suggest both *P. cubeba* and *P. nigrum* EOs were potential to treat antioxidative stress and antihyperuricemic related diseases. In addition, the EOs of the two plants may be useful to control *B. pilosa* and *E. crus-galli*, the two invasive and problematic weeds in agriculture practice.

## 1. Introduction

Essential oils (EOs) of plants have been extensively used in food, cosmetic, healthcare, and agriculture industries [1,2]. EO is the hydrophobic liquid containing volatile aromatic compounds and usually extracted from plants tissues by hydro-distillation [3]. It is considered of great importance due to its biological properties, for example antioxidant, anti-aging, anti-melanogenic, anti-inflammatory, anti-fungal, or anti-aflatoxin capacity [2,4]. In herbicidal activity, essential oils extracted from *Tagetes erecta*, *Satureja hortensis*, *Citrus aurantiifolia*, and *Tetraclinis articulate* have been reported to have phytotoxic property on some weed species [1,5,6,7].

The genus *Piper* (Piperaceae family) consists of more than 700 species distributed in tropical and subtropical regions in the world and is used mainly for spices and seasonings. Traditionally, this genus is employed to treat inflammation, skin irritation, bronchitis, and intestinal pains [8,9]. Some EOs from this genus for example *P. gaudichaudianum*, *P. humaytanum*, *P. permucronatum*, and *P. hostmanianum* exhibit larvicidal activity [10]. Another species, such as *P. angustifolium* EO possesses antileishmanial capacity [11]. In comparison to other species from the genus of *Piper*, biological activities of *Piper cubeba* and *Piper nigrum* EOs have received less attention so far and have not yet been well documented.

*P. cubeba*, or tailed pepper, a plant originated from Java and Borneo, sometimes called as Java pepper, is mostly cultivated for its berries and essential oil [12]. Normally, this plant is utilized as a traditional medicine to treat gonorrhea, dysentery, syphilis, abdominal pain, diarrhea, enteritis, and asthma diseases [13]. Some biological activities of *P. cubeba* EO for instance antiparasitic [14], antimicrobial [15], insecticidal activities [16] have been reported. Sabinene, β-elemene, and cubebol are the principal components of *P. cubeba* EO extracted from its berries [17].

*P. nigrum*, or black pepper, is native in Kerala in Southwestern India and widely distributed in tropical regions, including Indonesia. Its fruits are usually dried and used for spices and seasonings. This plant is used as a folk medicine to treat gastrointestinal disorder, rheumatic, flu, colds, muscular aches, and fever diseases [12]. Biological activities of *P. nigrum* EO have been known as insecticidal [18,19], larvicidal [20], antioxidant [12], and antimicrobial activities [21]. Caryophyllene and limonene have been reported as the dominant compounds of *P. nigrum* EO [22]. For antihyperuricemia, ethanol, water, and methanol extracts of *P. nigrum*, and piperine, have been known to be active substances against xanthine oxidase [23,24,25]. However, the inhibitory effect of essential oil of this plant on xanthine oxidase to the best of our knowledge is still unknown.

The present study evaluated antioxidant, antihyperuricemic, and herbicidal activities of *P. cubeba* and *P. nigrum* EOs, and examined their chemical compositions. Xanthine oxidase, the key enzyme played vital roles to cause hyperuricemia and gout, was employed to evaluate inhibitory capacity of these EOs. Two noxious weeds, *B. pilosa* and *E. crus-galli*, were selected as indicator plants in herbicidal assays. Physiological and biochemical responses of the two indicator plants to *P. cubeba* and *P. nigrum* EOs were also evaluated.

## 2. Results

### 2.1. Antioxidant and Antihyperuricemic Activities

Antioxidant and antihyperuricemic activities of *P. cubeba* and *P. nigrum* EOs were presented in Table 1. *P. cubeba* EO showed higher inhibitory effects on both antioxidant and antihyperuricemic activities. The IC_50_ values of *P. cubeba* EO in DPPH and ABTS assays were 0.28 and 0.24-folds stronger than *P. nigrum*. In line with antioxidant capacity, *P. cubeba* EO also exhibited more inhibitory effect on xanthine oxidase than *P. nigrum*. However, compared with BHT and allopurinol as positive controls of antioxidant and XOI activities, both of EOs presented lower inhibitory effects.

### 2.2. Herbicidal Activity of P. cubeba and P. nigrum EOs against B. pilosa and E. crus-galli

The inhibitory effects (IC_50_) of *P. cubeba* and *P. nigrum* EOs on germination and growth of two noxious weeds, *B. pilosa* and *E. crus-galli*, were indicated in Table 2. The values of IC_50_ of the two EOs on germination and growth of indicator plants were varied. Inhibitory effects of *P. cubeba* EO on the roots and shoots elongation of *B. pilosa* were 4.16 and 37.74% stronger than *P. nigrum* respectively. Similarly, *P. cubeba* EO gave more 18.72% inhibition effects on the shoot growth of *E. cruss-gali* than *P. nigrum* EO. It appears that *P. cubeba* EO may contain more growth inhibitors than *P. nigrum* EO.

### 2.3. Physiological and Biochemical Responses of B. pilosa and E. crus-galli to P. cubeba and P. nigrum EOs

#### 2.3.1. Pigments Contents

**Figure 1:**
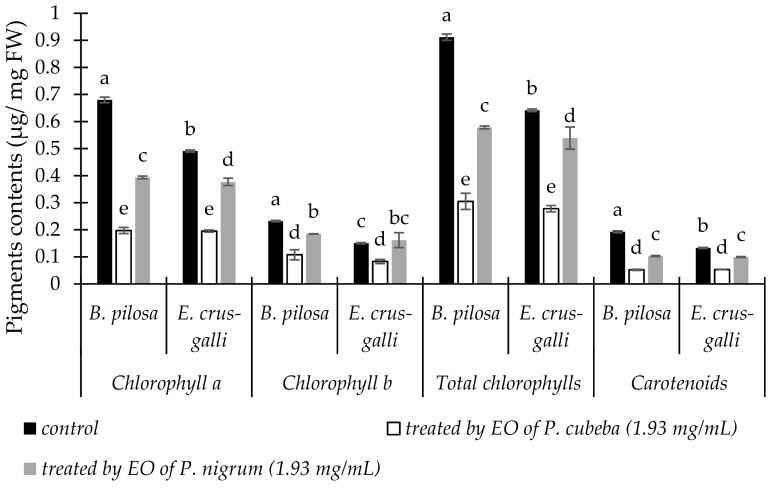
The changes of pigment contents of *B. pilosa* and *E. crus-galli* between control and treatments. Data were presented in means ± standard deviations (SD). Means with different small letter in the same pigment indicted significantly different by Fisher’s test (*p* < 0.05).

The responses of *B. pilosa* and *E. crus-galli* in chlorophylls and carotenoids contents to *P. cubeba* and *P. nigrum* EOs were presented in Figure 1. Both of EOs decreased chlorophyll a, b, total chlorophylls, and carotenoids contents of *B. pilosa* and *E. crus-galli* ranged from 15.98 to 73.00% compare with control. Compared with *P. nigrum*, *P. cubeba* EO decreased *B. pilosa* and *E. crus-galli* pigment more effective by 49.84 and 48.24% in chlorophyll a; 41.76 and 48.79% in chlorophyll b; 47.26 and 48.41% in total chlorophylls; 49.43 and 46.44% in carotenoids contents respectively.

#### 2.3.2. Electrolyte Leakage

**Figure 2:**
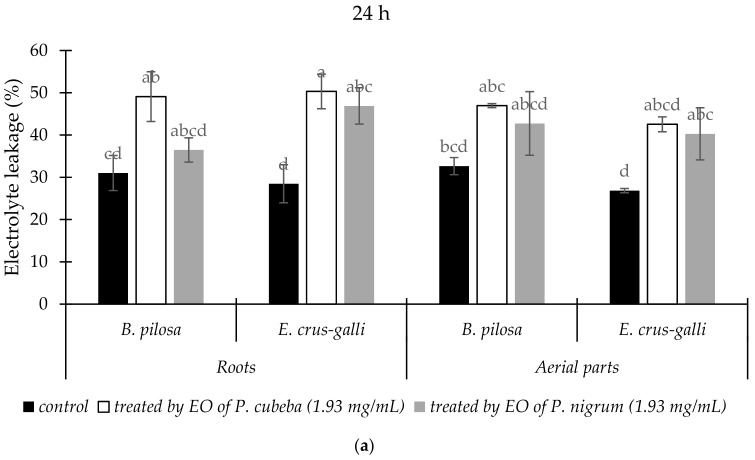

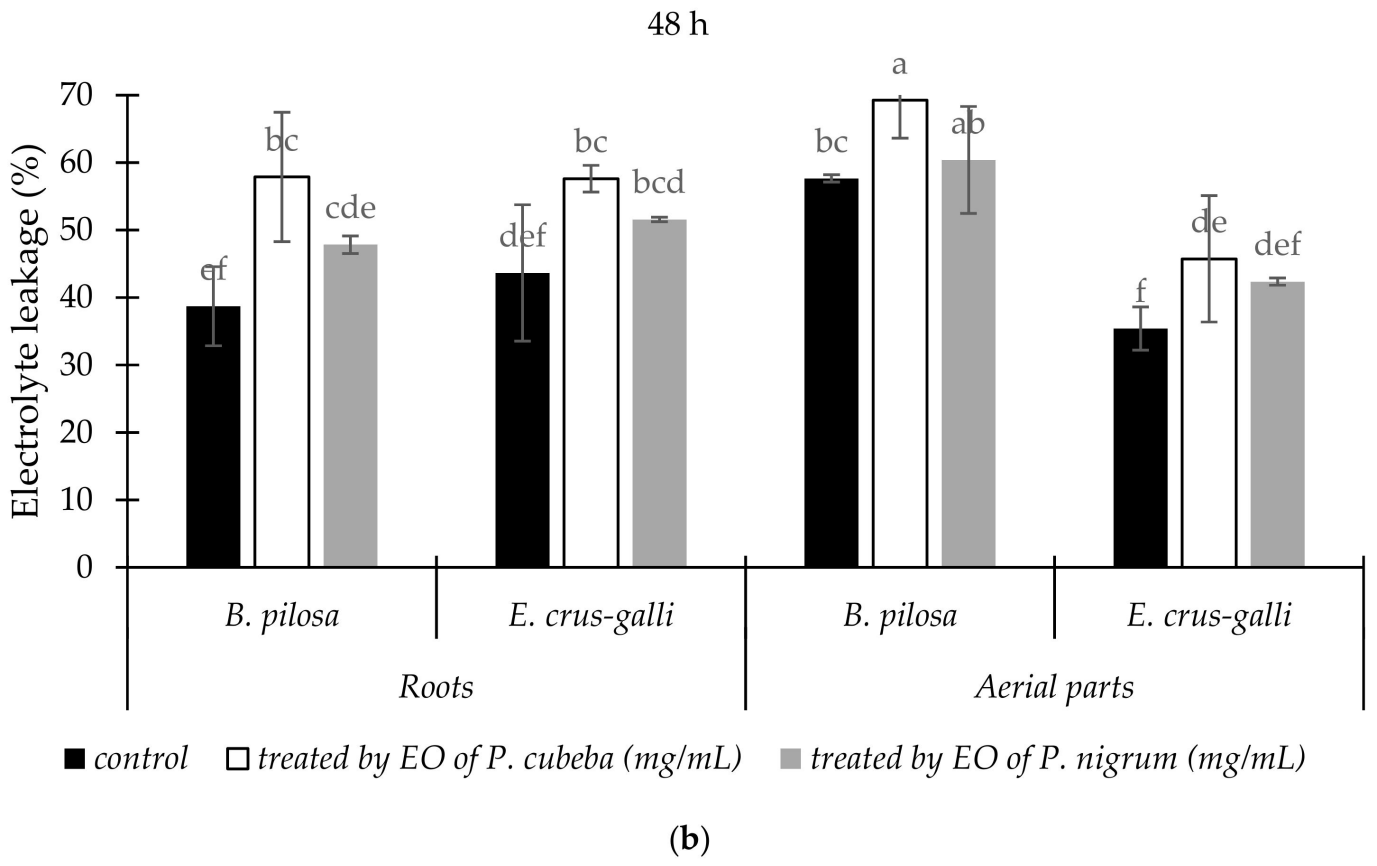
The changes of electrolyte leakage (%) of *B. pilosa* and *E. crus-galli* between control and treatments after 24 h (**a**) and 48 h (**b**). Data were presented in means ± standard deviations (SD). Means with different small letter indicted significantly different by Fisher’s test (*p* < 0.05).

The variation of electrolyte leakage (EL) from the roots and aerial parts of treated and untreated *B. pilosa* and *E. crus-galli* by EOs were presented in Figure 2. Generally, EOs treatments increased EL percentage compared with control. Compared with *P. nigrum*, *P. cubeba* EO gave more effects in increasing of electrolyte leakage percentage in the roots and aerial parts of *B. pilosa* than *E. crus-galli* after 24 h and 48 h treatments. The leakages were greater after 48 h, compared with 24 h.

#### 2.3.3. Lipid Peroxidation

**Figure 3:**
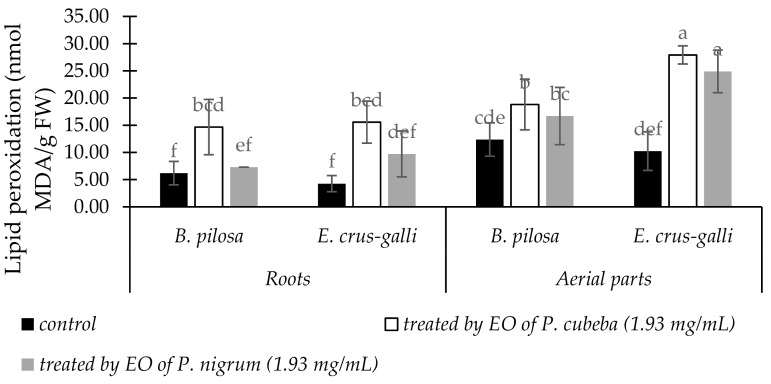
The changes of MDA content between treated and untreated of *B. pilosa* and *E. crus-galli* by EOs. Data were presented in means ± standard deviations (SD). Means with different small letter indicted significantly different by Fisher’s test (*p* < 0.05).

The responses of *B. pilosa* and *E. crus-galli* to *P. cubeba* and *P. nigrum* EOs treatments in lipid peroxidation accumulations were presented in Figure 3. Lipid peroxidation, expressed as malondialdehyde (MDA) contents, were increased significantly in both EOs treatments compared with control. Accumulation of MDA in *E. crus-galli* was the highest as responses of EOs treatments. Compared with *P. nigrum* EO, *P. cubeba* EO gave more stimulation effects in MDA accumulation both in *B. pilosa* and *E. cruss-galli*. *P. cubeba* EO induced MDA accumulation both in *B. pilosa* and *E. crus-galli* by 37.46 and 50.18% in the roots and 11.26 and 10.82% in the aerial parts respectively.

#### 2.3.4. Total Phenolic Contents

**Figure 4:**
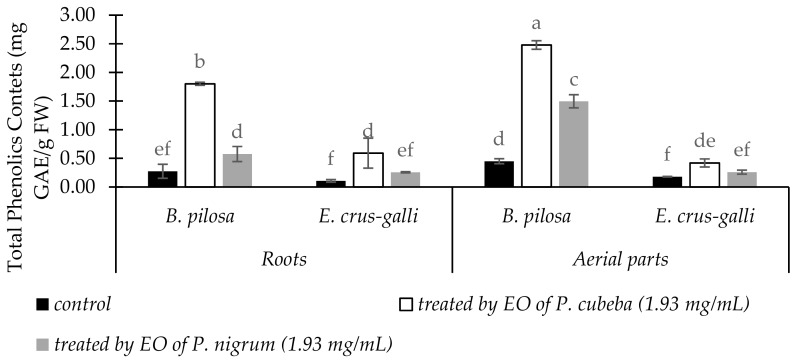
The changes of TPC in the roots and aerial parts of between treated and untreated of *B. pilosa* and *E. crus-galli* by *P. cubeba* and *P. nigrum* EOs. Data were presented in means ± standard deviations (SD). Means with different small letter indicted significantly different by Fisher’s test (*p* < 0.05).

Total phenolic contents of *B. pilosa* and *E. crus-galli* in the roots and aerial parts as response of EOs treatments were indicated in Figure 4. Compared with control, total phenolic contents (TPC) in the EOs treatments of two indicator plants were increased significantly. *P. cubeba* EO gave more stimulation effect on total phenolic accumulations of *B. pilosa* and *E. crus-galli* by 80.82 and 26.54% in the roots and 21.47 and 50.97% in the aerial parts, respectively than *P. nigrum*.

#### 2.3.5. Total Flavonoid Contents

**Figure 5:**
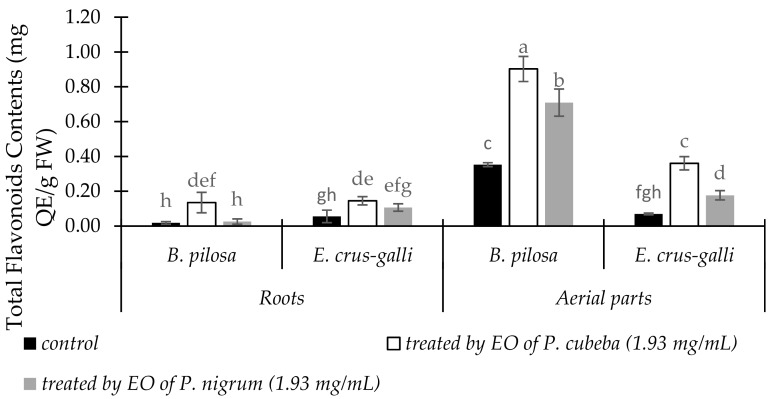
The changes of TFC in the roots and aerial parts of between treated and untreated of *B. pilosa* and *E. crus-galli* by *P. cubeba* and *P. nigrum* EOs. Data were presented in means ± standard deviations (SD). Means with different small letter indicted significantly different by Fisher’s test (*p* < 0.05).

Total flavonoid contents (TFC) in the roots and aerial parts between treated and untreated of *B. pilosa* and *E. crus-galli* were showed in Figure 5. Both in the roots and aerial parts of *B. pilosa* and *E. crus-galli*, TFC were increased significantly when treated by EOs compared with control. The aerial parts of *B. pilosa* showed the maximum response in total flavonoids accumulations. *P. cubeba* EO gave more stimulation of total phenol accumulation for *B. pilosa* and *E. crus-galli* by 68.04 and 56.46% in the roots and 39.67 and 39.19% in the aerial parts respectively than *P. nigrum*.

#### 2.3.6. Proline Contents

**Figure 6:**
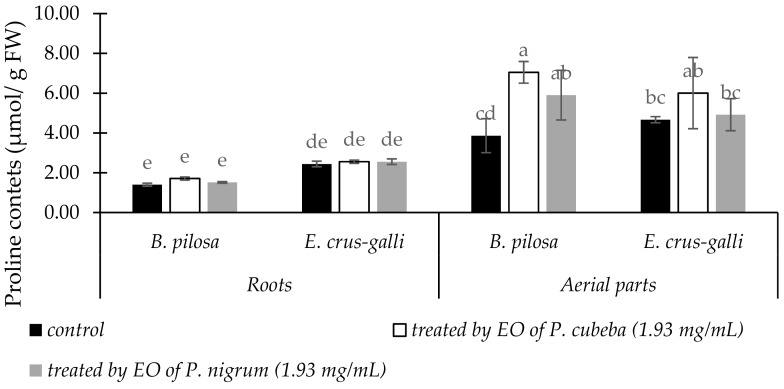
The changes of proline in the roots and aerial parts of between treated and untreated *B. pilosa* and *E. crus-galli* by *P. cubeba* and *P. nigrum* EOs. Data were presented in means ± standard deviations (SD). Means with different small letter indicted significantly different by Fisher’s test (*p* < 0.05).

The responses of *B. pilosa* and *E. crus-galli* between untreated and treated by *P. cubeba* or *P. nigrum* EOs in the proline accumulations were indicated in Figure 6. In the roots, effects of *P. cubeba* and *P. nigrum* EOs in proline accumulation of the two indicator plants were negligible compared with control. In contrast, in the aerial parts proline accumulations between control and treated indicator plants were significantly different. Compare with *P. nigrum*, *P. cubeba* EO stimulated 16.23 and 18.08% higher in proline accumulations of *B. pilosa* and *E. crus-galli* respectively.

#### 2.3.7. Essential Oil Yields

EOs yields of *P. cubeba* and *P. nigrum* were presented in Table 3. The yields of *P. cubeba* and *P. nigrum* EOs were significantly different. *P. cubeba* yielded more EO (1.23% *w*/*w*) compared with *P. nigrum* (1.11% *w*/*w*).

#### 2.3.8. Chemicals Composition of *P. cubeba* and *P. nigrum* Essential Oils

The chemical components of EOs from *P. cubeba* and *P. nigrum* by GC-MS and confirmed by LC-ESI-MS were indicated in Table 4 (Supplementary Materials Figures S1–S24 and Tables S1–S22). Both of EOs contained a complex mixture mainly consisting mainly of monoterpenes and sesquiterpenes.

The major compounds of *P. cubeba* EO were terpinen-4-ol (42.41%), followed by α-copaene (20.04%), γ-elemene (17.68%), α-cubebene (6.54%), and D-germacrene (2.50%). Whereas *P. nigrum* EO contained the dominant components of β-caryophyllene (51.12%), β-thujene (20.58%), β-selinene (5.59%), δ-eIemene (5.03%), and α-copaene (4.79%).

## 3. Discussion

The safety and environmentally friendly of natural substances used in drugs, health foods, or natural herbicides are the major concern of many researchers. Several studies have explored the biological activities of *P. cubeba* or *P. nigrum* EOs such as antioxidant [12,27], anti-larvicidal [10], and insecticidal activities [28]. This study is the first report in comparison of the antihyperuricemic and herbicidal activities of *P. cubeba* and *P. nigrum* EOs. Our study demonstrated that *P. cubeba* and *P. nigrum* EOs possessed antioxidant, antihyperuricemic, and herbicidal activities.

In the antioxidant assay, *P. cubeba* EO had more anti-radical scavenging activity against DPPH and ABTS than *P. nigrum*. However, compared with BHT as positive control, both of the EOs exhibited lower antioxidant capacities. The GC-MS and LC-ESI-MS analyses indicated that terpinen-4-ol (42.41%), α-copaene (20.04%), and γ-elemene (17.68%) were the major components of *P. cubeba* EO. In line with our results, terpinen-4-ol has been reported to have antioxidant activities [4,29]. Whilst for *P. nigrum* EO, β-caryophyllene (51.12%) and β-thujene (20.58%) accounted as the main components. β-caryophyllene is a compound belongs to sesquiterpenes group. This compound has also been reported to possess antioxidant activity [4]. Antioxidant activity of *P. nigrum* has been reported for its methanol, ethanol, and water extracts, and essential oil [12,26]. However, for *P. cubeba*, to the best of our knowledge only methanol, ethanol, and water extracts have been reported [12], while antioxidant activity of its essential is remain unknown. Antioxidant properties of *P. nigrum* and *P. cubeba* EOs in this study may be attribute to the presence of terpinen-4-ol and β-caryophyllene, respectively. Thus, our results suggest that *P. cubeba* and *P. nigrum* EOs may be useful for anti-oxidative stress treatment.

In the antihyperuricemic activitiy, *P. cubeba* and *P. nigrum* EOs possessed ability to inhibit xanthine oxidase. However, the inhibitory effects of the two EOs were lower than allopurinol (20.45 µg/ mL) as positive control of xanthine inhibitory assay. Compared with *P. nigrum*, *P. cubeba* EO (IC_50_ = 54.87 µg/mL) had more inhibitory effect. Inhibitory effects of EOs on xanthine oxidase have been reported from several plants for example *Alpinia zerumbet* [4], rice (*Oryza sativa*) leaf [30], and *Foeniculum vulgare* [31]. In line with our study, Tu and Tawata [4] reported the presence of monoterpenes and sesquiterpenes may be responsible to antyhiperuricemic property of EOs.

Allelopathic effects of water extract of *P. nigrum* leaves against several indicator plants such as *Phaseolus radiatus*, *Raphanus sativus*, *Stylosanthes guianensis*, and *Amaranthus spinous* have been reported. This water extract affected some antioxidant enzymes including superoxide dismutase (SOD), catalase (CAT) and peroxidase (POD) of *Rhapanus sativus*. It also stimulated malondiadehyde (MDA) accumulation of *R. sativus* seedling [32,33]. Furthermore, *P. nigrum* leaching has been noted to possess allelopathic effects on *Vigna mungo* [34]. However, both of *P. cubeba* and *P. nigrum* EOs have been not studied yet.

In the herbicidal assay, *B. pilosa* and *E. crus-galli*, two of the most problematic weeds, were employed as indicator plants. *P. cubeba* and *P. nigrum* EOs showed inhibitory effects on germination and growth of two indicator plants. Compared with *P. nigrum* EO, *P. cubeba* EO exhibited more inhibitory effects. In agreement with our results, several herbicidal activities of EOs have been attributed to the presence of flavonoids, saponins [1], sesquiterpenes [3], limonene, limonene-10-al [35], ocimenones, and spathulenol [36]. Some commercial oils such as limonene and caryophyllene oxide have reported to possess phytotoxic activity [37].

Allelochemicals from EOs generally affected chlorophyll synthesis. In our study, both of *P. cubeba* and *P. nigrum* EOs decreased chlorophyll a, b, total chlorophylls, and carotenoids contents of *B. pilosa* and *E. crus-galli*. *P. cubeba* EO reduced pigments contents by 41.76 to 49.43% stronger than *P. nigrum* at concentration 1.93 mg/mL. These pigments reduction suggests that *P. cubeba* and *P. nigrum* EOs inhibited photosynthesis, due the loss of chlorophylls concentration in two of indicator plants. These results are similar with previous studies which reported that EOs reduced chlorophylls and carotenoid contents [1,38]. The reduction of chlorophylls and carotenoids contents may be because of the changes of either pigments biosynthesis or inhibition of enzyme protoporphyrinogen oxidase [39].

*P. cubeba* and *P. nigrum* EOs increased electrolyte leakage and MDA contents of *B. pilosa* and *E. crus-galli* at 1.93 mg/mL dose of treatments. This condition indicated that membranes of the two indicator plants were disrupted and loss of their integrity [40]. Our results agree with previous studies which reported that *Artemesia scoparia* and *Tagetes erecta* EOs induced electrolyte leakage and MDA accumulation [1,41]. Monoterpenes compounds have been reported to disrupt cell membrane permeability that stimulate MDA accumulation. Consequently, cellular potassium was leakage and inhibited glucose-dependent respiration [42].

Proline accumulations in *B. pilosa* and *E. crus-galli* increased when treated with *P. cubeba* and *P. nigrum* EOs (1.93 mg/mL) compared with control. Proline is a proteinogenic amino acid used in the biosynthesis of proteins. This amino acid accumulation is used for common physiological indicator in many plants in response to a wide range of biotic and abiotic stresses, for example, allelochemical stress. Increasing proline accumulation in *B. pilosa* and *E. crus-galli* may be a part of the stress signal influencing adaptive response due to allelochemicals from *P. cubeba* and *P. nigrum* EOs [40].

During stress, plants release secondary metabolites such as phenolics and flavonoids compounds. In our results, total phenolics and flavonoids contents were increased significantly in *B. pilosa* and *E. crus-galli* when treated by *P. cubeba* and *P. nigrum* EOs compared with control. Similar to our results, Ladhari et al. [43] reported that lettuce enhanced accumulation of total phenolic and flavonoid contents as an adaptive response to allelochemicals from *Cleome arabica*.

The GC-MS and LC-ESI-MS analyses revealed that monoterpenes and sesquiterpenes were the major components of *P. cubeba* and *P. nigrum* EOs. Bos et al. [17] reported the main components of *P. cubeba* EO extracted from its berries were β-elemene (9.4%) and sabinene (9.1%). In contrast, we detected terpinen-4-ol (42.41%), α-copaene (20.04%), and γ-elemene (17.68) as the major components of *P. cubeba* EO. γ-Elemene is isomere of β-elemene, whereas sabinene was also detected in our study as trace element (<0.02%) (data not shown). In case *P. nigrum* EO, β-caryophyllene (51.12%) was the dominant compound detected. Similarly, Morshed et al. [22] and Bagheri et al. [27] reported β-caryophyllene was the main compound of *P. nigrum* EO. The changes of chemical components of EOs may have resulted from several factors such as environmental (climatic, geographic or seasonal) and genetic differences.

## 4. Materials and Methods

### 4.1. Chemicals

Potassium phosphate monobasic and dibasic, Folin-Ciocalteu’s phenol, xanthine oxidase, xanthine, allopurinol, dimethyl sulfoxide (DMSO), hydrochloric acid, tricholoroacetic acid (TCA), Tween 20, thiobarbituric acid (TBA), proline, sulfosalicylic acid, glacial acetic acid, ninhydrin, and toluene were obtained from Sigma-Aldrich Japan K.K. (Tokyo, Japan). Sodium acetate, acetic acid, aluminium (III) chloride hexahydrate, 1,1-diphenyl-2-picrylhydrazyl (DPPH), dibutyl hydroxytoluene (BHT), and gallic acid were purchased from Kanto Chemical Co. Inc. (Tokyo, Japan). Acetone, potassium peroxodisulfate, 2,2′-azinobis (3-ethylbenzothiazoline-6-sulfonic acid) (ABTS) were obtained from Nacalai Tesque, Inc. (Kyoto, Japan). Methanol and ethanol at analytical grade were obtained from Junsei Chemical Co.; Ltd. (Tokyo, Japan).

### 4.2. Plant Materials and Seeds

Berries of *Piper cubeba* Bojer were purchased from a traditional market in Purworejo, Indonesia in 2016 and authenticated by Herbarium Bogoriense, Bogor, Indonesia. The samples were cleaned with tap water, sterilized and dried in an oven at 60 °C for three days before pulverized into a fine powder. The plant powder was then stored in sealed containers at 4 °C until used. Black pepper (*Piper nigrum* L.) powder was purchased commercially in Japan (Gaban, Co. Ltd.; Tokyo, Japan). The seeds of *Bidens pilosa* and *Echinochloa crus-galli* were collected in paddy fields on July 2017 in Higashi Hiroshima, Japan. The seeds germination was randomly tested and was >80% germination.

### 4.3. Essential Oils Extraction

An amount of 300 g of dry powders of *P. cubeba* or *P. nigrum* was hydro-distilled with 2 L of water in a glass Clevenger-type apparatus for seven hours, as recommended by European Pharmacopoeia (ver. 8.2, Monograph 2.8.12). The essential oils collected were diluted with hexane to approximately 10 mL, and vacuum evaporated at the ambient condition to obtain the neat oil. The EOs was then accurately weighed and stored at 4 °C for further analysis. Measurements were carried out in three replications. Essential oil yields (%) were determined using the equation as follows [44]:

(1) $$\mathrm{Essential}\;\mathrm{oil}\;\left(\mathrm{EO}\right)\left(\%\right)=\;\frac{\mathrm{Mass}\;\mathrm{of}\;\mathrm{EO}\;(g)}{\mathrm{Dried}\;\mathrm{materials}\;(g)}\;\times 100$$

### 4.4. Antioxidant Assays

#### 4.4.1. DPPH Radical Scavenging Assay

To evaluate the radical scavenging activity of *P. cubeba* and *P. nigrum* EOs, the 1,1-diphenyl-2-picryhydrazyl (DPPH) test was conducted following the method described previously [30]. Initially, samples or standard (80 µL), DPPH solution (40 µL, 0.2 mM), and acetate buffer (80 µL, 0.1 M, pH 5.5) were mixed in a 96-wells microplate and kept at room temperature in the dark condition for 30 min. The absorbance was recorded at 517 nm using a microplate reader (Multiskan^TM^ Microplate Spectrophotometer, Thermo Fisher Scientific, Osaka, Japan) and butylated hydroxytoluene (BHT) (10–100 µg/mL) was used as a positive control. Percentage of inhibition was calculated as the following formula:

(2) $$\mathrm{Radical}\;\mathrm{scavenging}\;(\%)=\frac{\left(A_{\mathrm{control}}{\;-\;A}_{\mathrm{sample}}\right)}{A_{\mathrm{control}}}\;\times \;100$$

The term A_control_ is the absorbance of reaction without sample and A_sample_ is the absorbance of the reaction mixture with the sample. Antioxidant activity was expressed as IC_50_ values (mg/mL), the concentrations required to give 50% DPPH radical scavenging activity.

#### 4.4.2. ABTS Radical Scavenging Assay

A solution of 2,2′-azinobis (3-ethylbenzothiazoline-6-sulfonic acid) radical cation (ABTS) was employed to evaluate the radical scavenging activity of EOs according to a previously described method [45] with minor adjustments. The ABTS working solution was generated by a reaction of 7 mM ABTS and 2.45 mM potassium persulfate solution after incubation at room temperature in the dark for 16 h. The mixture was then diluted with methanol until obtain an absorbance of 0.70 ± 0.05 at 734 nm. Initially, the ABTS working solution (120 µL) was added to samples (24 µL) or standard containing different concentration. Finally, the mixture was incubated in the dark at ambient condition for 30 min. The absorbance was recorded at 734 nm using microplate a reader (Multiskan^TM^ Microplate Spectrophotometer, Thermo Fisher Scientific, Osaka, Japan). BHT standard (5–125 µg/mL) was used as a positive control. The calculation of ABTS radical scavenging activity was the same with DPPH method.

### 4.5. Xanthine Oxidase Inhibitory Assay

The xanthine oxidase (XO) inhibitory activity was assayed spectrophotometrically in vitro under aerobic condition according to the method reported previously [46,47]. Briefly, a volume of 50 µL sample or standard was mixed with 35 µL of 70 mM phosphate buffer (pH = 7.5), and 30 µL of fresh xanthine oxidase solution (0.01 units/mL in 70 mM phosphate buffer, pH = 7.5) in a 96-well microplate. After pre-incubated at 25 °C for 15 min, the reaction was then initiated by adding 60 µL of substrate solution (150 µM xanthine in the same buffer) and incubated in the same temperature for 30 min. To stop the reaction, a volume of 25 µL HCl (1 M) was added. The absorbance was recorded at 290 nm with a microplate reader. For the blank, the assay mixture was prepared in its present condition, but the enzyme solution was pipetted after adding HCl. One unit of XO was defined as the amount of enzyme required to produce one µmol of uric acid/min at 25 °C. The XO inhibitory activity was calculated as follows:

(3) $$\mathrm{Inhibition}\;(\%)=\;\left\{\frac{\left(A-B\right)-\left(C-D\right)}{A-B}\right\}\times 100$$

where A is the absorbance of the mixture assay without test sample, B is the control of A without test sample and enzyme, C and D are the absorbances of test sample with and without XO, respectively. Allopurinol (10–100 µg/mL) was used as a positive control. The IC_50_ values were calculated from the mean values of percentage inhibition data.

### 4.6. Herbicidal Assays

The herbicidal assays were conducted according to the method reported previously [39]. *P. cubeba* and *P. nigrum* EOs were dispersed in Tween 20 and subsequently diluted with distilled water to obtain several concentrations of samples (final concentration of Tween 20 < 0.02%). A volume of 300 µL of sample was pipetted in a 12-well plate (22.1 mm diameter × 35 mm) lined with filter paper. The seeds of *B. pilosa* and E. *crus-galli* were sterilized with sodium hypochlorite (5%) for 5 min, rinsed by distilled water, and placed in the well plate. The well plates were then kept in a growth chamber (Biotron NC system, Nippon Medical & Chemical Instrument, Co. Ltd.; Osaka, Japan) and additional volume of 50 µL distilled water was added subsequently at 2 to 5 days. Photoperiod was set up 12/12 h day/night with temperature 28/25 °C. After five days, germination percentages, and root and shoot lengths over the controls were recorded and expressed as the percentage of inhibition. The IC_50_ value presented the concentration required to inhibit 50% of germination, shoot height, and root length was also calculated.

### 4.7. Physiological and Biochemical Responses

#### 4.7.1. Chlorophyll and Carotenoid Contents

The chlorophylls (a, b, and total chlorophylls) and carotenoids were determined following the method reported previously [48]. An amount of 100 mg fresh weight leaves of 5-days old *B. pilosa* or *E. crus-galli* seedlings was placed in a microtube, ground, and added 1.5 mL of 80% acetone. After centrifugation at 15,000 rpm, the supernatant of the mixture was recorded at 663, 645 nm and 440 nm using a microplate reader (Multiskan^TM^ Microplate Spectrophotometer). The pigment contents were expressed as µg/mg fresh weight and calculated following the equations:

Total chlorophyll (mg/g) = 20.2 A645 + 8.02 A663(4)

Chlorophyll a (mg/g) = 12.7 A663 − 2.69 A645(5)

Chlorophyll b (mg/g) = 22.9 A645 − 4.68 A663(6)

Carotenoids (mg/g) = (4.7 A440 − (1.38 A663 + 5.48 A645))(7)

#### 4.7.2. Electrolyte Leakage

The electrolyte leakage (EL) was determined by the method described previously [49]. The amount of 100 mg freshly leaves or aerial parts of *B. pilosa* and *E. crus-galli* seedlings (control or treatment) were cut and floated in a tube containing 15 mL of distilled water. The tubes were kept at ambient condition for 24 and 48 h and the initial electrical conductivity of the medium (EC_1_) was measured with a digital conductivity meter (EC Meter CM-14P, TOA Electronics Co.; Ltd.; Nagoya, Japan). After that, the samples were autoclaved at 121 °C for 20 min to release all electrolytes, cooled down to 25 °C, and the final electrical conductivity (EC_2_) were measured. The electrolyte leakage (EL) was calculated according to the following formula [50]:

(8) $$\mathrm{Electrolyte}\;\mathrm{Leakage}\;\left(\%\right)=\left(\frac{{\mathrm{EC}}_{1}}{{\mathrm{EC}}_{2}}\right)\;\times 100$$

#### 4.7.3. Lipid Peroxidation

The lipid peroxidation was estimated by measuring the amount of malondialdehyde (MDA) accumulation according to the method described previously [51]. Fresh samples of roots and aerial parts of *B. pilosa* or *E. crus-galli* (100 mg) were ground and homogenized in 1.5 mL of 0.1% trichloroacetic acid (TCA). Afterward, the homogenate was centrifuged at 15,000 rpm and 4 °C for 20 min. The supernatant (250 µL) was transferred to a microtube and added 750 µL 0.5% thiobarbituric acid (TBA) in 20% TCA. The mixture was then incubated at 90 °C for 10 min in a dry bath incubator (MS-100 Thermo Shaker Incubator, On Wing Tat Co. Ltd.; Kowloon, Hong Kong) and cooled in an ice bath for five min. After centrifugation at 10,000 rpm and 4 °C for 5 min, the absorbance of supernatant was recorded at 532 and 600 nm by using a microplate reader (Multiskan^TM^ Microplate Spectrophotometer). The MDA accumulation was calculated using the Δ (A532 − A600) equation and an extinction coefficient (ε = 155 m/M/cm). The results were expressed as nmol MDA/g fresh weight by following the equation:

MDA (mM/L) = (A_532_ − A_600_)/ε (9)

#### 4.7.4. Proline Contents

The proline accumulations of *B. pilosa* and *E. crus-galli* were measured according to the method reported previously [52]. Initially, 10 mg of powder of dry roots and aerial parts from the two indicator plants were homogenized with 1.5 mL of 3% sulfosalicylic acid. The homogenate solution was centrifuged at 14,000 rpm for 10 min. The supernatant (250 µL) from each homogenate was mixed with 250 µL glacial acetic acid and 250 µL ninhydrin reagent (1.25 g ninhydrin in 30 mL glacial acetic and 20 mL 6 M H_3_PO_4_) in a fresh tube. After, the tubes were incubated at 100 °C for 1 h in a water bath and they rapidly cooled in an ice bath to stop the reaction. Finally, 500 µL mL of toluene was added to each tube. The absorbance of the pink-red upper phase was recorded at 520 nm against toluene blank. L-Proline (2.5 to 50 µg/mL) was used as a standard.

#### 4.7.5. Total Phenolic Contents

The Folin Ciocalteu (FC) reagent was used to measure total phenolic accumulations of *B. pilosa* and *E. crus-galli* following the method reported previously [53] with minor modifications. A volume of 20 µL of either sample solution (1.0 mg/mL), or gallic acid standard solution (5–25 µg/mL) was pipetted into separate wells of a 96-well microplate. A volume of 100 µL of the FC reagent (10% *v*/*v* in water) was added to each well, thoroughly mixed, and an aliquot of 80 µL sodium carbonate (5% *w*/*v* in water) was then added. The reaction mixture was then incubated at ambient condition for 30 min and the absorbance was read at 765 nm by using a microplate reader. The total phenolic contents were expressed as mg gallic acid equivalent (GAE) per gram of fresh weight (r^2^ = 0.996).

#### 4.7.6. Total Flavonoid Contents

The total flavonoid contents of *B. pilosa* and *E. crus-galli* were assessed by a colorimetric assay as described previously [53]. Briefly, a volume of either 100 µL sample (1 mg/mL) or standard (5–25 µg/mL) was mixed with 100 µL aluminum (III) chloride hexahydrate (2% *w*/*v* in water) in a 96-wells-microplate. After a 15-min incubation at room temperature, the absorbance of the reaction mixture was measured at 430 nm using a microplate reader. The total flavonoid contents were expressed as mg quercetin equivalent (QE) per gram of fresh weight (r^2^ = 0.999).

#### 4.7.7. Identification of Chemical Constituents by Gas Chromatography-Mass Spectrometry (GC-MS)

A volume of 1 µL of each sample was injected into a GC-MS system (JMS-T100 GCV, JEOL Ltd.; Tokyo, Japan). The column was DB-5MS with 30 m in length, 0.25 mm internal diameter, and 0.25 µm in thickness (Agilent Technologies, J & W Scientific Products, Folsom, CA, USA). Helium was chosen as a carrier gas, and the split ratio was 5.0/1.0. The operating condition of GC oven temperature was maintained as follows: the initial temperature was set up at 50 °C without hold time, the temperature was increased at a rate of 10 °C/min up to a final temperature of 300 °C (hold for 20 min). The injector and detector temperatures were set at 300 °C and 320 °C, respectively. The mass range scanned from 29–800 amu. The obtained peak was analyzed using JEOL’s GC-MS Mass Center System version 2.65a.

#### 4.7.8. Liquid Chromatography-Electrospray Ionization-Mass Spectrometry (LC-ESI-MS) Analysis

Chemical composition of *P. cubeba* and *P. nigrum* EOs detected in GC-MS system both for volatile and non-volatile components were confirmed by LC-ESI-MS system (Thermo Fisher Scientific^TM^, LTQ XLTM, Ion Trap Mass Spectrometer, Tokyo, Japan) [47]. The column used for the LC system was a JASCO J-Pak Symphonia C18 (250 mm × 4.6 mm × 5 μm). Two solvents for the mobile phase were 0.1% formic acid in water (solvent A) and 0.1% formic acid in acetonitrile (solvent B). The proportion of these solvents was 30:70 (A:B), flowrate was 0.4 mL/min, volume of sample injection was 5 µL, and operation time was 30 min. For ESI-MS, the flow rate of sheath gas was 60, while auxiliary gas was 20 arbitrary units of sheath gas. The spray voltage was 4.5 kV. The measurements were performed in the positive mode. For positive polarity, Fourier transform mass spectrometry (FTMS)/orbitrap with 60,000 resolutions, and scan ranged from 100–1000 *m*/*z* was applied for mass analysis. For negative polarity, ion trap mass spectrometer (ITMS)/linear ion trap with scan range 115–1000 *m*/*z* was employed [54]. Peak processing was conducted using Thermo Xcalibur Qual Browser software (ThermoScientific^TM^) equipped with NIST MS Library.

#### 4.7.9. Statistical Analysis

The statistical analysis was conducted in one-way ANOVA using Minitab^®^ 16.2.3 (^©^2012 Minitab Inc.; Philadelphia, PA, USA). The results were expressed as means ± standard deviation (SD) values. The significant difference among treatments, controls, and standards were determined by using Fisher’s test with the confidence level of 95% (*p* < 0.05).

## 5. Conclusions

*P. cubeba* and *P. nigrum* EOs are the volatile oils contented rich bioactive sources and healthy values. In this study, we found that these EOs possessed antioxidant, antihyperuricemic, and herbicidal activities. Compared with *P. nigrum* EO, *P. cubeba* EO indicated stronger of antioxidant, antihyperuricemic, and herbicidal activities. In the herbicidal assay, *P. cubeba* decreased chlorophyll a, b, total chlorophylls, and carotenoids contents of *B. pilosa* and *E. crus-galli*, but induced electrolyte leakage, lipid peroxidation, total phenolic, total flavonoid, and proline contents. GC-MS and LC-ESI-MS analyses revealed that *P. cubeba* and *P. nigrum* EOs had complex mixture of compounds that consisted of monoterpenes and sesquiterpenes. Terpinen-4-ol (42.41%), followed by α-copaene (20.04%) and γ-elemene (17.68%) were accounted as the major components of *P. cubeba* EO. Whereas, β-caryophyllene (51.12%) and β-thujene (20.58%) were the dominant components of *P. nigrum* EO. Findings of this study suggested that *P. cubeba* and *P. nigrum* EOs are promising source to treat oxidative stress and antihyperuricemia diseases as well as natural herbicide against *B. pilosa* and *E. crus-galli*.

## Figures and Tables

**Table 1:** Antioxidant and xanthine oxidase inhibitory activities of EOs from *P. cubeba* and *P. nigrum*.

Samples	Antioxidant Activity (IC_50_ (mg/mL))		XOI Activity (IC_50_ (µg/mL))
	DPPH	ABTS	
*P. cubeba* EO	0.82 ± 0.06 *^b^*	1.32 ± 0.04 *^b^*	54.87 ± 1.69 *^a b^*
*P. nigrum* EO	1.15 ± 0.08 *^a^*	1.74 ± 0.03 *^a^*	77.11 ± 2.11 *^a^*
BHT *	0.009 ± 0.02 *^c^*	0.071 ± 0.001 *^c^*	-
Allopurinol **	-	-	20.45 ± 0.3 *^b^*

Data presented means ± standard deviations (SD). Values in the same column followed by similar letters are not significantly different by Fisher’ (*p* < 0.05). * = positive control for antioxidant assay. ** = positive control for xanthine oxidase inhibitory assay. - = not determined.

**Table 2:** Inhibition (IC_50_) values of *P. cubeba* and *P. nigrum* EOs on some indicator plants.

Treatments	Indicator Plants	IC_50_ (mg/mL)		
		Germination	Roots	Shoots
*P. cubeba* EOs	*B. pilosa*	5.19 ± 0.22 *^a^*	2.63 ± 0.58 *^a^*	1.93 ± 0.68 *^b^*
	*E. crus-galli*	5.26 ± 0.34 *^a^*	3.30 ± 0.97 *^a^*	5.86 ± 0.94 *^a b^*
*P. nigrum* EOs	*B. pilosa*	5.44 ± 0.00 *^a^*	2.67 ± 0.64 *^a^*	3.1 ± 0.56 *^a b^*
	*E. crus-galli*	> 8.00	2.71 ± 0.11 *^a^*	7.21 ± 2.02 *^a^*

Data were presented in means ± standard deviations (SD). Different letter in a column indicated significantly different by Fisher’s test (*p* < 0.05).

**Table 3:** Yields of essential oils extracted from *P. cubeba* and *P. nigrum.*

Samples	Dry Weight (g)	EOs (g)	Yields (% *w*/*w*) (% *w*/*w* DW)
*P. cubeba*	300.00	3.69 ± 0.05 *^a^*	1.23 ± 0.01 *^a^*
*P. nigrum*	300.00	3.35 ± 0.01 *^b^*	1.11 ± 0.01 *^b^*

Data were presented in means ± standard deviations (SD). Different letter in a column indicted significantly different by Fisher’s test (*p* < 0.05).

**Table 4:** Identification of chemical components of *P. cubeba* and *P. nigrum EOs* by GC-MS.

No.	Compounds	Rt (min)	Chemical Formula	MW (g/mol)	Chemical Class	Area (%)		RI *
						*P. cubeba EO*	*P. nigrum EO*	
1	β-Thujene	5.32	C_10_H_16_	136.238	Monoterpenes	-	20.58	929
2	Terpinen-4-ol	7.81	C_10_H_18_O	154.253	Monoterpenes	42.41	1.85	1179
3	*trans*-Chrysanthenyl acetate	8.38	C_12_H_18_O_2_	194.274	Monoterpenes	0.1	-	1228
4	δ-EIemene	9.96	C_15_H_24_	204.357	Monoterpenes	0.58	5.03	1338
5	α-Cubebene	10.13	C_15_H_24_	204.357	Sesquiterpenes	6.54	-	1349
6	α-Copaene	10.55	C_15_H_24_	204.357	Sesquiterpenes	20.04	4.79	1377
7	D-Germacrene	10.69	C_15_H_24_	204.357	Sesquiterpenes	2.50	-	1451
8	γ-Elemene	11.21	C_15_H_24_	204.357	Sesquiterpenes	17.68	-	1455
9	β-Caryophyllene	11.23	C_15_H_24_	204.357	Sesquiterpenes	-	51.12	1467
10	Humulene	11.60	C_15_H_24_	204.357	Sesquiterpenes	-	3.81	1474
11	β-Selinene	12.03	C_15_H_24_	204.357	Sesquiterpenes	-	5.59	1490
12	δ-Cadinene	12.33	C_15_H_24_	204.357	Sesquiterpenes	2.7	2.04	1525
13	α-Elemol	12.68	C_15_H_26_O	222.372	Sesquiterpenes	1.78	-	1551
14	Spathulenol	13.07	C_15_H_24_O	220.356	Sesquiterpenes	0.18	-	1581
15	Caryophyllene oxide	13.16	C_15_H_24_O	220.356	Sesquiterpenes	-	1.51	1595
16	Cubenol	13.65	C_15_H_26_O	222.372	Sesquiterpenes	0.44	0.97	1642
17	β-Eudesmol	14.00	C_15_H_26_O	222.372	Sesquiterpenes	0.64	-	1655

Rt = retention time of GC-MS. MW= molecular weight. - = not detected. RI = retention index, * calculated according to Kovat’s index based on NIST Mass Spectral Library [26].

## Supplementary Materials

Figures S1–S24 and Tables S1–S22 are provided. Figures S1 and S13 are GC-MS chromatogram of P. cubeba and P. nigrum EOs. Tables S1–S12 are GC-MS fragmentation pattern, Figures S2a–S13a are GC-MS mass spectra, and Figures S2b–S13b are LC-ESI-MS spectra by H+ ion adduction for terpinen-4-ol, trans-chrysanthenyl acetate, δ-eIemene, α-cubebene, α-copaene, d-germacrene, γ-elemene, δ-cadinene, α-elemol, spathulenol, cubenol, and β-eudesmol detected in P. cubeba EO, respectively. Tables S13–S22 are GC-MS fragmentation pattern, Figures S14a–S24a are GC-MS mass spectra, and Figures S14b–S24b are LC-ESI-MS spectra by H+ ion adduction for β-thujene, terpinen-4-ol, δ-eIemene, α-copaene, β-caryophyllene, humulene, β-selinene, δ-cadinene, caryophyllene oxide, and cubenol detected in P. nigrum EO.
