# Supplementary material for: Biological Activities and Chemical Constituents of Essential Oils from Piper cubeba Bojer and Piper nigrum L
Source: Molecules. 2019 May 15;24(10):1876. doi: 10.3390/molecules24101876 (PMC6571889; doi:10.3390/molecules24101876)
Supplement: Supplementary file 1 [file molecules-24-01876-s001.pdf]

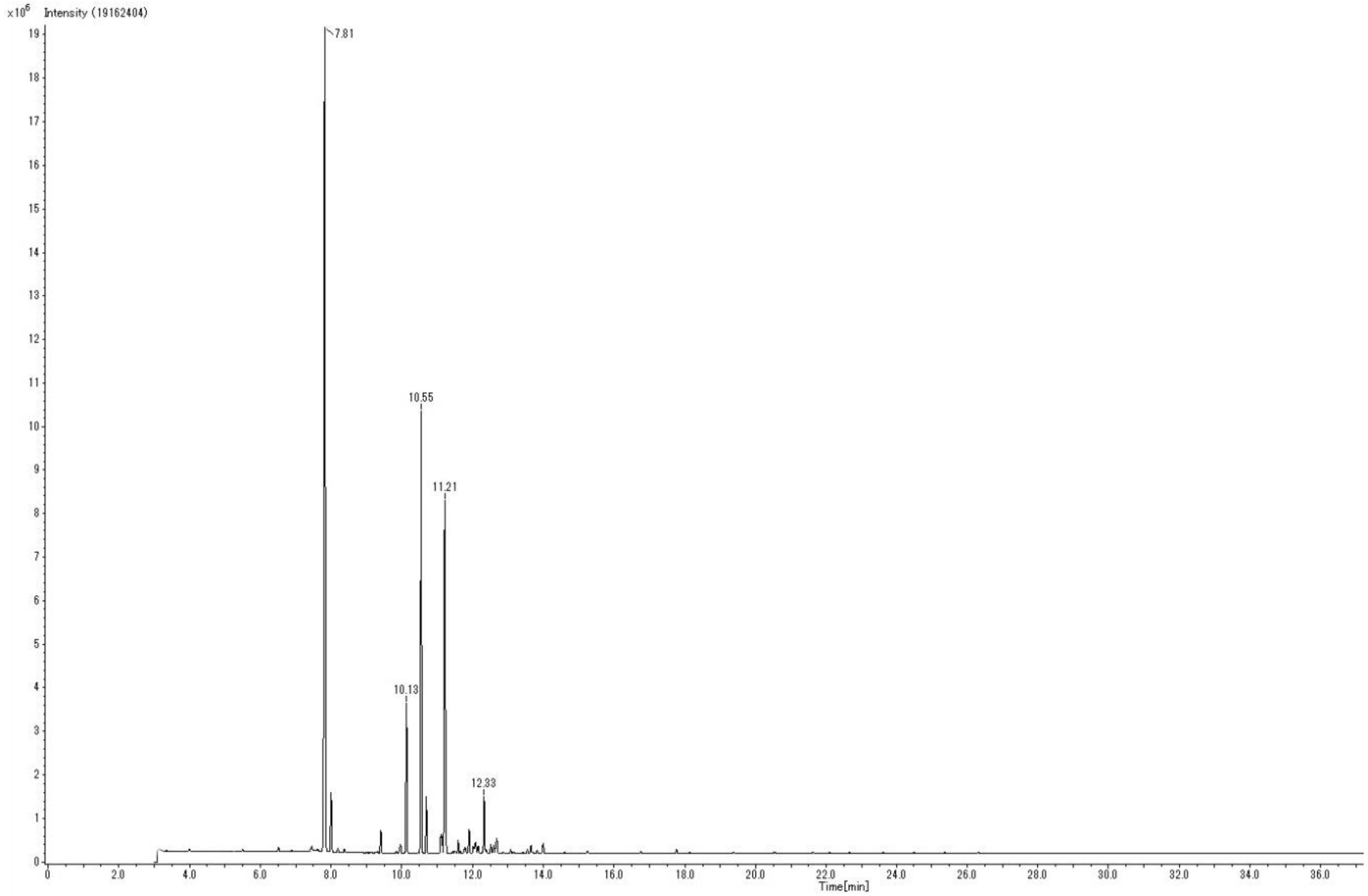

Figure S1. GC-MS Chromatogram of *Piper cubeba* essential oil

Table S1. Fragmentation pattern of Terpinen-4-ol (retention time = 7.81) in *P. cubeba* essential oil detected by GC-MS

Peak#	m/z	Intensity	Relative Intensity (%)	Peak#	m/z	Intensity	Relative Intensity (%)
1	29.013	5369	1.821	54	43.313	727	0.247
2	29.050	25090	8.512	55	43.361	894	0.303
3	29.111	1008	0.342	56	43.395	703	0.238
4	29.263	84	0.028	57	43.428	212	0.072
5	29.290	128	0.043	58	43.576	1489	0.505
6	29.302	61	0.021	59	43.639	514	0.174
7	29.314	154	0.052	60	43.822	125	0.042
8	29.512	52	0.018	61	43.868	438	0.149
9	29.688	87	0.030	62	43.907	194	0.066
10	30.013	109	0.037	63	44.039	4197	1.424
11	30.054	886	0.301	64	44.074	3893	1.321
12	31.030	8748	2.968	65	44.129	382	0.13
13	32.086	571	0.194	66	44.383	47	0.016
14	32.255	54	0.018	67	45.050	8300	2.816
15	35.989	166	0.056	68	45.107	92	0.031
16	37.021	419	0.142	69	46.053	142	0.048
17	38.023	1545	0.524	70	46.984	211	0.072
18	39.038	61990	21.030	71	50.034	2863	0.971
19	39.108	1092	0.370	72	51.042	12641	4.289
20	39.161	219	0.074	73	52.049	7366	2.499
21	39.291	19	0.006	74	53.022	753	0.255
22	39.313	152	0.052	75	53.058	51945	17.623
23	39.336	283	0.096	76	53.127	618	0.21
24	39.547	54	0.018	77	53.168	94	0.032
25	39.558	56	0.019	78	53.319	63	0.021
26	39.582	69	0.023	79	53.365	39	0.013
27	39.806	58	0.020	80	53.374	75	0.025
28	40.045	16022	5.436	81	53.416	71	0.024
29	40.097	353	0.120	82	53.669	90	0.031
30	40.609	84	0.028	83	54.031	109	0.037
31	41.054	138363	46.940	84	54.065	8777	2.978
32	41.113	289	0.098	85	55.037	35951	12.197
33	41.167	698	0.237	86	55.074	49702	16.862
34	41.222	63	0.021	87	55.133	161	0.055
35	41.339	1405	0.477	88	55.207	197	0.067
36	41.368	961	0.326	89	55.340	150	0.051
37	41.542	192	0.065	90	55.407	378	0.128
38	41.591	608	0.206	91	55.687	263	0.089
39	41.627	321	0.109	92	55.715	86	0.029
40	41.802	118	0.040	93	56.043	2622	0.89
41	41.838	186	0.063	94	56.079	3898	1.322
42	41.860	292	0.099	95	57.054	11419	3.874
43	41.885	89	0.030	96	57.090	2876	0.976
44	42.026	4270	1.449	97	57.115	93	0.032
45	42.061	15507	5.261	98	58.062	5846	1.983
46	42.110	682	0.231	99	59.070	4039	1.37
47	42.149	163	0.055	100	60.077	130	0.044
48	42.332	171	0.058	101	62.038	391	0.133
49	42.356	102	0.035	102	63.046	3362	1.141
50	43.033	84801	28.769	103	64.053	1017	0.345
51	43.070	101671	34.492	104	65.062	20072	6.81
52	43.121	287	0.097	105	65.127	610	0.207
53	43.171	1022	0.347	106	65.334	30	0.01

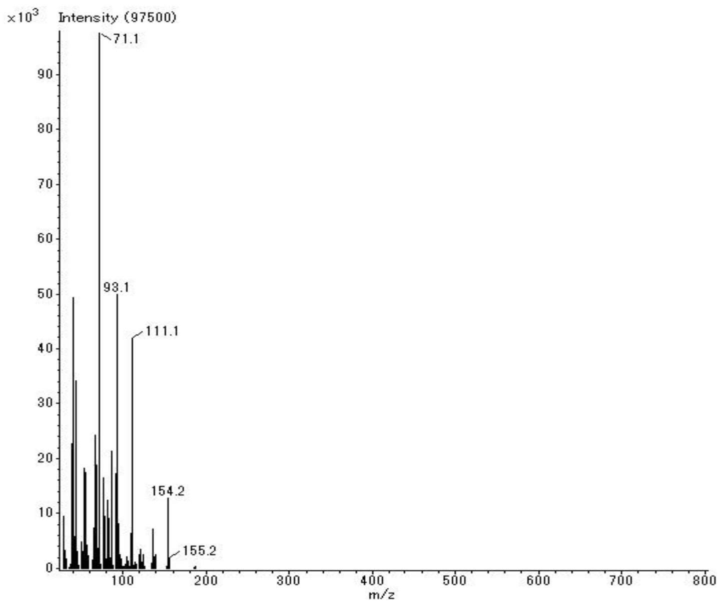

Figure S2a. Mass-spectra of Terpinen-4-ol (Rt= 7.81) detected in *P. cubeba* essential oil by GC-MS

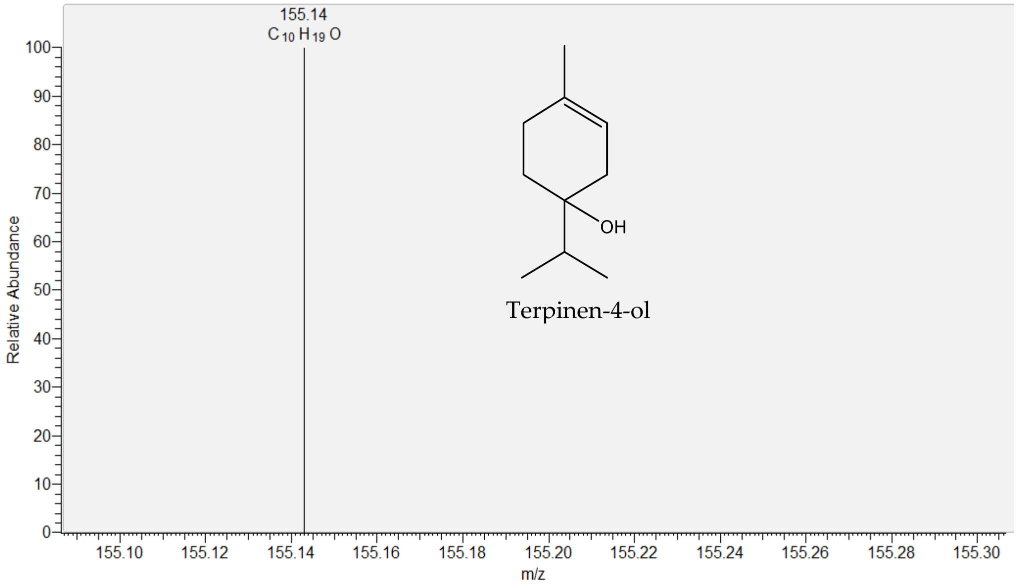

Figure S2b. Spectra of Terpinen-4-ol in *P. cubeba* essential oil identified by positive ion (H^+) adduction method of LC-ESI-MS

Table S2. Fragmentation pattern of trans-Chrysanthenyl acetate in *P. cubeba* essential oil detected by GC-MS

Peak#	m/z	Intensity	Relative Intensity (%)
1	29	451	18.621
2	30	635	26.218
3	31	447	18.456
4	32	527	21.759
5	38	584	24.112
6	39	437	18.043
7	41.1	615	25.392
8	43	2422	100.000
9	44	89	3.675
10	53.1	453	18.704
11	59.1	1653	68.249
12	77.1	966	39.884
13	79.1	1080	44.591
14	85	461	19.034
15	91.1	1154	47.647
16	93.1	955	39.430
17	119.1	2094	86.457
18	120.1	268	11.065
19	134.2	771	31.833
20	135.2	555	22.915

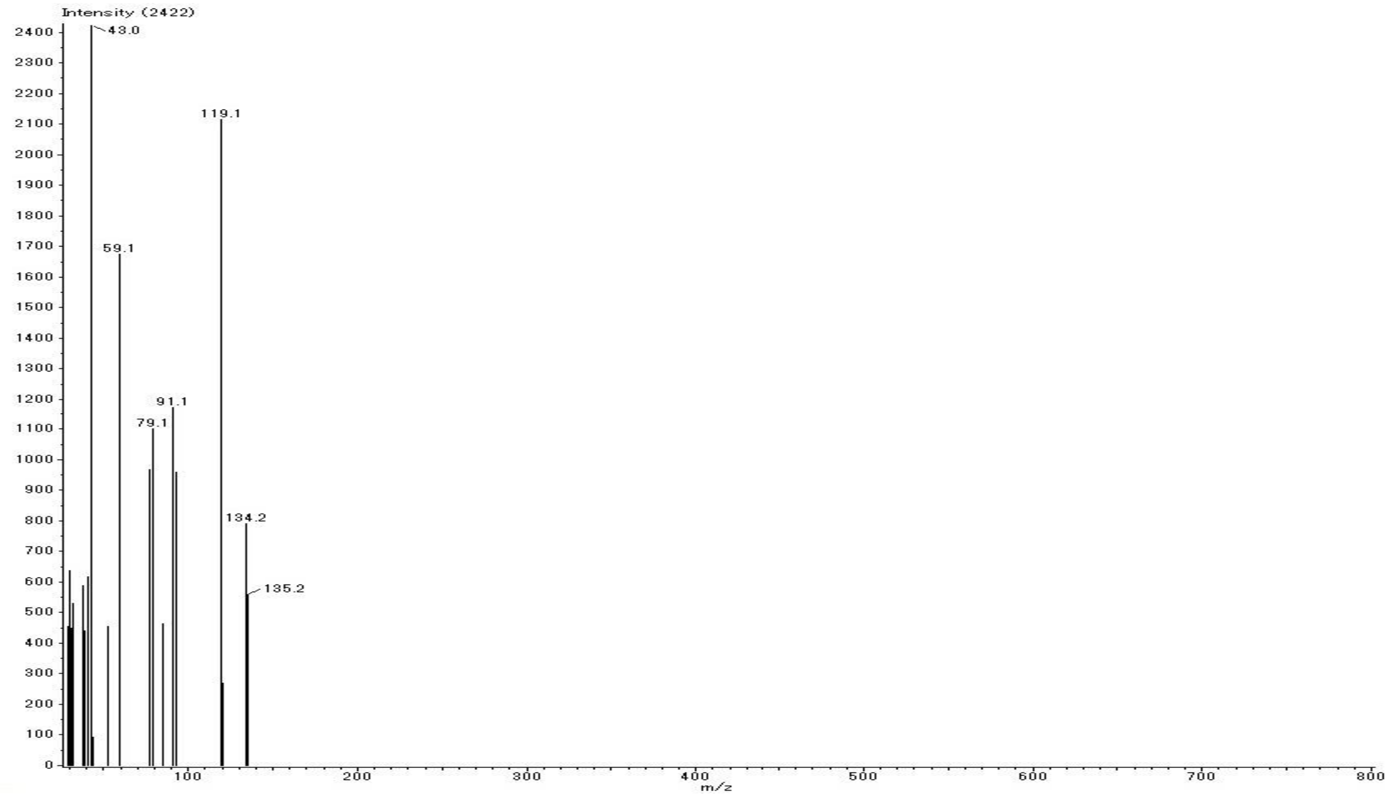

Figure S3a. Mass-spectra of trans-Chrysantheyl acetate (Rt= 8.38) detected in *P. cubeba* essential oil by GC- MS

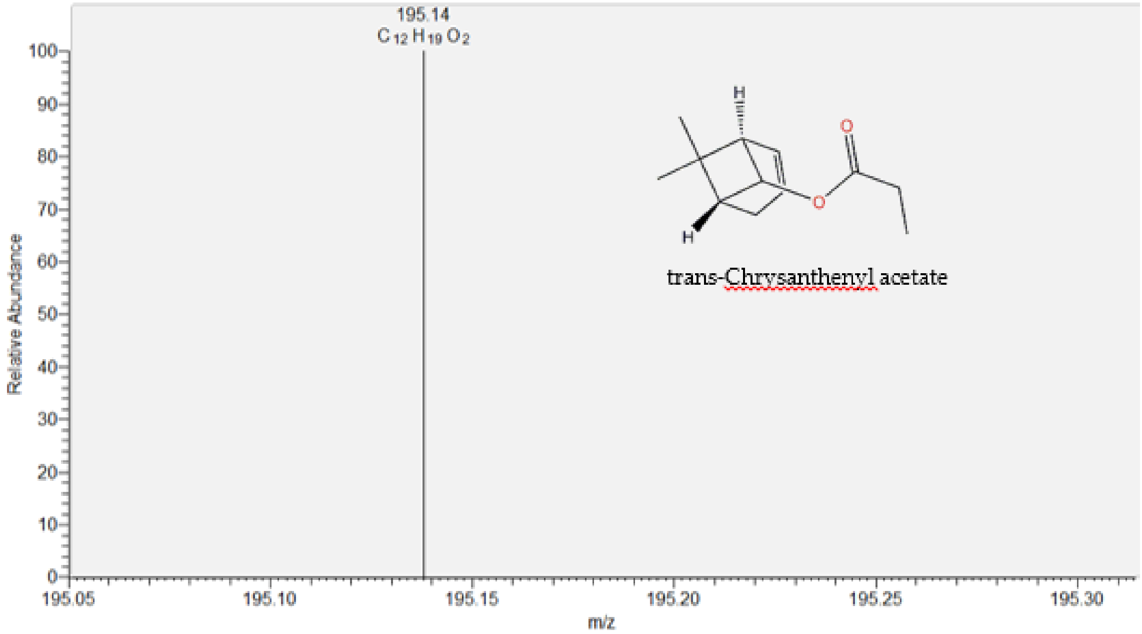

Figure S3b. Spectra of trans-Chrysantheyl acetate in *P. cubeba* essential oil identified by positive ion (H^+) adduction method of LC-ESI-MS

Table S3. Fragmentation pattern of δ-EIemene (retention time = 9.96) in *P. cubeba* essential oil detected by GC-MS

Peak#	m/z	Intensity	Relative Intensity (%)	Peak#	m/z	Intensity	Relative Intensity (%)
1	29	190	3.680	26	78.1	681	13.190
2	29.1	1723	33.372	27	79.1	1977	38.292
3	32	422	8.174	28	80.1	823	15.940
4	32	81	1.569	29	81.1	1348	26.109
5	32.1	225	4.358	30	82.1	208	4.029
6	32.2	47	0.910	31	91.1	3011	58.319
7	34	184	3.564	32	92.1	678	13.132
8	35	209	4.048	33	93.1	4146	80.302
9	38	318	6.159	34	94.1	1453	28.143
10	39	1760	34.089	35	95.1	728	14.100
11	40	300	5.811	36	105.1	1611	31.203
12	41.1	3022	58.532	37	106.1	435	8.425
13	42.1	150	2.905	38	107.1	2531	49.022
14	43.1	908	17.587	39	108.1	700	13.558
15	44	35	0.678	40	119.1	909	17.606
16	51	216	4.184	41	121.1	5163	100.000
17	53.1	1747	33.837	42	122.1	803	15.553
18	54.1	220	4.261	43	123.2	543	10.517
19	55.1	1604	31.067	44	133.1	261	5.055
20	65.1	1026	19.872	45	136.1	47	0.910
21	66.1	111	2.150	46	136.2	1314	25.450
22	67.1	1794	34.747	47	161.2	723	14.003
23	68.1	724	14.023	48	187	233	4.513
24	69.1	314	6.082	49	189.2	162	3.138
25	77.1	1667	32.287	50	482.1	79	1.530

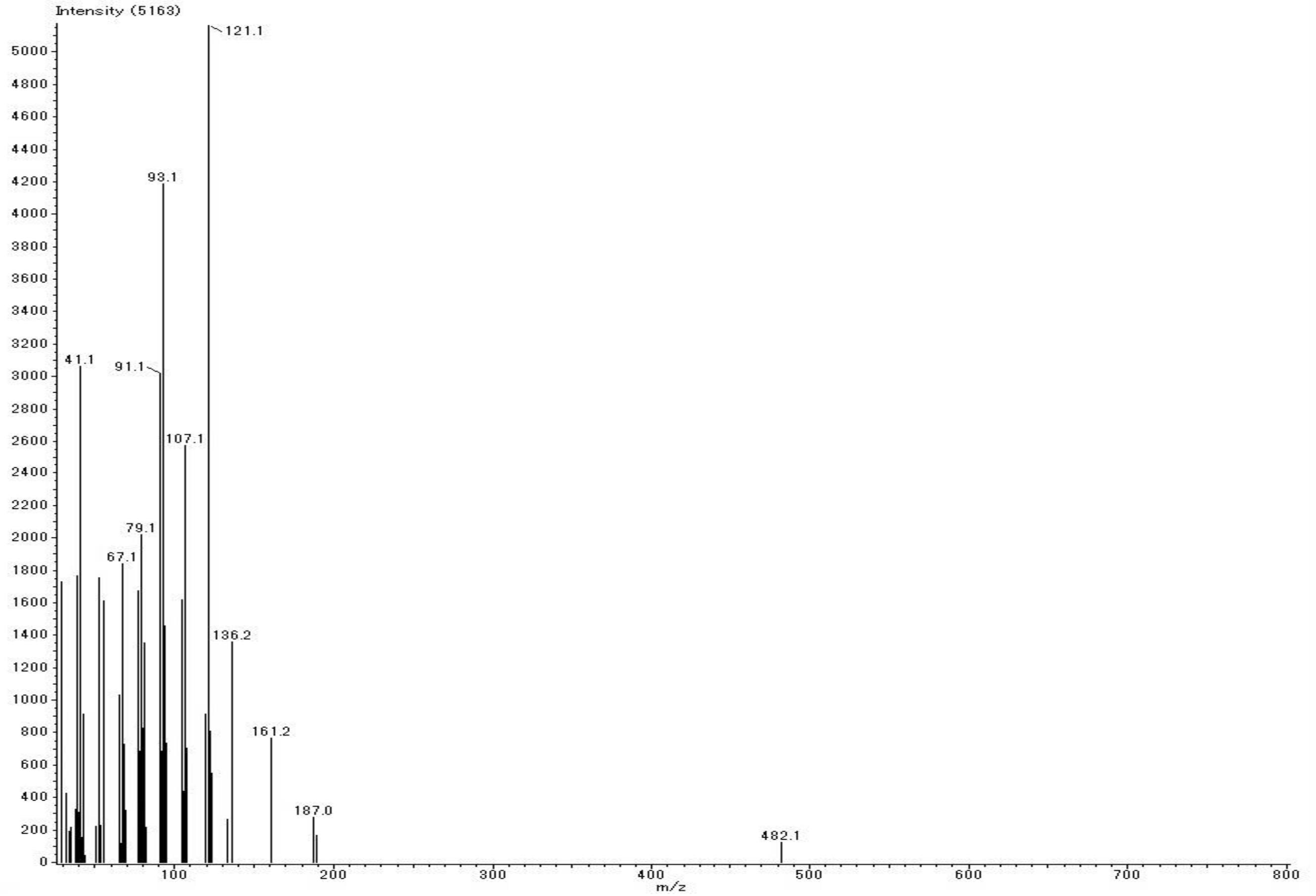

Figure S4a. Mass-spectra of δ-EIemene (Rt= 9.96) detected in *P. cubeba* essential oil by GC- MS

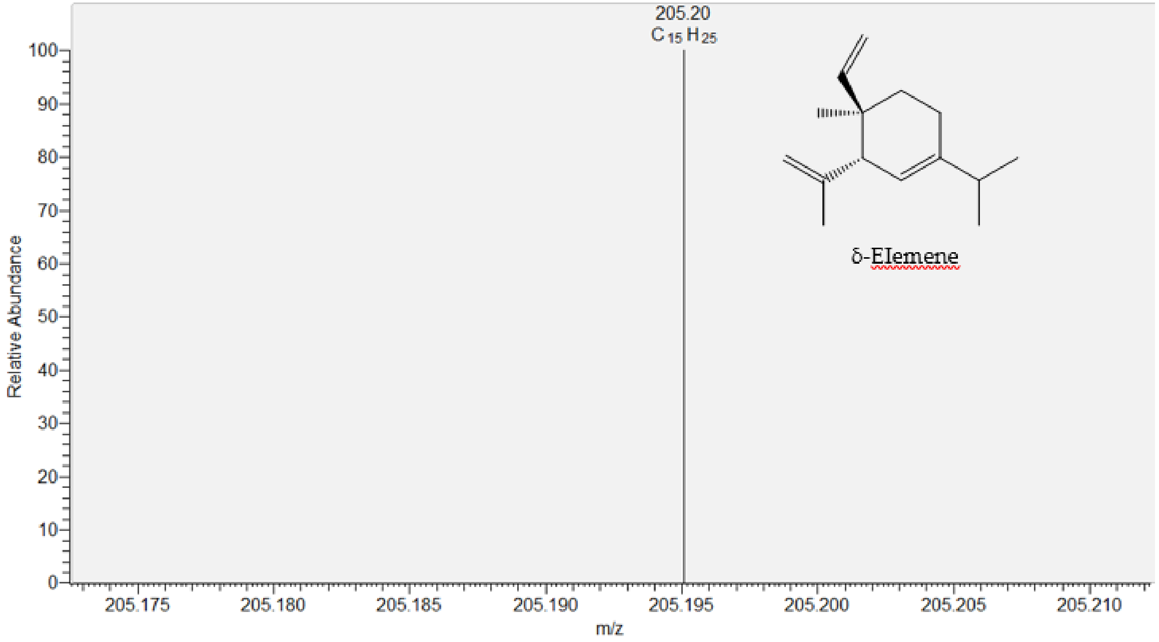

Figure S4b. Spectra of δ-EIemene in *P. cubeba* essential oil identified by positive ion (H^+) adduction method of LC-ESI-MS

Table S4. Fragmentation pattern of α-Cubebene (retention time = 10.13) in *P. cubeba* essential oil detected by GC-MS

Peak#	m/z	Intensity	Relative Intensity (%)	Peak#	m/z	Intensity	Relative Intensity (%)
1	29	65	0.3236	52	104.1	1170	5.8250
2	29	3018	15.0254	53	105.1	20086	100.0000
3	32.1	229	1.1401	54	105.2	525	2.6138
4	34	328	1.6330	55	105.2	211	1.0505
5	38	8	0.0398	56	105.3	154	0.7667
6	39	3078	15.3241	57	106.1	2760	13.7409
7	40	136	0.6771	58	107.1	2933	14.6022
8	40	424	2.1109	59	108.1	1224	6.0938
9	41.1	9254	46.0719	60	109.1	247	1.2297
10	41.1	114	0.5676	61	110.1	118	0.5875
11	41.1	159	0.7916	62	115.1	2443	12.1627
12	42.1	1169	5.8200	63	116.1	876	4.3612
13	43	364	1.8122	64	117.1	3693	18.3859
14	43.1	4515	22.4783	65	118.1	1599	7.9608
15	44	88	0.4381	66	119.1	17528	87.2648
16	44.1	120	0.5974	67	119.2	304	1.5135
17	50	164	0.8165	68	120.1	5317	26.4712
18	51	1022	5.0881	69	121.1	2241	11.1570
19	52	473	2.3549	70	122.1	370	1.8421
20	53.1	2682	13.3526	71	123.2	244	1.2148
21	54.1	208	1.0355	72	127.1	313	1.5583
22	55.1	4727	23.5338	73	128.1	1447	7.2040
23	56.1	1164	5.7951	74	129.1	1281	6.3776
24	57.1	494	2.4594	75	130.1	489	2.4345
25	63	253	1.2596	76	131.1	1722	8.5731
26	64.1	146	0.7269	77	132.1	443	2.2055
27	65.1	2235	11.1272	78	133.1	2156	10.7338
28	66.1	322	1.6031	79	134.2	611	3.0419
29	67.1	1849	9.2054	80	135.2	224	1.1152
30	68.1	236	1.1749	81	141.1	285	1.4189
31	69.1	2490	12.3967	82	142.1	238	1.1849
32	70.1	558	2.7781	83	143.1	247	1.2297
33	77.1	5240	26.0878	84	144.1	270	1.3442
34	78.1	1618	8.0554	85	145.2	1080	5.3769
35	79.1	4300	21.4079	86	146.2	227	1.1301
36	80.1	1045	5.2026	87	147.2	933	4.6450
37	81.1	5180	25.7891	88	148.2	230	1.1451
38	82.1	638	3.1763	89	149.2	221	1.1003
39	83.1	253	1.2596	90	159.2	966	4.8093
40	85	43	0.2141	91	160.2	294	1.4637
41	87.9	75	0.3734	92	161.2	16342	81.3602
42	89.1	319	1.5882	93	161.4	106	0.5277
43	91.1	11533	57.4181	94	162.2	2651	13.1982
44	91.2	319	1.5882	95	163.2	203	1.0107
45	92.1	4483	22.3190	96	178	178	0.8862
46	93.1	6128	30.5088	97	185	206	1.0256
47	94.1	932	4.6400	98	187	25	0.1245
48	95.1	151	0.7518	99	189.2	176	0.8762
49	95.1	887	4.4160	100	204.3	3047	15.1698
50	102.1	271	1.3492	101	205.3	661	3.2908
51	103.1	1999	9.9522				

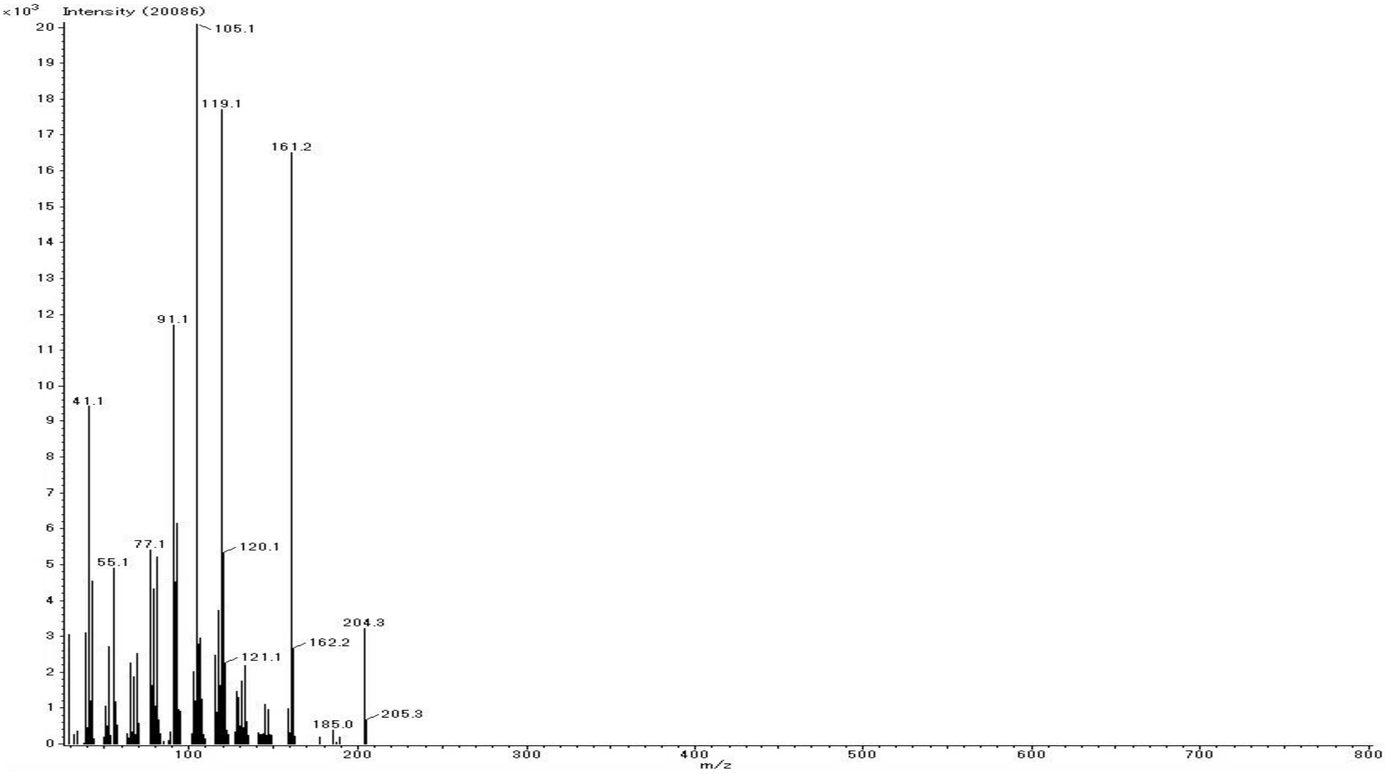

Figure S5a. Mass-spectra of α-Cubebene (Rt= 10.13) detected in *P. cubeba* essential oil by GC- MS

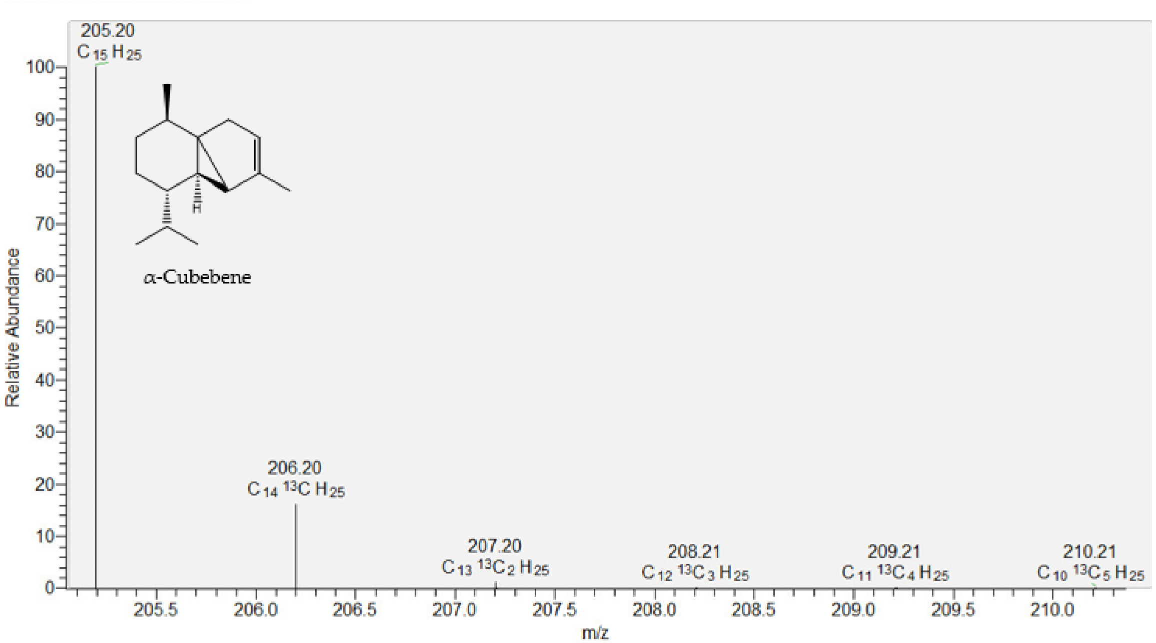

Figure S5b. Spectra of α-Cubebene in *P. cubeba* essential oil identified by positive ion (H^{+}) adduction method of LC-ESI-MS

Table S5. Fragmentation pattern of alpha-Copaene (retention time = 10.55) in *P. cubeba* essential oil detected by GC-MS

Peak#	m/z	Intensity	Relative Intensity (%)	Peak#	m/z	Intensity	Relative Intensity (%)
1	29	202	0.570	62	97.1	304	0.857
2	29	5298	14.940	63	102.1	336	0.948
3	30	84	0.237	64	103.1	2900	8.178
4	32	429	1.210	65	104.1	1609	4.537
5	36	40	0.113	66	105.1	35461	100.000
6	39	5998	16.914	67	105.2	300	0.846
7	40	1089	3.071	68	105.2	308	0.869
8	41.1	20257	57.125	69	106.1	6260	17.653
9	41.1	607	1.712	70	107.1	6084	17.157
10	42.1	1834	5.172	71	108.1	1898	5.352
11	43.1	10090	28.454	72	109.1	790	2.228
12	43.1	73	0.206	73	115.1	4225	11.914
13	44.1	416	1.173	74	116.1	1755	4.949
14	45	93	0.262	75	117.1	5508	15.533
15	50	153	0.431	76	118.1	2268	6.396
16	51	1692	4.771	77	119.1	34608	97.595
17	52	984	2.775	78	119.2	98	0.276
18	53.1	5518	15.561	79	119.3	370	1.043
19	53.1	167	0.471	80	119.6	96	0.271
20	54.1	707	1.994	81	120.1	8768	24.726
21	55.1	9581	27.018	82	120.2	58	0.164
22	55.1	54	0.152	83	121.1	5259	14.830
23	55.1	74	0.209	84	122.1	1348	3.801
24	56.1	1950	5.499	85	123.2	681	1.920
25	57.1	1052	2.967	86	127.1	741	2.090
26	63	537	1.514	87	128.1	2288	6.452
27	64.1	248	0.699	88	129.1	2185	6.162
28	65.1	4590	12.944	89	130.1	1249	3.522
29	66.1	1030	2.905	90	131.1	3918	11.049
30	67.1	4827	13.612	91	132.1	1142	3.220
31	67.1	129	0.364	92	133.1	4843	13.657
32	68.1	806	2.273	93	134.2	2079	5.863
33	69.1	4807	13.556	94	135.2	1102	3.108
34	70.1	969	2.733	95	136.2	3010	8.488
35	71.1	92	0.259	96	137.2	311	0.877
36	76.1	220	0.620	97	141.1	707	1.994
37	77.1	11683	32.946	98	142.1	294	0.829
38	77.1	172	0.485	99	143.1	572	1.613
39	77.1	275	0.775	100	144.1	388	1.094
40	77.2	133	0.375	101	145.2	2163	6.100
41	78.1	3224	9.092	102	146.2	881	2.484
42	79.1	8981	25.326	103	147.2	1774	5.003
43	79.2	119	0.336	104	148.2	963	2.716
44	80.1	2400	6.768	105	149.2	425	1.198
45	81.1	9789	27.605	106	150.2	170	0.479
46	81.2	232	0.654	107	159.2	1220	3.440
47	82.1	911	2.569	108	160.2	809	2.281
48	83	210	0.592	109	161.2	29168	82.254
49	83.1	643	1.813	110	161.3	373	1.052
50	89.1	526	1.483	111	161.4	207	0.584
51	90.1	201	0.567	112	161.8	123	0.347
52	91.1	21674	61.121	113	162.2	4985	14.058
53	91.2	1104	3.113	114	162.3	137	0.386
54	92.1	11834	33.372	115	162.3	105	0.296
55	92.2	207	0.584	116	163.2	378	1.066

Table S5. Cont.

Peak#	m/z	Intensity	Relative Intensity (%)	Peak#	m/z	Intensity	Relative Intensity (%)
56	93.1	19582	55.221	117	175.2	206	0.581
57	93.2	319	0.900	118	185	137	0.386
58	93.2	94	0.265	119	189.2	1721	4.853
59	94.1	3222	9.086	120	204.3	5067	14.289
60	95.1	2213	6.241	121	204.3	70	0.197
61	96.1	258	0.728	122	205.3	1096	3.091

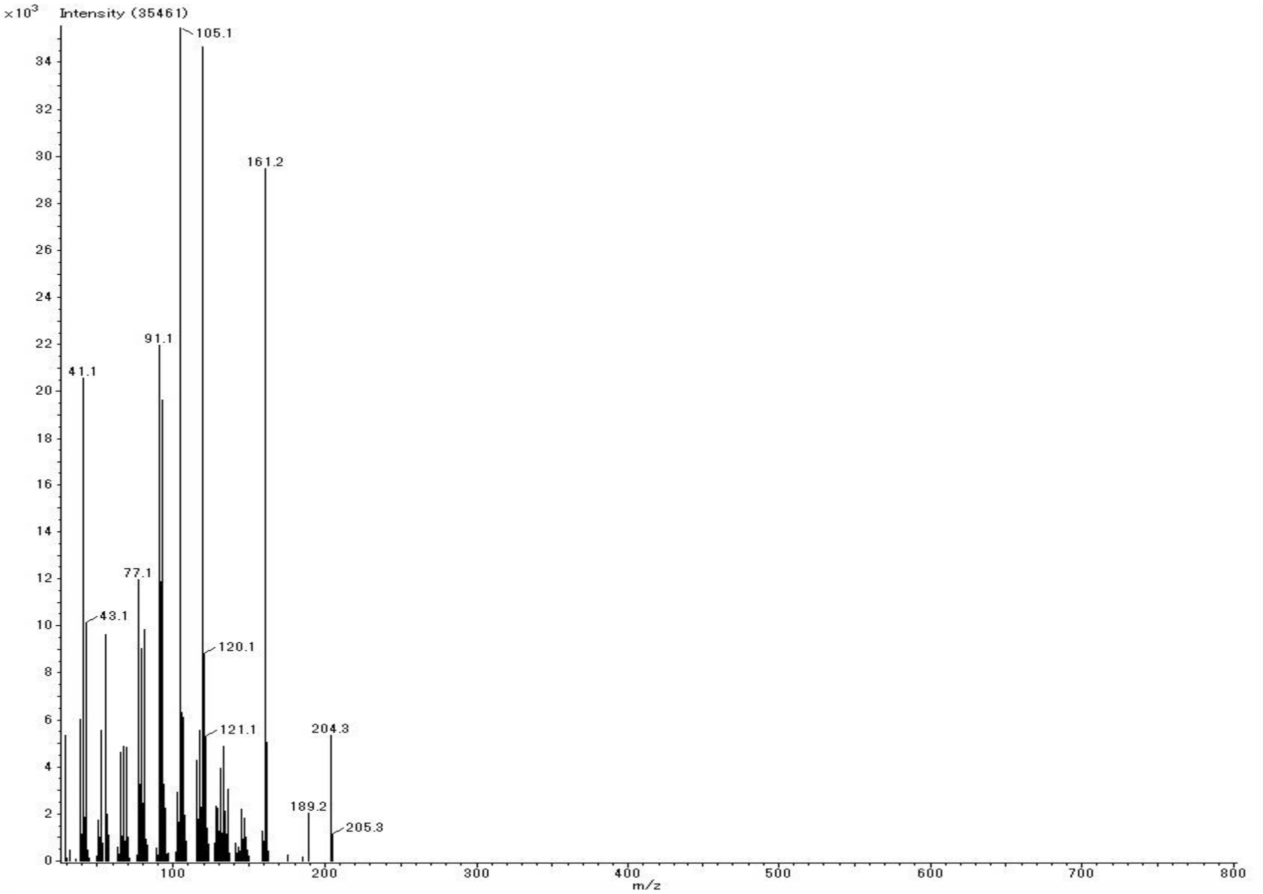

Figure S6a. Mass-spectra of alpha-Copaene (Rt= 10.55) detected in *P. cubeba* essential oil by GC- MS

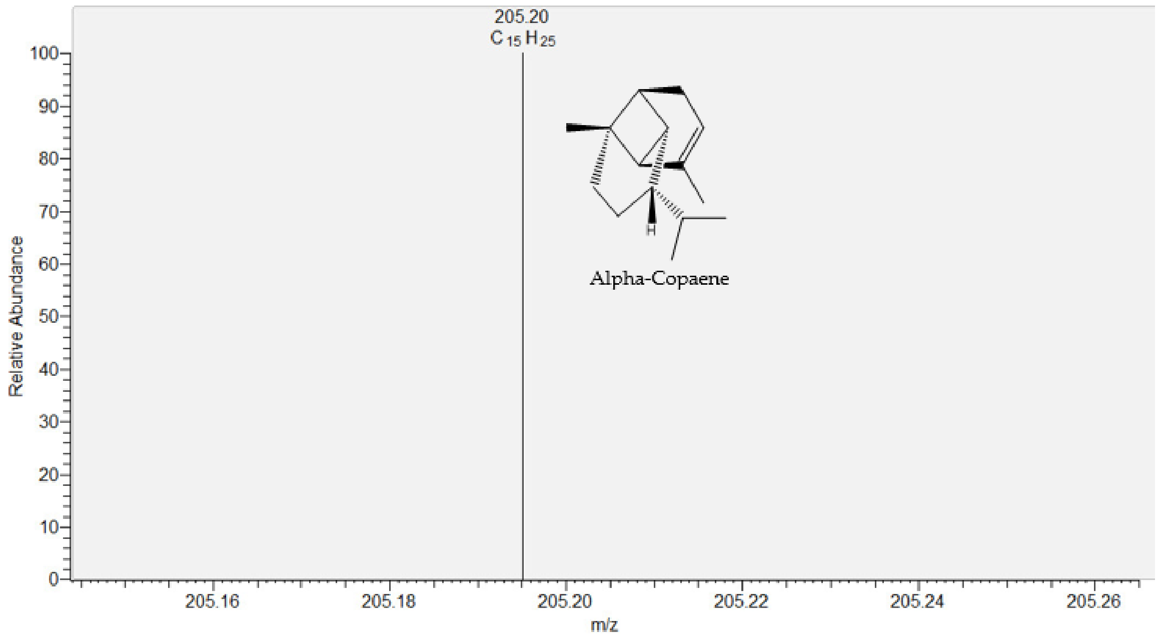

Figure S6b. Spectra of alpha-Copaene in *P. cubeba* essential oil identified by positive ion (H^+) adduction method of LC-ESI-MS

Table S6. Fragmentation pattern of D-Germacrene (retention time = 10.69) in *P. cubeba* essential oil detected by GC-MS

Peak#	m/z	Intensity	Relative Intensity (%)	Peak#	m/z	Intensity	Relative Intensity (%)
1	29	487	12.735	22	81.1	1429	37.369
2	30	450	11.768	23	91.1	2343	61.271
3	32	430	11.245	24	92.1	909	23.771
4	36	534	13.964	25	93.1	1477	38.624
5	38	37	0.968	26	105.1	2352	61.506
6	39	857	22.411	27	106.1	563	14.723
7	41.1	2209	57.767	28	107.1	783	20.476
8	43	250	6.538	29	108.1	325	8.499
9	43.1	1214	31.747	30	115.1	459	12.003
10	44	85	2.223	31	117.1	570	14.906
11	53.1	1102	28.818	32	119.1	1414	36.977
12	54.1	382	9.990	33	120.1	1326	34.676
13	55.1	1646	43.044	34	121.1	569	14.880
14	57.1	402	10.513	35	133.1	804	21.025
15	67.1	1145	29.942	36	147.2	636	16.632
16	68.1	773	20.214	37	161.2	3824	100.000
17	69.1	989	25.863	38	162.2	856	22.385
18	70.1	296	7.741	39	185	483	12.631
19	77.1	1012	26.464	40	204.3	882	23.065
20	78.1	448	11.715	41	212.6	240	6.276
21	79.1	1494	39.069				

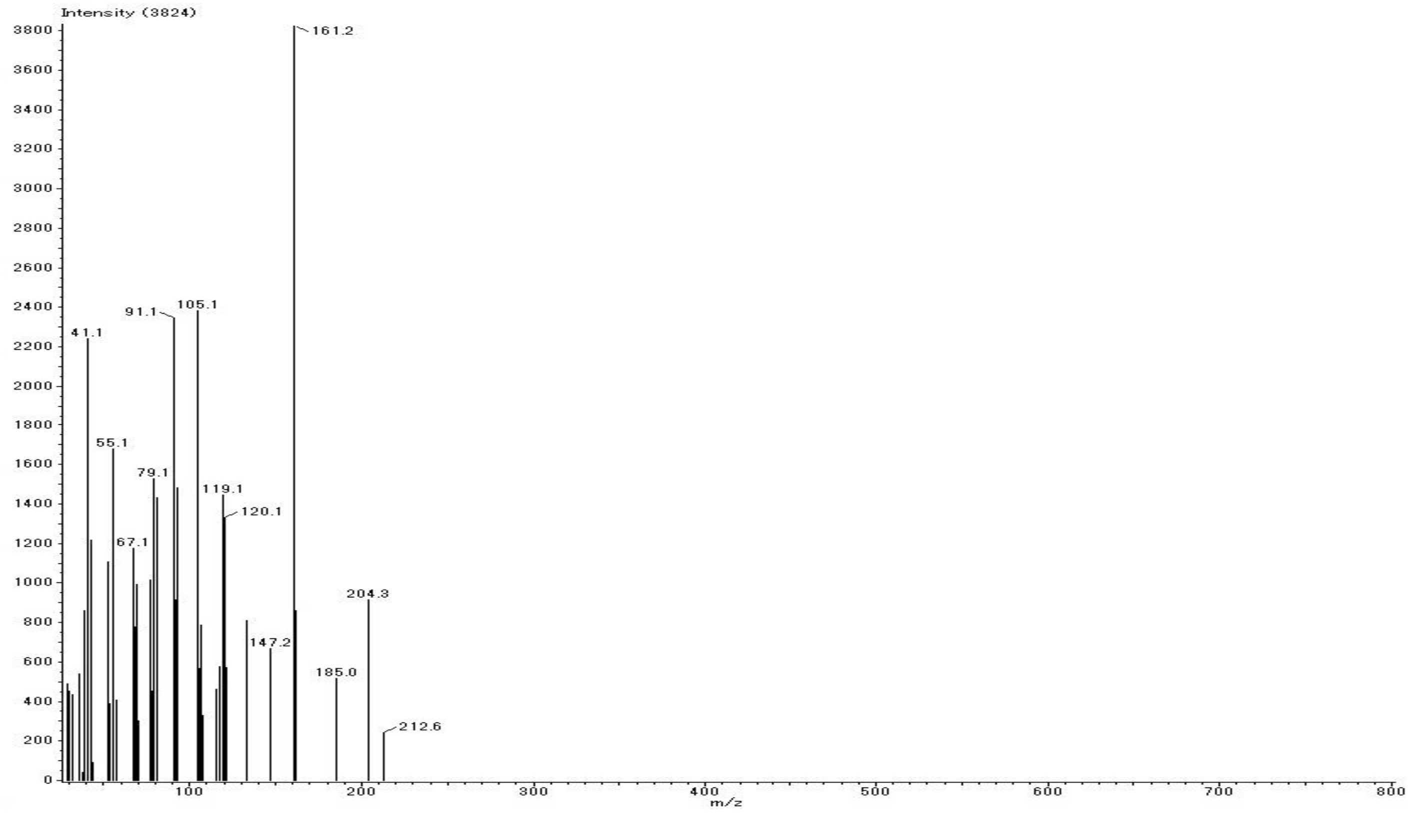

Figure S7a. Mass-spectra of D-Germacrene (Rt= 10.69) detected in *P. cubeba* essential oil by GC- MS

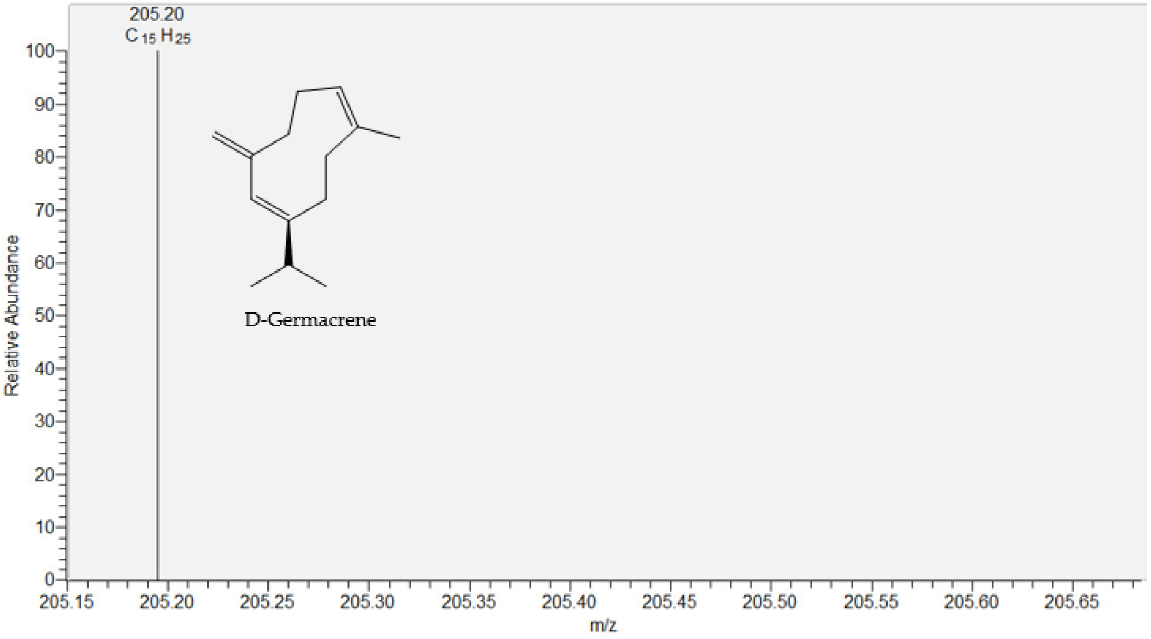

Figure S7b. Spectra of D-Germacrene in *P. cubeba* essential oil identified by positive ion (H^+) adduction method of LC-ESI-MS

Table S7. Fragmentation pattern of γ-Elemene (retention time = 11.21) in *P. cubeba* essential oil detected by GC-MS

Peak#	m/z	Intensity	Relative Intensity (%)	Peak#	m/z	Intensity	Relative Intensity (%)
1	29	183	1.393	40	95.1	1425	10.849
2	29	2475	18.843	41	96.1	234	1.781
3	30	222	1.690	42	103.1	430	3.274
4	32	266	2.025	43	104.1	285	2.170
5	34	127	0.967	44	105.1	5867	44.667
6	38	209	1.591	45	106.1	1560	11.877
7	39	4319	32.882	46	107.1	6280	47.811
8	40	214	1.629	47	108.1	2736	20.830
9	40	1654	12.592	48	109.1	1061	8.078
10	41.1	9330	71.032	49	110	157	1.195
11	42.1	1211	9.220	50	110.1	236	1.797
12	43.1	2763	21.035	51	115.1	776	5.908
13	51	977	7.438	52	116.1	146	1.112
14	52	578	4.400	53	117.1	667	5.078
15	53.1	5788	44.065	54	119.1	4181	31.831
16	54.1	764	5.817	55	120.1	1490	11.344
17	55.1	5345	40.693	56	121.1	13135	100.000
18	56.1	551	4.195	57	121.2	340	2.589
19	57.1	413	3.144	58	122.1	3009	22.908
20	65.1	2189	16.665	59	123.2	835	6.357
21	66.1	657	5.002	60	129.1	236	1.797
22	67.1	7150	54.435	61	131.1	323	2.459
23	68.1	2735	20.822	62	133.1	2937	22.360
24	69.1	2053	15.630	63	134.2	834	6.349
25	70.1	211	1.606	64	135.2	1112	8.466
26	77.1	4900	37.305	65	136.2	2354	17.922
27	77.1	152	1.157	66	137.2	344	2.619
28	78.1	1256	9.562	67	145.1	254	1.934
29	79.1	5994	45.634	68	147.2	1774	13.506
30	80.1	1989	15.143	69	148.2	1129	8.595
31	80.5	188	1.431	70	149.2	532	4.050
32	81.1	4195	31.938	71	161.2	2560	19.490
33	82.1	381	2.901	72	162.2	534	4.065
34	83.1	708	5.390	73	168.9	126	0.959
35	91.1	6387	48.626	74	175.2	230	1.751
36	92.1	1793	13.651	75	187	152	1.157
37	93.1	10329	78.637	76	189.2	1772	13.491
38	93.1	164	1.249	77	190.2	262	1.995
39	94.1	1812	13.795	78	204.3	817	6.220

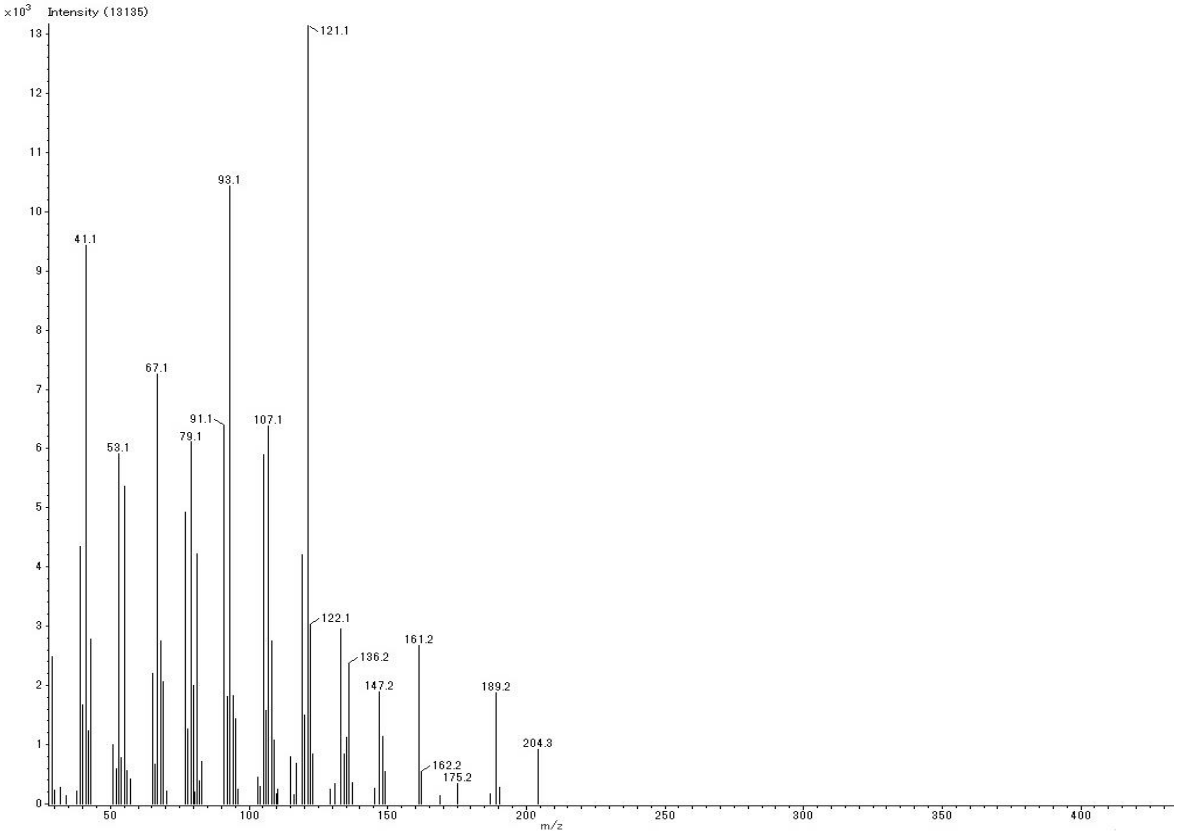

Figure S8a. Mass-spectra of γ-Elemene (Rt= 11.21) detected in *P. cubeba* essential oil by GC- MS

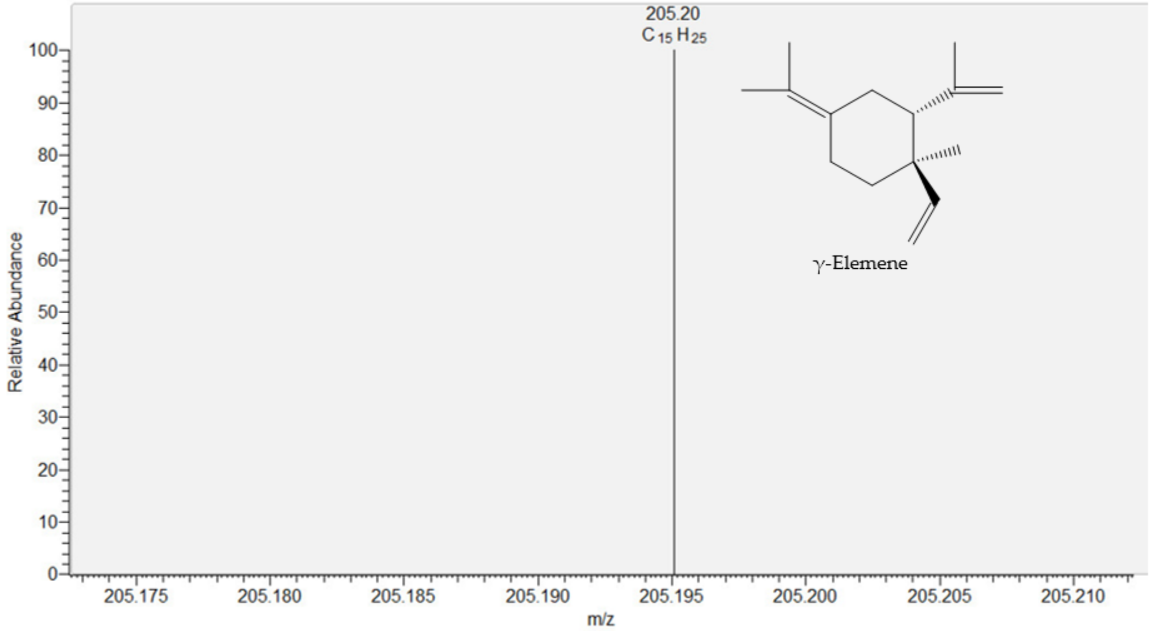

Figure S8b. Spectra of γ-Elemene in *P. cubeba* essential oil identified by positive ion (H^+) adduction method of LC-ESI-MS

Table S8. Fragmentation pattern of δ-Cadinene (retention time = 12.33) in *P. cubeba* essential oil detected by GC-MS

Peak#	m/z	Intensity	Relative Intensity (%)	Peak#	m/z	Intensity	Relative Intensity (%)
1	29	2045	16.171	49	106.1	1547	12.233
2	32	5	0.040	50	107.1	1565	12.375
3	32	654	5.172	51	108.1	220	1.740
4	32.1	105	0.830	52	109.1	407	3.218
5	32.2	65	0.514	53	115.1	2159	17.073
6	36	50	0.395	54	116.1	739	5.844
7	39	2294	18.140	55	117.1	2795	22.102
8	40	197	1.558	56	118.1	846	6.690
9	40	285	2.254	57	119.1	9865	78.009
10	41.1	7396	58.485	58	119.2	301	2.380
11	41.1	161	1.273	59	120.1	1704	13.475
12	42.1	557	4.405	60	121.1	1251	9.892
13	43	1762	13.933	61	122.2	144	1.139
14	43.1	3035	24.000	62	123.1	633	5.006
15	44	111	0.878	63	127.1	241	1.906
16	51	584	4.618	64	128.1	1255	9.924
17	52	330	2.610	65	129.1	1453	11.490
18	53.1	2092	16.543	66	130.1	563	4.452
19	54.1	131	1.036	67	131.1	1664	13.158
20	55.1	3840	30.365	68	132.1	672	5.314
21	56.1	205	1.621	69	133.1	2599	20.552
22	57.1	187	1.479	70	134.2	7750	61.284
23	63	243	1.922	71	135.2	1619	12.802
24	65.1	1686	13.332	72	137.1	124	0.981
25	66.1	283	2.238	73	141.1	560	4.428
26	67.1	1801	14.242	74	142.1	249	1.969
27	68.1	239	1.890	75	143.1	324	2.562
28	69.1	1831	14.479	76	144.1	153	1.210
29	70.1	276	2.183	77	145.1	1348	10.659
30	71.1	428	3.384	78	146.2	749	5.923
31	77.1	3752	29.669	79	147.2	1191	9.418
32	78.1	960	7.591	80	157.2	120	0.949
33	79.1	3495	27.637	81	159.2	971	7.678
34	80.1	506	4.001	82	160.2	675	5.338
35	81.1	3821	30.215	83	161.2	12646	100.000
36	82.1	479	3.788	84	161.3	227	1.795
37	83.1	144	1.139	85	162.2	2999	23.715
38	85	120	0.949	86	163.2	313	2.475
39	89.1	151	1.194	87	175.2	108	0.854
40	91.1	7836	61.964	88	189.2	2125	16.804
41	92.1	2128	16.827	89	190.2	383	3.029
42	93.1	3464	27.392	90	202.2	174	1.376
43	94.1	451	3.566	91	204.3	5808	45.928
44	95.1	1052	8.319	92	204.4	77	0.609
45	102.1	128	1.012	93	205.3	949	7.504
46	103.1	1060	8.382	94	206.3	113	0.894
47	104.1	523	4.136	95	207.2	1353	10.699
48	105.1	8882	70.236				

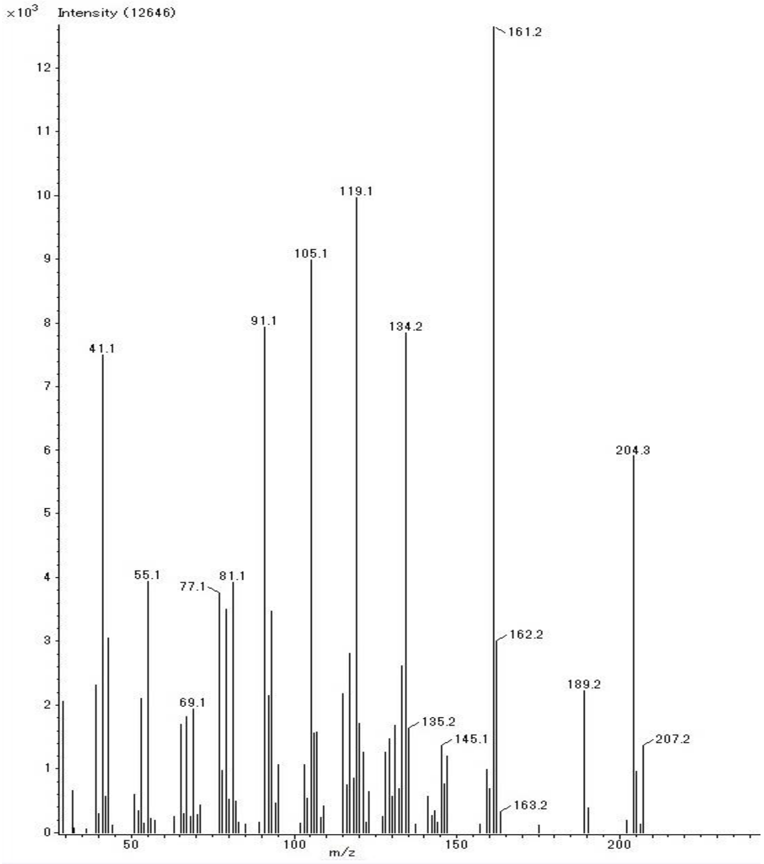

Figure S9a. Mass-spectra of δ-Cadinene (Rt= 12.33) detected in *P. cubeba* essential oil by GC- MS

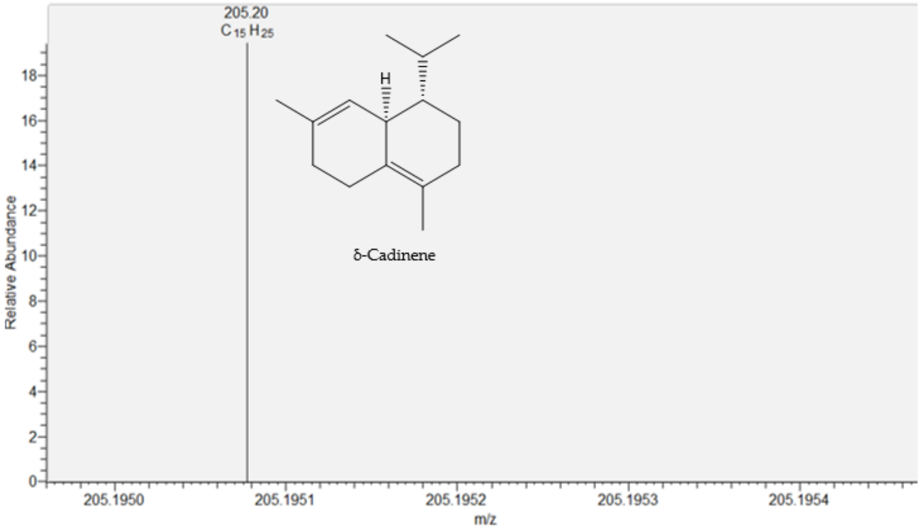

Figure S9b. Spectra of δ-Cadinene in *P. cubeba* essential oil identified by positive ion (H^+) adduction method of LC-ESI-MS

Table S9. Fragmentation pattern of α-Elemol (retention time = 12.68) in *P. cubeba* essential oil detected by GC-MS

Peak#	m/z	Intensity	Relative Intensity (%)	Peak#	m/z	Intensity	Relative Intensity (%)
1	29	36	0.255	41	84.1	591	4.185
2	29	1972	13.964	42	85.1	279	1.976
3	31	2099	14.863	43	91.1	3550	25.138
4	32	386	2.733	44	92.1	1507	10.671
5	34	193	1.367	45	93.1	7828	55.431
6	36	184	1.303	46	94.1	2075	14.693
7	38	200	1.416	47	95.1	2694	19.077
8	39	2303	16.308	48	96.1	922	6.529
9	40	880	6.231	49	103.7	160	1.133
10	41.1	6743	47.748	50	105.1	2767	19.594
11	42.1	761	5.389	51	106.1	771	5.460
12	43	5468	38.720	52	107.1	4748	33.621
13	43.1	2269	16.067	53	108.1	2027	14.353
14	45	564	3.994	54	109.1	1915	13.560
15	51	207	1.466	55	115.1	146	1.034
16	53.1	3264	23.113	56	117.1	248	1.756
17	54.1	335	2.372	57	119.1	2129	15.076
18	55.1	3942	27.914	58	120.1	665	4.709
19	56.1	569	4.029	59	121.1	3706	26.243
20	57.1	776	5.495	60	122.1	1025	7.258
21	59.1	14122	100.000	61	123.2	1124	7.959
22	59.1	247	1.749	62	131.1	474	3.356
23	60.1	651	4.610	63	133.1	1483	10.501
24	65.1	1444	10.225	64	134.2	959	6.791
25	66.1	590	4.178	65	135.2	2021	14.311
26	67.1	5475	38.769	66	136.2	1154	8.172
27	68.1	3636	25.747	67	137.1	149	1.055
28	69.1	2855	20.217	68	147.2	1238	8.766
29	70.1	271	1.919	69	148.2	717	5.077
30	71.1	2602	18.425	70	149.2	1270	8.993
31	71.1	124	0.878	71	161.2	3646	25.818
32	72.1	273	1.933	72	162.2	1160	8.214
33	77.1	2660	18.836	73	163.2	1026	7.265
34	78.1	616	4.362	74	164.2	186	1.317
35	79.1	5220	36.964	75	175.2	286	2.025
36	80.1	1879	13.305	76	185	496	3.512
37	81.1	4795	33.954	77	189.2	1444	10.225
38	82.1	1242	8.795	78	204.3	909	6.437
39	83	52	0.368	79	590.4	156	1.105
40	83.1	775	5.488				

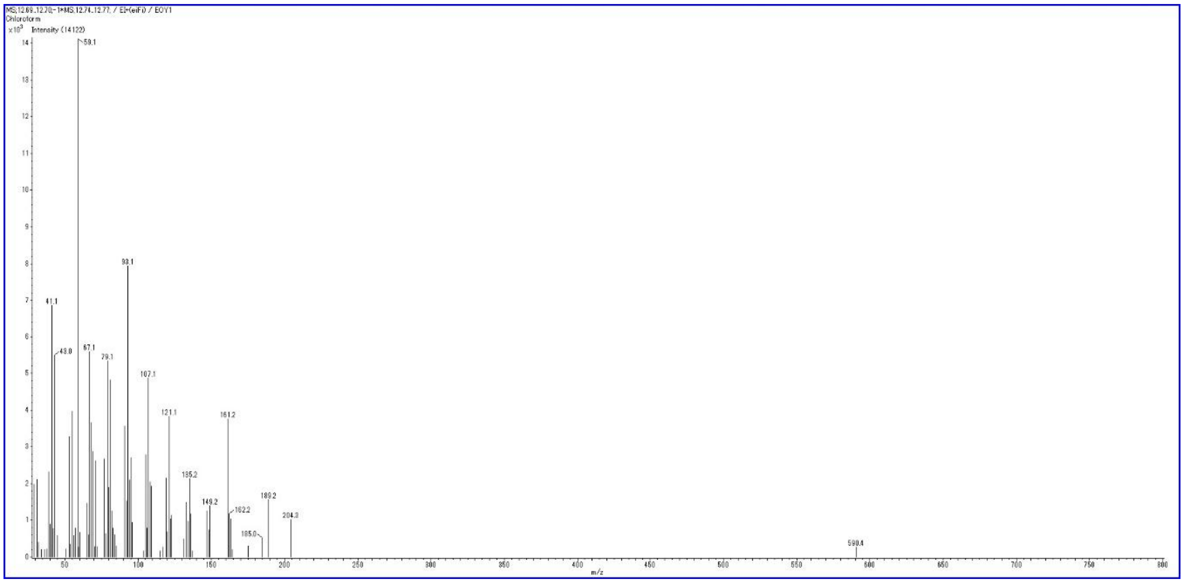

Figure S10a. Mass-spectra of α-Elemol (Rt= 12.68) detected in *P. cubeba* essential oil by GC- MS

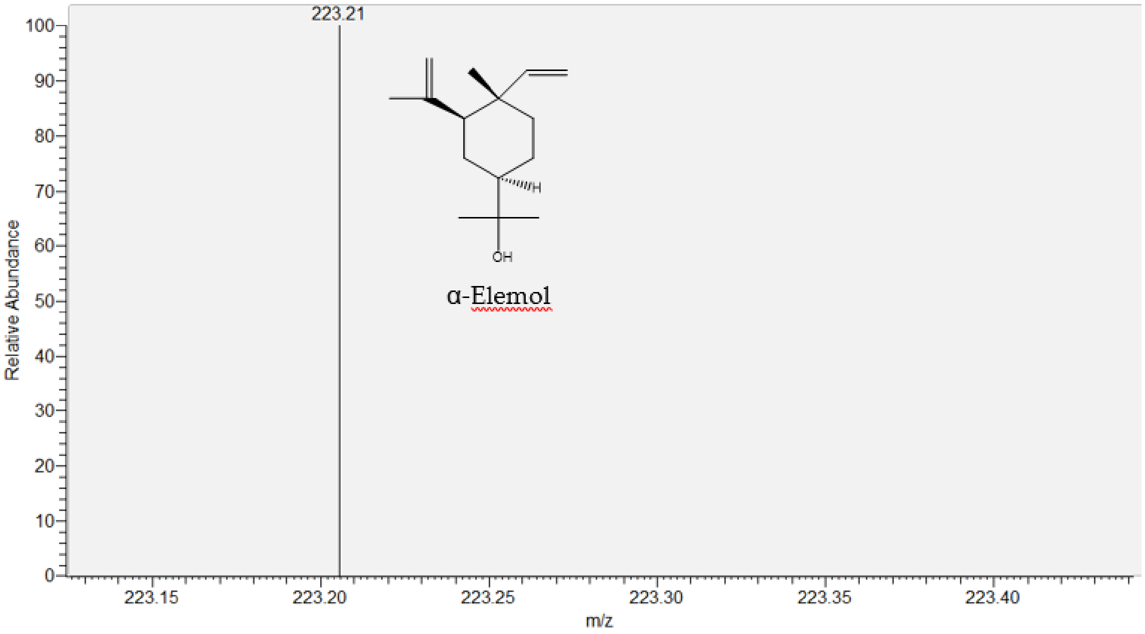

Figure S10b. Spectra of α-Elemol in *P. cubeba* essential oil identified by positive ion (H^+) adduction method of LC-ESI-MS

Table S10. Fragmentation pattern of Spathulenol (retention time = 13.07) in *P. cubeba* essential oil detected by GC-MS

Peak#	m/z	Intensity	Relative Intensity (%)	Peak#	m/z	Intensity	Relative Intensity (%)
1	29	7	0.205	33	92.1	522	15.312
2	29	939	27.545	34	93.1	1546	45.351
3	29.1	102	2.992	35	94.1	573	16.808
4	32	228	6.688	36	95.1	673	19.742
5	32.1	251	7.363	37	103.1	204	5.984
6	34	208	6.101	38	105.1	1626	47.697
7	36	5	0.147	39	106.1	651	19.097
8	38	254	7.451	40	107.1	1181	34.644
9	39	1093	32.062	41	108.1	209	6.131
10	41.1	2786	81.725	42	109.1	218	6.395
11	42.1	238	6.982	43	115.1	323	9.475
12	43	3409	100.000	44	117.1	548	16.075
13	43.1	1103	32.356	45	119.1	1702	49.927
14	51	220	6.454	46	120.1	359	10.531
15	52	188	5.515	47	121.1	529	15.518
16	53.1	1117	32.766	48	123.1	171	5.016
17	55.1	1124	32.972	49	131.1	1075	31.534
18	58.1	522	15.312	50	133.1	478	14.022
19	65.1	363	10.648	51	134.2	608	17.835
20	66.1	181	5.309	52	135.1	155	4.547
21	67.1	1319	38.692	53	145.2	498	14.608
22	68.1	170	4.987	54	146.2	325	9.534
23	69.1	1117	32.766	55	147.2	991	29.070
24	71.1	561	16.456	56	149.2	242	7.099
25	77.1	1483	43.502	57	159.2	1114	32.678
26	78.1	247	7.246	58	160.2	404	11.851
27	79.1	1864	54.679	59	162.2	853	25.022
28	80.1	306	8.976	60	185	43	1.261
29	81.1	694	20.358	61	187.2	485	14.227
30	82.1	458	13.435	62	202.2	370	10.854
31	83.1	228	6.688	63	205.2	1462	42.886
32	91.1	2448	71.810				

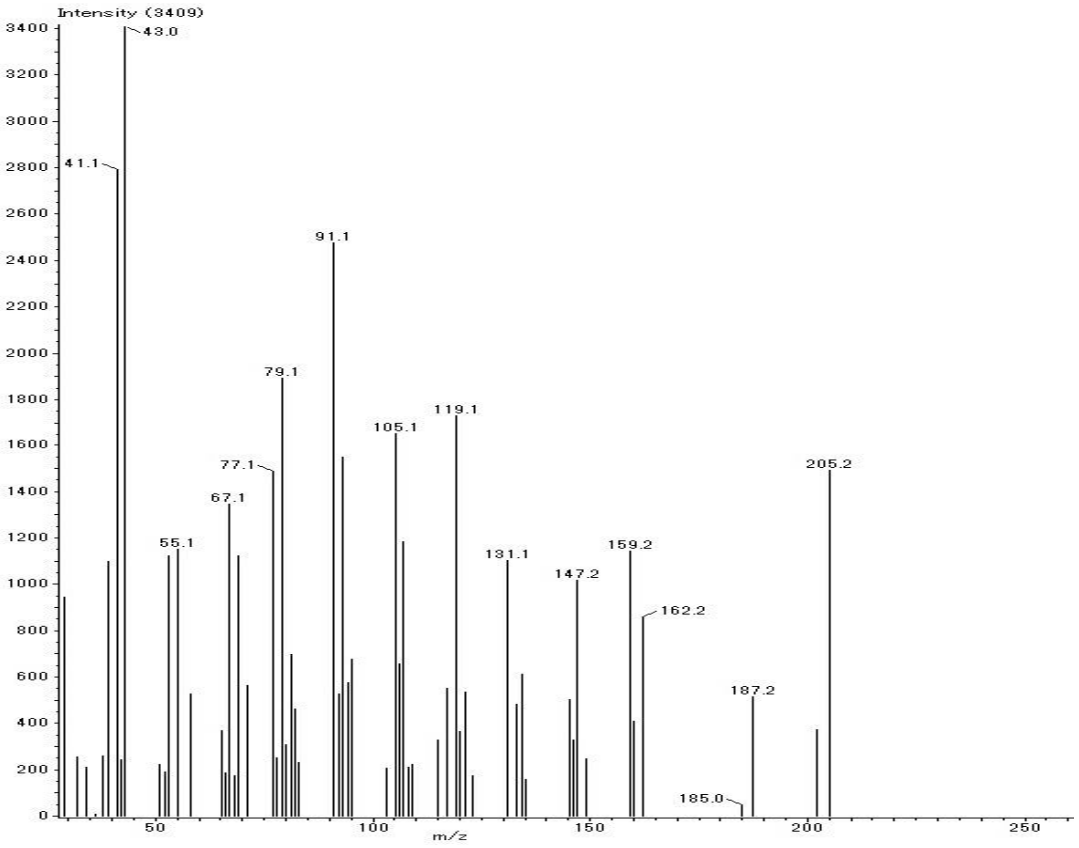

Figure S11a. Mass-spectra of Spathulenol (Rt= 13.07) detected in *P. cubeba* essential oil by GC- MS

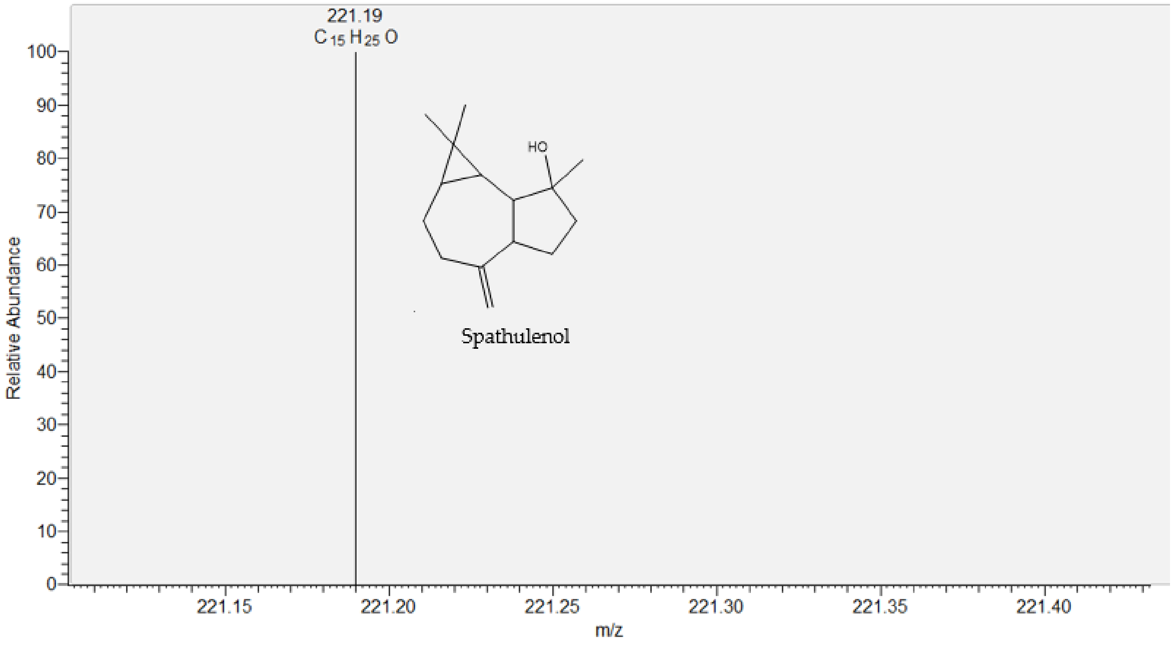

Figure S11b. Spectra of Spathulenol in *P. cubeba* essential oil identified by positive ion (H^+) adduction method of LC-ESI-MS

Table S11. Fragmentation pattern of Cubenol (retention time = 13.65) in *P. cubeba* essential oil detected by GC-MS

Peak#	m/z	Intensity	Relative Intensity (%)	Peak#	m/z	Intensity	Relative Intensity (%)
1	29	102	1.901565996	56	95.1	815	15.193885
2	29	1144	21.32736764	57	95.1	1098	20.469799
3	29.3	35	0.652498136	58	97.1	187	3.4862043
4	29.7	45	0.838926174	59	103.1	325	6.0589113
5	32	117	2.181208054	60	104.1	55	1.0253542
6	32.3	97	1.808351976	61	105.1	3049	56.841909
7	34	51	0.950782998	62	106.1	1002	18.680089
8	35	53	0.988068606	63	107.1	1753	32.680835
9	36	100	1.864280388	64	108.1	174	3.2438479
10	37.9	76	1.416853095	65	109.1	640	11.931394
11	38	60	1.118568233	66	109.1	606	11.297539
12	39	1353	25.22371365	67	110.1	538	10.029828
13	40	133	2.479492916	68	111.1	411	7.6621924
14	40	98	1.82699478	69	115.1	530	9.8806861
15	41.1	4414	82.28933632	70	116.1	109	2.0320656
16	42.1	232	4.3251305	71	117.1	1075	20.041014
17	43	3698	68.94108874	72	118.1	313	5.8351976
18	43.1	1951	36.37211037	73	119.1	5364	100
19	44	16	0.298284862	74	120.1	1068	19.910515
20	45	97	1.808351976	75	121.1	40	0.7457122
21	49.8	56	1.043997017	76	121.1	1176	21.923937
22	51	244	4.548844146	77	122.1	572	10.663684
23	53.1	1347	25.11185682	78	123.1	429	7.9977629
24	54.1	82	1.528709918	79	123.2	215	4.0082028
25	55	290	5.406413125	80	128.1	78	1.4541387
26	55.1	2124	39.59731544	81	129.1	380	7.0842655
27	56.1	257	4.791200597	82	131.1	872	16.256525
28	57.1	75	1.398210291	83	133.1	904	16.853095
29	57.1	446	8.314690529	84	134.2	621	11.577181
30	58.1	588	10.96196868	85	135.2	548	10.216257
31	59.1	145	2.703206562	86	136.1	101	1.8829232
32	65.1	909	16.94630872	87	137.1	190	3.5421327
33	66.1	285	5.313199105	88	143.1	91	1.6964952
34	67.1	1623	30.25727069	89	145.1	559	10.421327
35	68.1	141	2.628635347	90	147.2	1228	22.893363
36	69.1	209	3.89634601	91	148.2	106	1.9761372
37	69.1	1191	22.20357942	92	149.2	406	7.5689784
38	70.1	64	1.193139448	93	153.2	39	0.7270694
39	71.1	781	14.56002983	94	159.2	1480	27.59135
40	72.1	49	0.91349739	95	160.2	272	5.0708427
41	77.1	2159	40.24981357	96	161.2	2553	47.595078
42	78.1	612	11.40939597	97	162.2	1331	24.813572
43	79.1	2395	44.64951529	98	163.2	77	1.4354959
44	80.1	472	8.79940343	99	177.2	367	6.841909
45	81.1	1744	32.51304996	100	179.2	798	14.876957
46	82.1	1108	20.6562267	101	187	139	2.5913497
47	83	115	2.143922446	102	187.2	426	7.9418345
48	83.1	183	3.41163311	103	189.2	137	2.5540641
49	83.1	429	7.997762864	104	202.2	314	5.8538404
50	85	49	0.91349739	105	204.3	1104	20.581655
51	85.1	144	2.684563758	106	205.2	635	11.83818
52	91.1	3394	63.27367636	107	220.2	58	1.0812826
53	92.1	883	16.46159582	108	243.3	56	1.043997
54	93.1	2768	51.60328113	109	494.2	97	1.808352
55	94.1	491	9.153616704				

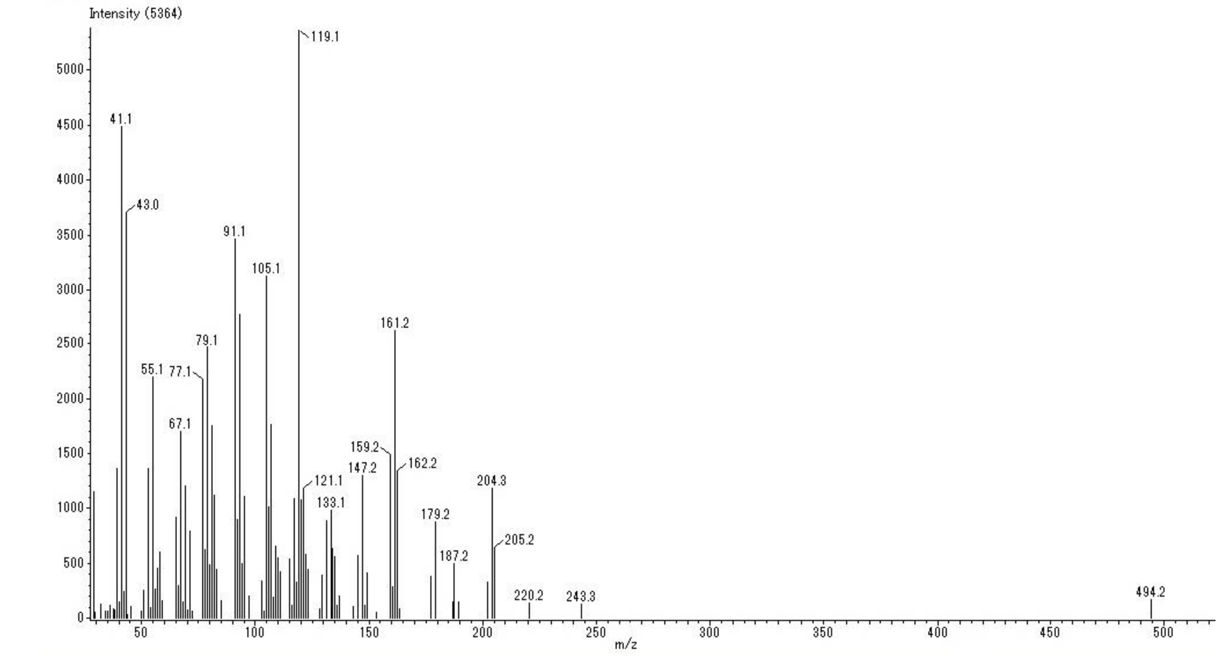

Figure S12a. Mass-spectra of Cubenol (Rt= 13.65) detected in *P. cubeba* essential oil by GC- MS

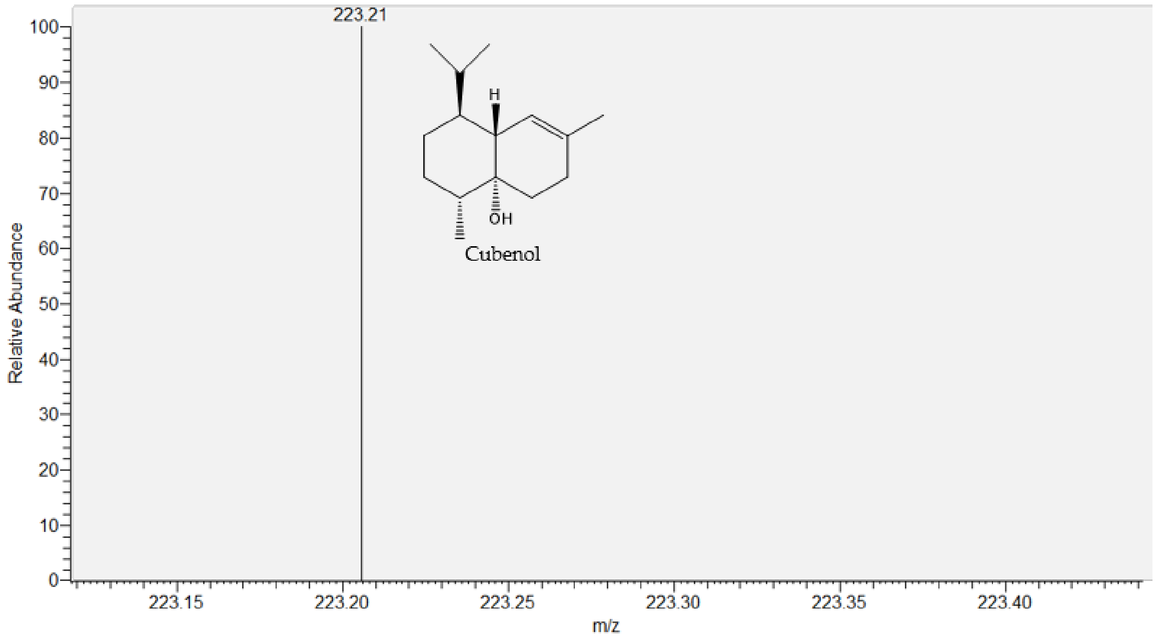

Figure S12b. Spectra of Cubenol in *P. cubeba* essential oil identified by positive ion (H^+) adduction method of LC-ESI-MS

Table S12. Fragmentation pattern of β-Eudesmol (retention time = 13.65) in *P. cubeba* essential oil detected by GC-MS

Peak#	m/z	Intensity	Relative Intensity (%)	Peak#	m/z	Intensity	Relative Intensity (%)
1	29	748	11.917	41	93.1	2082	33.169
2	29.1	145	2.310	42	94.1	868	13.828
3	29.3	88	1.402	43	95.1	2120	33.774
4	31	682	10.865	44	96.1	350	5.576
5	32	394	6.277	45	105.1	1545	24.614
6	32.1	295	4.700	46	106.1	453	7.217
7	32.3	50	0.797	47	107.1	1474	23.483
8	35	74	1.179	48	108.1	1434	22.845
9	39	1104	17.588	49	109.1	1642	26.159
10	40	246	3.919	50	110.1	122	1.944
11	41.1	2502	39.860	51	115.1	348	5.544
12	42.1	155	2.469	52	117.1	353	5.624
13	43	3189	50.805	53	119.1	827	13.175
14	43.1	1257	20.025	54	120.1	106	1.689
15	44	32	0.510	55	121.1	1799	28.660
16	45.1	274	4.365	56	122.1	1155	18.401
17	53.1	1195	19.038	57	123.2	869	13.844
18	55.1	1969	31.368	58	131.1	201	3.202
19	56.1	135	2.151	59	133.1	678	10.801
20	57.1	240	3.823	60	134.2	140	2.230
21	58.1	422	6.723	61	135.2	758	12.076
22	59.1	6277	100.000	62	145.2	271	4.317
23	59.1	143	2.278	63	147.2	414	6.596
24	60.1	116	1.848	64	148.2	79	1.259
25	65.1	727	11.582	65	149.2	2367	37.709
26	66.1	109	1.736	66	150.2	280	4.461
27	67.1	1472	23.451	67	157.2	333	5.305
28	68.1	653	10.403	68	159.2	158	2.517
29	69.1	227	3.616	69	161.2	1321	21.045
30	69.1	973	15.501	70	162.2	84	1.338
31	71.1	985	15.692	71	163.2	140	2.230
32	77.1	1147	18.273	72	164.2	957	15.246
33	78.1	330	5.257	73	175.2	89	1.418
34	79.1	2235	35.606	74	187	87	1.386
35	80.1	792	12.617	75	189.2	737	11.741
36	81.1	2060	32.818	76	190.2	76	1.211
37	82.1	823	13.111	77	202.2	562	8.953
38	83.1	373	5.942	78	204.3	1017	16.202
39	91.1	1968	31.353	79	426.1	113	1.800
40	92.1	472	7.520	80	535.5	107	1.705

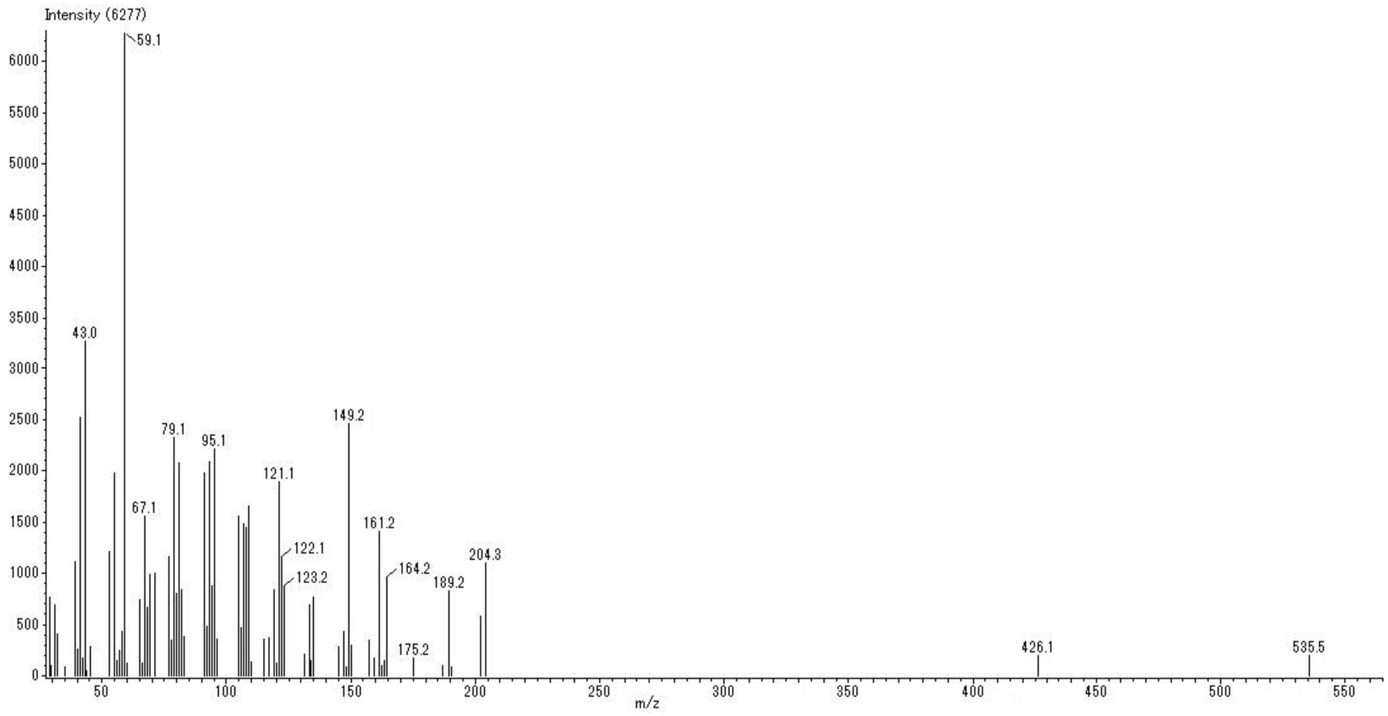

Figure S13a. Mass-spectra of β-Eudesmol (Rt= 14.00) detected in *P. cubeba* essential oil by GC- MS

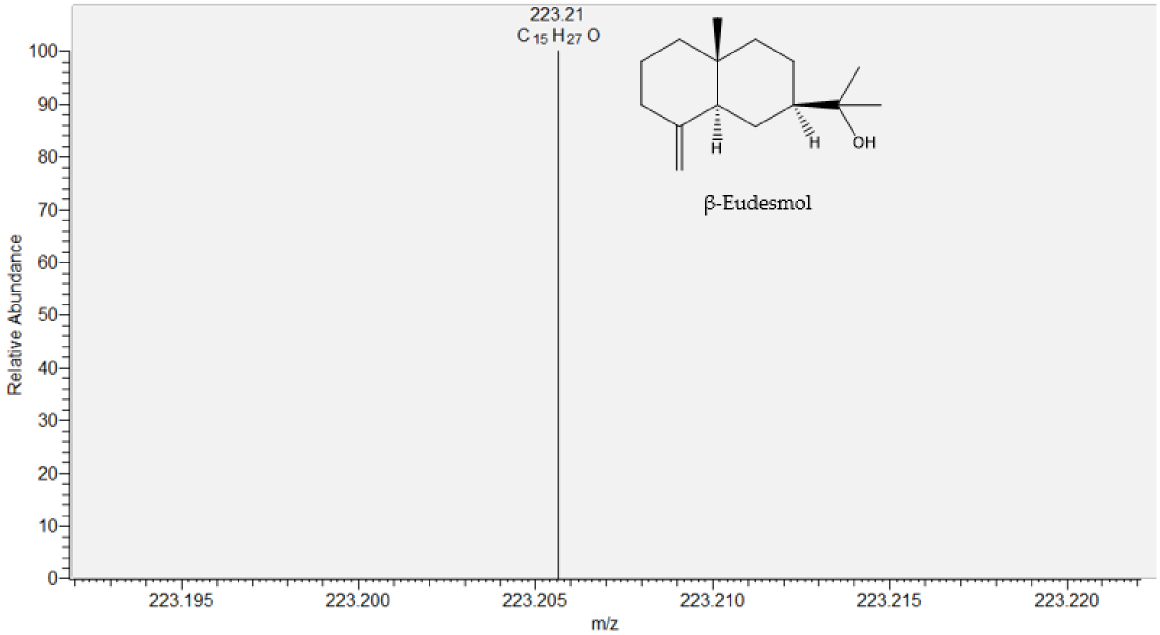

Figure S13b. Spectra of β-Eudesmol in *P. cubeba* essential oil identified by positive ion (H^+) adduction method of LC-ESI-MS

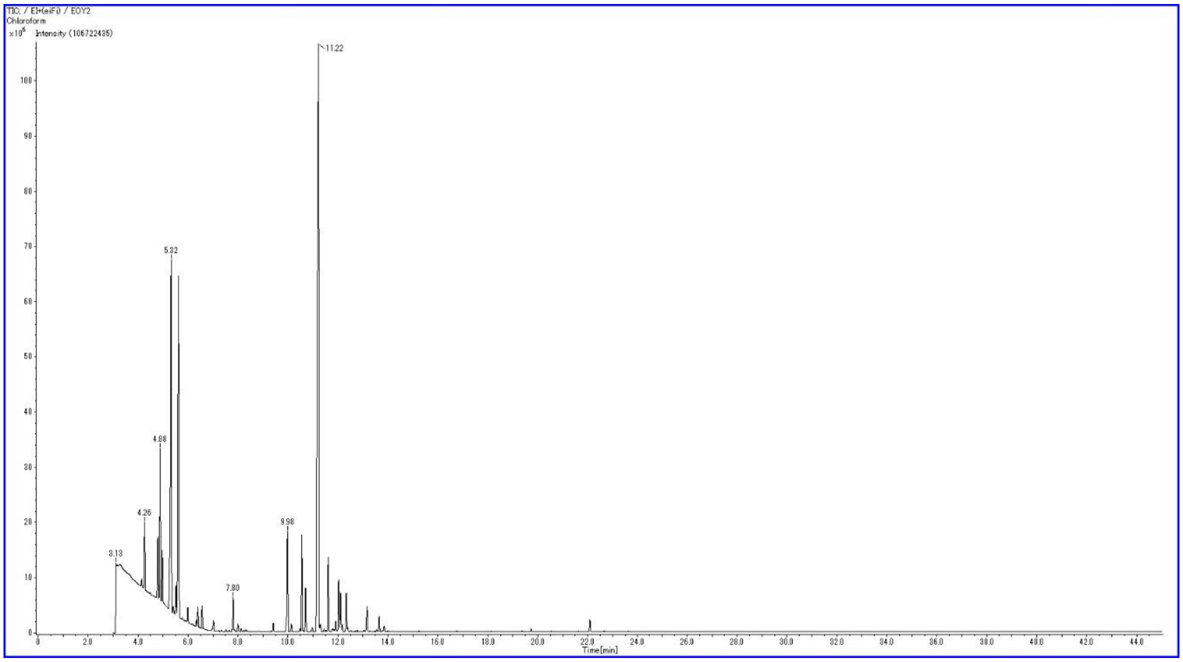

Figure S14. GC-MS Chromatogram of *Piper nigrum* essential oil

Table S13. Fragmentation pattern of β-Thujene (retention time = 13.65) in *P. nigrum* essential oil detected by GC-MS

Peak#	m/z	Intensity	Relative Intensity (%)	Peak#	m/z	Intensity	Relative Intensity
1	29	31831	4.259	55	41.6	153	0.020
2	29.2	31	0.004	56	41.6	148	0.020
3	29.3	1005	0.134	57	41.8	106	0.014
4	29.4	297	0.040	58	42.1	9304	1.245
5	29.6	85	0.011	59	42.1	473	0.063
6	29.7	1441	0.193	60	43.1	57697	7.721
7	29.9	30	0.004	61	43.1	1118	0.150
8	29.9	591	0.079	62	43.4	104	0.014
9	30.1	600	0.080	63	43.4	70	0.009
10	30.3	95	0.013	64	44.1	2295	0.307
11	32.1	982	0.131	65	45	206	0.028
12	32.1	530	0.071	66	47	128	0.017
13	32.3	153	0.020	67	50	11510	1.540
14	32.5	3063	0.410	68	50.1	36	0.005
15	32.6	111	0.015	69	51	53407	7.146
16	32.6	143	0.019	70	51.1	1167	0.156
17	32.7	317	0.042	71	51.1	664	0.089
18	33.1	323	0.043	72	51.2	58	0.008
19	33.2	340	0.045	73	51.4	120	0.016
20	33.2	126	0.017	74	51.5	340	0.045
21	33.4	310	0.041	75	52	22542	3.016
22	33.6	81	0.011	76	52.1	361	0.048
23	35	90	0.012	77	52.6	610	0.082
24	36	2	0.000	78	53.1	48462	6.485
25	37	1098	0.147	79	53.1	998	0.134
26	38	66	0.009	80	53.2	602	0.081
27	38	5997	0.802	81	53.4	108	0.014
28	39	125435	16.785	82	54.1	5427	0.726
29	39.2	706	0.094	83	55.1	19515	2.611
30	39.3	465	0.062	84	55.1	151	0.020
31	39.3	1308	0.175	85	55.2	27	0.004
32	39.4	1085	0.145	86	56.1	1702	0.228
33	39.5	96	0.013	87	57	1015	0.136
34	39.6	390	0.052	88	57.1	890	0.119
35	39.6	373	0.050	89	57.5	2147	0.287
36	39.8	152	0.020	90	58.1	2609	0.349
37	39.8	109	0.015	91	58.6	1448	0.194
38	40	18548	2.482	92	59.1	1290	0.173
39	40.1	777	0.104	93	59.6	545	0.073
40	40.2	66	0.009	94	60.6	170	0.023
41	40.3	223	0.030	95	61	373	0.050
42	40.3	55	0.007	96	62	4212	0.564
43	40.3	242	0.032	97	63	20913	2.798
44	40.5	49	0.007	98	63.1	529	0.071
45	40.6	55	0.007	99	64.1	5864	0.785
46	40.7	53	0.007	100	65.1	70846	9.480
47	41.1	127153	17.015	101	65.1	1209	0.162
48	41.2	1026	0.137	102	65.4	124	0.017
49	41.3	229	0.031	103	65.5	70	0.009
50	41.3	1144	0.153	104	66.1	11975	1.602
51	41.4	593	0.079	105	67.1	20773	2.780
52	41.4	69	0.009	106	67.1	213	0.029
53	41.4	52	0.007	107	67.2	118	0.016
54	41.6	249	0.033	108	67.2	94	0.013

Table S13. Cont.

Peak#	m/z	Intensity	Relative Intensity (%)	Peak#	m/z	Intensity	Relative Intensity (%)
109	68.1	4886	0.654	163	83	53	0.007
110	68.1	82	0.011	164	83.1	135	0.018
111	69.1	2315	0.310	165	86	526	0.070
112	70.1	193	0.026	166	87.1	900	0.120
113	74	1873	0.251	167	88.1	258	0.035
114	75	2530	0.339	168	89.1	9279	1.242
115	76.1	2947	0.394	169	90.1	2939	0.393
116	77.1	324186	43.380	170	91.1	458236	61.317
117	77.2	739	0.099	171	91.4	8595	1.150
118	77.3	433	0.058	172	91.4	614	0.082
119	77.4	605	0.081	173	91.5	1635	0.219
120	77.5	2472	0.331	174	91.6	1005	0.134
121	77.5	1767	0.236	175	91.8	54	0.007
122	77.6	85	0.011	176	91.9	3234	0.433
123	77.8	2732	0.366	177	92	187	0.025
124	77.8	886	0.119	178	92.1	210204	28.128
125	77.9	120	0.016	179	92.3	615	0.082
126	78.1	58886	7.880	180	92.5	208	0.028
127	78.2	146	0.020	181	92.6	2606	0.349
128	78.4	141	0.019	182	92.6	60	0.008
129	78.5	903	0.121	183	92.9	259	0.035
130	78.5	618	0.083	184	92.9	338	0.045
131	78.6	55	0.007	185	93.1	747318	100.000
132	78.8	156	0.021	186	93.4	613	0.082
133	78.8	1071	0.143	187	93.4	416	0.056
134	78.9	374	0.050	188	93.4	1198	0.160
135	79.1	92936	12.436	189	93.5	4884	0.654
136	79.2	1169	0.156	190	93.6	3826	0.512
137	79.4	136	0.018	191	93.9	9896	1.324
138	79.5	933	0.125	192	94	123	0.016
139	79.5	621	0.083	193	94.1	60533	8.100
140	79.6	382	0.051	194	94.2	2667	0.357
141	79.8	247	0.033	195	94.3	723	0.097
142	79.8	553	0.074	196	94.6	261	0.035
143	79.9	586	0.078	197	94.7	3467	0.464
144	79.9	96	0.013	198	94.7	56	0.007
145	80.1	38054	5.092	199	94.9	68	0.009
146	80.2	1177	0.157	200	95	224	0.030
147	80.3	172	0.023	201	95	590	0.079
148	80.5	106	0.014	202	95.1	559	0.075
149	80.5	236	0.032	203	95.1	4254	0.569
150	80.6	125	0.017	204	95.3	40	0.005
151	80.8	53	0.007	205	95.4	481	0.064
152	80.8	22	0.003	206	95.4	415	0.056
153	80.8	76	0.010	207	95.5	108	0.014
154	80.9	130	0.017	208	95.8	326	0.044
155	80.9	57	0.008	209	95.8	36	0.005
156	81.1	9367	1.253	210	96.1	588	0.079
157	81.2	200	0.027	211	96.2	89	0.012
158	81.2	86	0.012	212	98.1	120	0.016
159	81.5	20	0.003	213	101.1	387	0.052
160	81.5	21	0.003	214	102.1	2867	0.384
161	81.6	97	0.013	215	103.1	13430	1.797
162	82.1	1088	0.146	216	103.1	163	0.022

Table S13. Cont.

Peak#	m/z	Intensity	Relative Intensity (%)
217	103.2	79	0.011
218	103.4	46	0.006
219	104.1	5545	0.742
220	105.1	36679	4.908
221	105.2	363	0.049
222	105.2	281	0.038
223	106.1	8550	1.144
224	106.2	77	0.010
225	107.1	13441	1.799
226	107.2	201	0.027
227	108.1	2961	0.396
228	109.1	433	0.058
229	115.1	7766	1.039
230	116.1	1948	0.261
231	117.1	5943	0.795
232	118.1	1270	0.170
233	119.1	15235	2.039
234	119.2	245	0.033
235	119.2	58	0.008
236	120.1	2753	0.368
237	121.1	33964	4.545
238	121.2	135	0.018
239	121.6	83	0.011
240	121.6	115	0.015
241	121.7	78	0.010
242	122.1	4131	0.553
243	123.1	199	0.027
244	128.1	414	0.055
245	132.1	128	0.017
246	133.1	290	0.039
247	134.2	2330	0.312
248	135.2	670	0.090
249	136.2	117870	15.772
250	136.3	463	0.062
251	136.4	577	0.077
252	136.6	243	0.033
253	136.7	965	0.129
254	136.8	765	0.102
255	137.1	52	0.007
256	137.2	13980	1.871
257	137.3	216	0.029
258	137.7	73	0.010
259	138.2	906	0.121
260	185	341	0.046
261	187	207	0.028
262	219	79	0.011
263	233	176	0.024
264	235	176	0.024
265	250	87	0.012
266	252	856	0.115
267	662.5	64	0.009

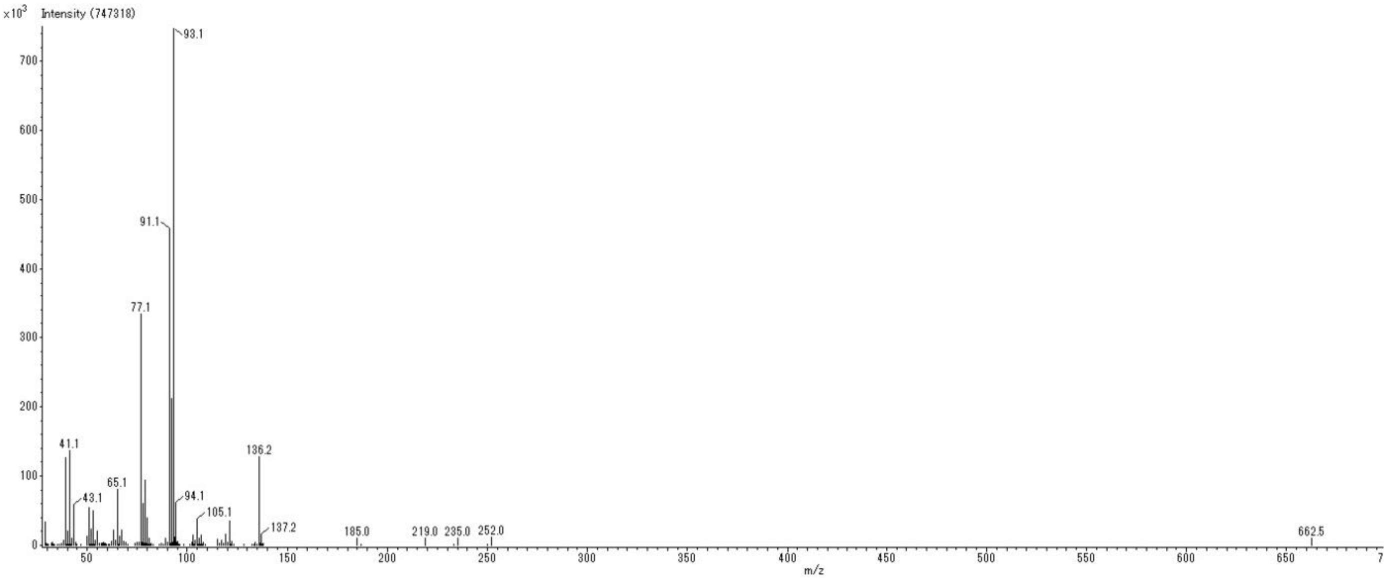

Figure S15a. Mass-spectra of β-Thujene (Rt= 5.32) detected in *P. nigrum* essential oil by GC- MS

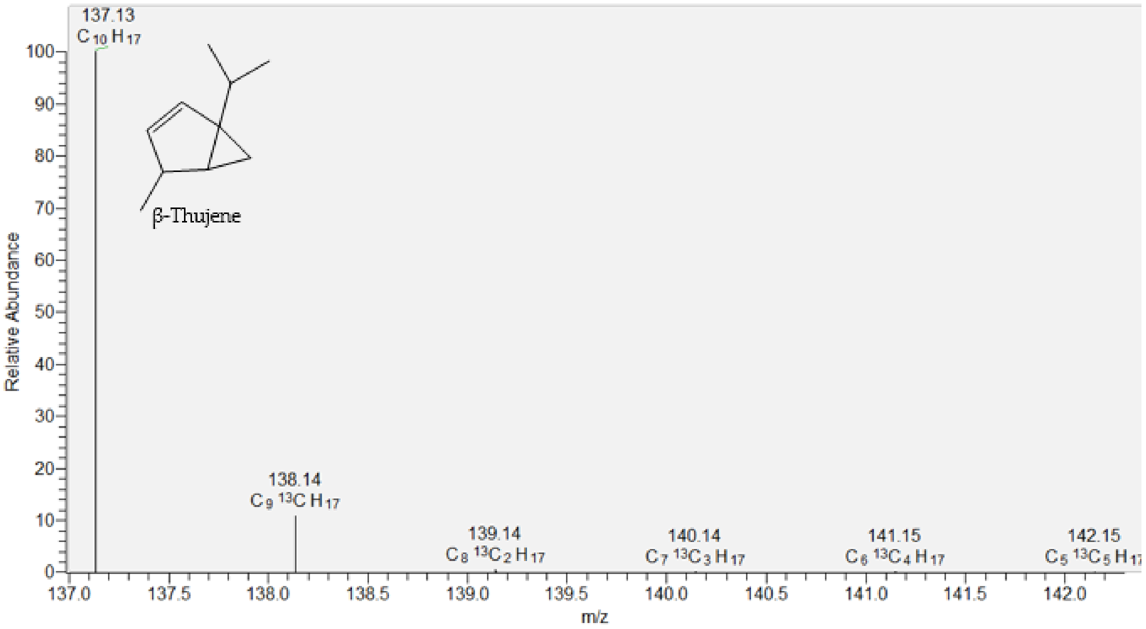

Figure S15b. Spectra of β-Thujene in *P. nigrum* essential oil identified by positive ion (H^+) adduction method of LC-ESI-MS

Table S14. Fragmentation pattern of Terpinen-4-ol (retention time = 7.81) in *P. nigrum* essential oil detected by GC-MS

Peak#	m/z	Intensity	Relative Intensity (%)	Peak#	m/z	Intensity	Relative Intensity (%)
1	29	1627	1.859	57	67.2	218	0.249
2	29	7268	8.303	58	67.2	204	0.233
3	29.1	40	0.046	59	68	285	0.326
4	29.1	28	0.032	60	68.1	14663	16.751
5	29.1	162	0.185	61	68.1	138	0.158
6	31	3003	3.431	62	68.1	547	0.625
7	37	174	0.199	63	69.1	5543	6.332
8	38	303	0.346	64	69.1	16233	18.544
9	39	19010	21.717	65	69.2	417	0.476
10	39.1	358	0.409	66	70.1	3087	3.527
11	40	5257	6.005	67	70.1	1339	1.530
12	41.1	41934	47.904	68	71.1	87537	100.000
13	41.1	1140	1.302	69	71.1	74	0.085
14	41.1	331	0.378	70	71.2	458	0.523
15	41.2	198	0.226	71	71.4	125	0.143
16	41.3	70	0.080	72	71.4	142	0.162
17	41.3	183	0.209	73	71.5	393	0.449
18	41.6	56	0.064	74	71.5	236	0.270
19	41.6	73	0.083	75	71.7	85	0.097
20	41.9	101	0.115	76	71.7	72	0.082
21	42	1450	1.656	77	71.8	117	0.134
22	42.1	5094	5.819	78	71.8	67	0.077
23	43	27333	31.225	79	71.8	100	0.114
24	43.1	30516	34.861	80	72.1	5262	6.011
25	43.1	921	1.052	81	72.1	205	0.234
26	43.3	94	0.107	82	73.1	466	0.532
27	43.3	140	0.160	83	75.1	199	0.227
28	43.4	222	0.254	84	77.1	14318	16.357
29	43.6	241	0.275	85	77.1	158	0.180
30	44	1471	1.680	86	77.2	120	0.137
31	44.1	1167	1.333	87	78.1	2953	3.373
32	45	2633	3.008	88	79.1	7525	8.596
33	50	1025	1.171	89	79.1	158	0.180
34	51	3587	4.098	90	80.1	1419	1.621
35	52	2368	2.705	91	81.1	10936	12.493
36	53	138	0.158	92	81.2	208	0.238
37	53.1	15315	17.495	93	82.1	736	0.841
38	53.1	129	0.147	94	82.1	2827	3.229
39	53.1	237	0.271	95	83.1	2109	2.409
40	54.1	2776	3.171	96	83.1	8045	9.190
41	55	11300	12.909	97	84.1	1697	1.939
42	55.1	14677	16.767	98	84.1	787	0.899
43	55.2	283	0.323	99	85	209	0.239
44	56	969	1.107	100	85.1	1238	1.414
45	56.1	1525	1.742	101	86.1	19437	22.204
46	57.1	3536	4.039	102	86.2	540	0.617
47	57.1	957	1.093	103	86.2	188	0.215
48	58.1	1742	1.990	104	87.1	1742	1.990
49	59.1	1691	1.932	105	89.1	263	0.300
50	63	1163	1.329	106	91.1	14777	16.881
51	64.1	395	0.451	107	91.1	99	0.113
52	65.1	6209	7.093	108	91.2	73	0.083
53	66.1	2433	2.779	109	91.2	42	0.048
54	67	134	0.153	110	91.4	117	0.134
55	67.1	21255	24.281	111	92.1	6643	7.589
56	67.1	519	0.593	112	92.1	84	0.096

Table S14. Cont.

Peak#	m/z	Intensity	Relative Intensity (%)
113	93.1	89	0.102
114	93.1	44118	50.399
115	93.2	587	0.671
116	93.5	302	0.345
117	93.6	141	0.161
118	93.6	116	0.133
119	93.6	148	0.169
120	94.1	745	0.851
121	94.1	4535	5.181
122	95.1	6898	7.880
123	95.1	2154	2.461
124	96.1	1336	1.526
125	96.1	269	0.307
126	97.1	1829	2.089
127	98.1	1104	1.261
128	99.1	269	0.307
129	102.1	166	0.190
130	103.1	656	0.749
131	104.1	353	0.403
132	105.1	1707	1.950
133	106.1	326	0.372
134	107.1	769	0.878
135	107.1	1092	1.247
136	108.1	239	0.273
137	108.1	233	0.266
138	109.1	1180	1.348
139	109.1	269	0.307
140	110.1	5556	6.347
141	111.1	37475	42.810
142	111.2	263	0.300
143	111.3	143	0.163
144	111.6	118	0.135
145	112.1	3496	3.994
146	113.1	147	0.168
147	115.1	716	0.818
148	117.1	620	0.708
149	119.1	1940	2.216
150	121.1	2966	3.388
151	122.1	277	0.316
152	123.1	534	0.610
153	124.1	164	0.187
154	125.1	2053	2.345
155	126.1	199	0.227
156	134.2	595	0.680
157	135.1	1066	1.218
158	136.2	6217	7.102
159	136.2	104	0.119
160	137.2	751	0.858
161	139.2	2181	2.492
162	140.2	172	0.196
163	154.2	10391	11.870
164	154.3	282	0.322
165	155.2	1437	1.642
166	187	13	0.015
167	299.5	195	0.223

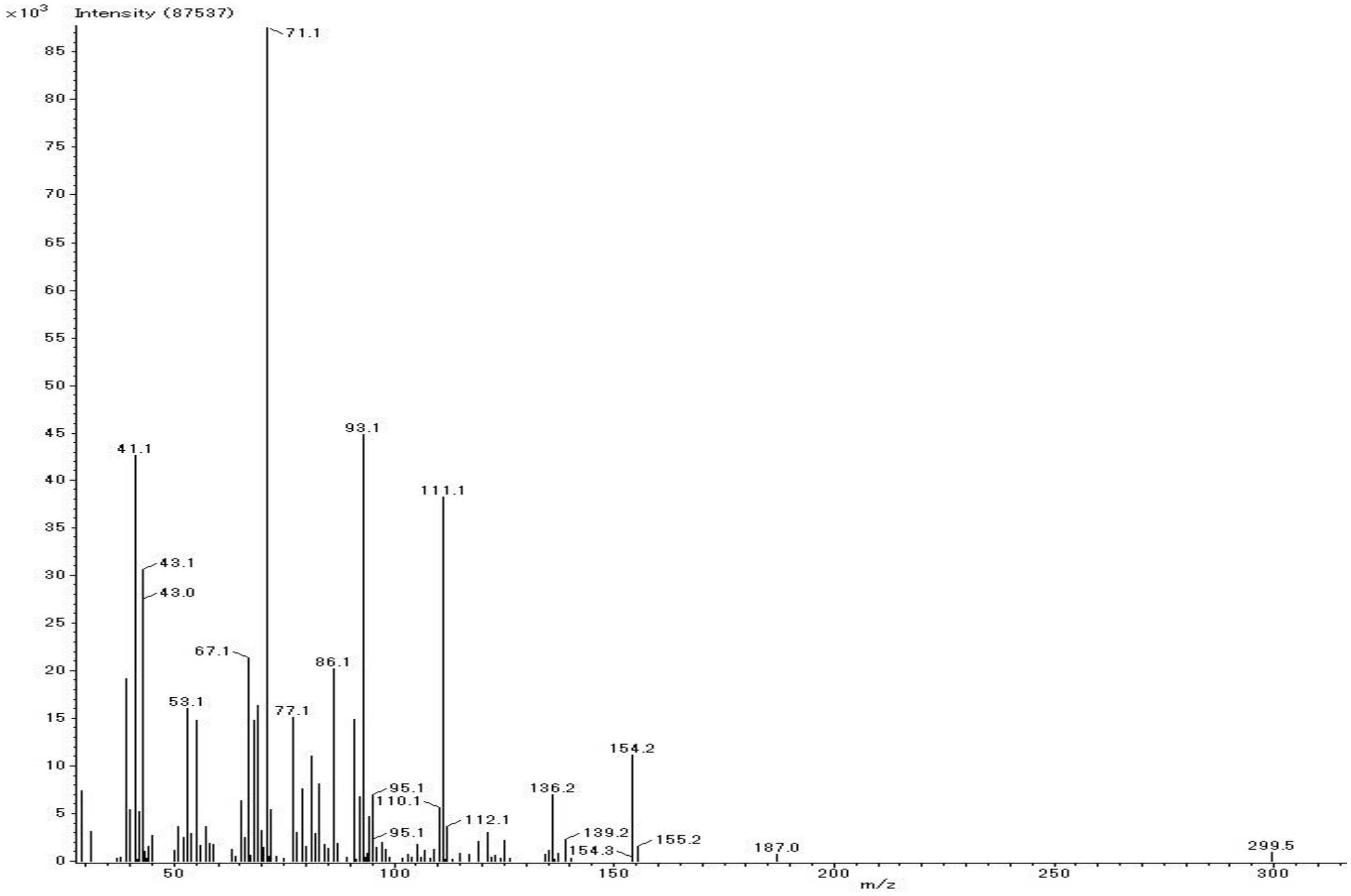

Figure S16a. Mass-spectra of Terpinen-4-ol (Rt= 7.81) detected in *P. nigrum* essential oil by GC- MS

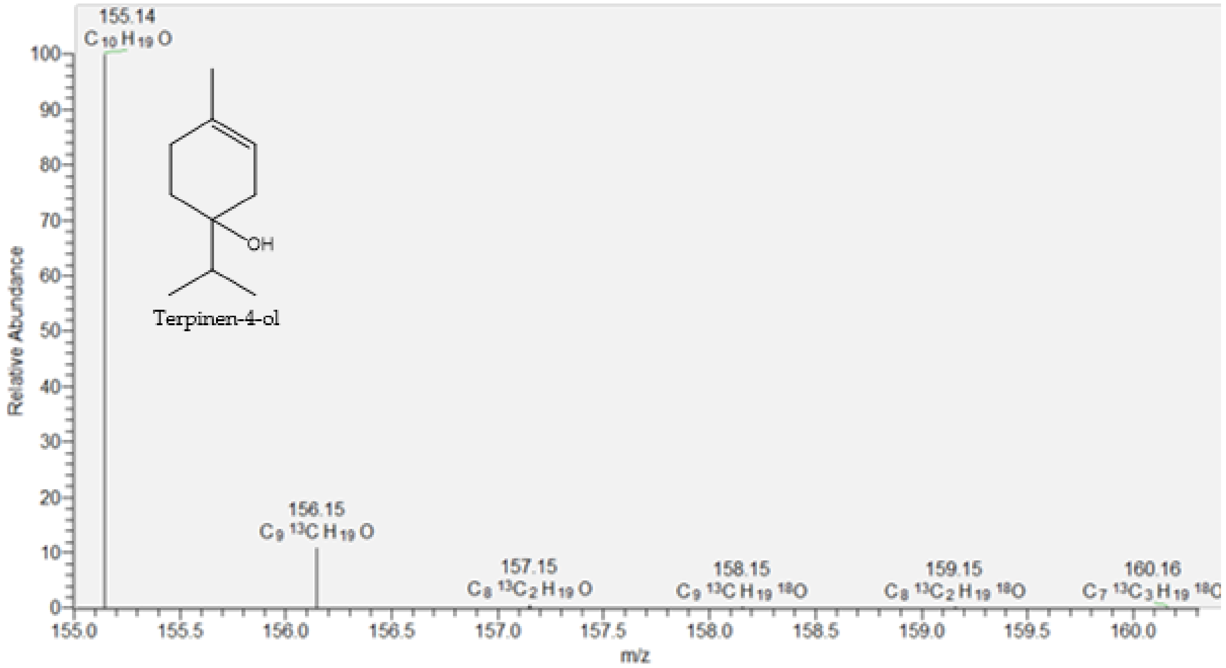

Figure S16b. Spectra of β-Thujene in *P. nigrum* essential oil identified by positive ion (H^+) adduction method of LC-ESI-MS

Table S15. Fragmentation pattern of δ-EIemene (retention time = 9.96) in *P. nigrum* essential oil detected by GC-MS

Peak#	m/z	Intensity	Relative Intensity (%)	Peak#	m/z	Intensity	Relative Intensity (%)
1	29	253	1.997	30	92.1	1714	13.528
2	29	1377	10.868	31	93.1	9446	74.554
3	29.1	417	3.291	32	94.1	1550	12.234
4	36	119	0.939	33	95.1	610	4.815
5	38	425	3.354	34	103.1	658	5.193
6	38	386	3.047	35	105.1	3514	27.735
7	39	2161	17.056	36	106.1	844	6.661
8	40	952	7.514	37	107.1	2231	17.609
9	41.1	5768	45.525	38	108.1	445	3.512
10	42.1	716	5.651	39	109.1	386	3.047
11	43.1	2352	18.564	40	115.1	759	5.991
12	51	1039	8.200	41	117.1	718	5.667
13	52	838	6.614	42	119.1	1659	13.094
14	53.1	2405	18.982	43	120.1	618	4.878
15	55.1	1855	14.641	44	121.1	601	4.743
16	57.1	686	5.414	45	121.1	12670	100.000
17	62	485	3.828	46	122.1	934	7.372
18	63	384	3.031	47	129.1	506	3.994
19	65.1	1650	13.023	48	133.1	1065	8.406
20	67.1	2043	16.125	49	136.2	6402	50.529
21	68.1	1041	8.216	50	137.2	767	6.054
22	69.1	751	5.927	51	147.2	512	4.041
23	72.1	312	2.463	52	149.1	1218	9.613
24	77.1	4108	32.423	53	150.1	642	5.067
25	78.1	1364	10.766	54	161.2	2977	23.496
26	79.1	3689	29.116	55	162.2	795	6.275
27	80.1	898	7.088	56	187	136	1.073
28	81.1	1432	11.302	57	189.2	652	5.146
29	91.1	5073	40.039				

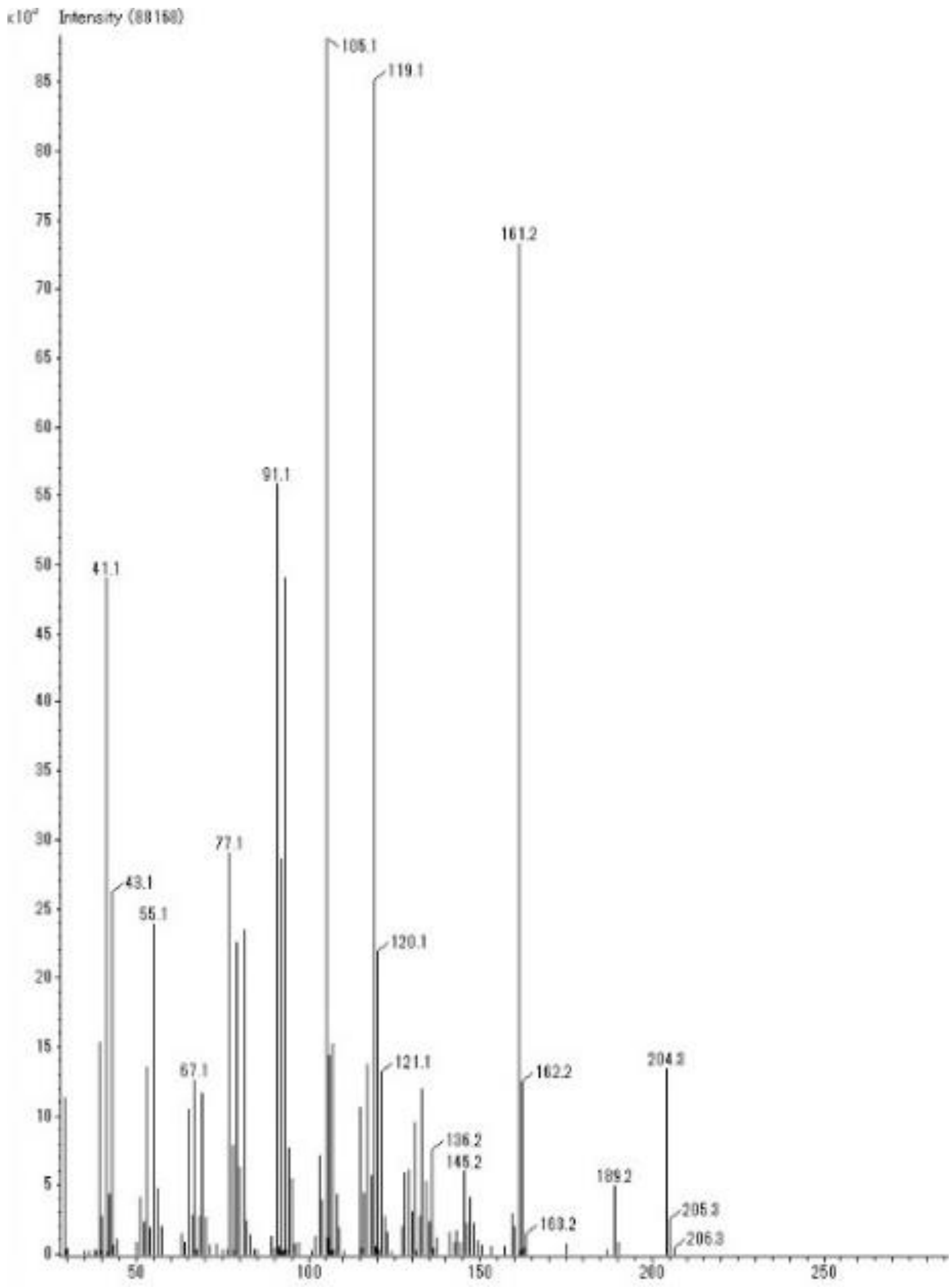

Figure S17a. Mass-spectra of δ-EIemene (Rt= 9.96) detected in *P. nigrum* essential oil by GC- MS

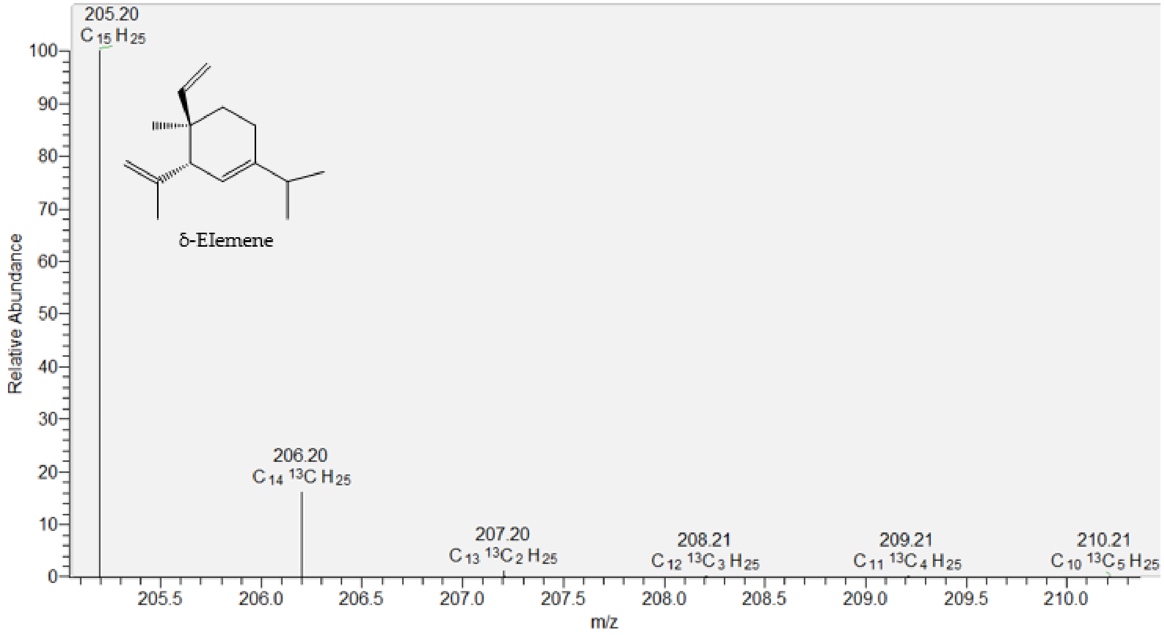

Figure S17b. Spectra of δ-EIemene in *P. nigrum* essential oil identified by positive ion (H^+) adduction method of LC-ESI-MS

Table S16. Fragmentation pattern of alpha-Copaene (retention time = 10.55) in *P. nigrum* essential oil detected by GC-MS

Peak#	m/z	Intensity	Relative intensity (%)	Peak#	m/z	Intensity	Relative intensity (%)
1	29	11237	12.745	54	77.1	28334	32.136
2	29.1	439	0.498	55	77.2	225	0.255
3	29.2	128	0.145	56	78.1	7747	8.787
4	29.5	126	0.143	57	78.1	121	0.137
5	30	331	0.375	58	79.1	22431	25.441
6	35	129	0.146	59	79.2	507	0.575
7	36	70	0.079	60	80.1	6158	6.984
8	38	124	0.141	61	80.2	212	0.240
9	38	185	0.210	62	81.1	23368	26.504
10	38.3	139	0.158	63	81.2	202	0.229
11	39	15159	17.193	64	81.2	200	0.227
12	39.1	437	0.496	65	82.1	2341	2.655
13	39.3	102	0.116	66	83.1	1230	1.395
14	40	32	0.036	67	84.1	228	0.259
15	40	2610	2.960	68	85.1	227	0.257
16	41.1	48334	54.820	69	89.1	1129	1.281
17	41.1	1305	1.480	70	90.1	321	0.364
18	41.3	442	0.501	71	91.1	55102	62.497
19	41.3	221	0.251	72	91.2	387	0.439
20	41.6	42	0.048	73	91.3	135	0.153
21	42.1	4217	4.783	74	91.4	375	0.425
22	43	287	0.326	75	91.5	230	0.261
23	43.1	26113	29.617	76	91.5	324	0.367
24	43.1	531	0.602	77	91.6	252	0.286
25	44	72	0.082	78	91.6	106	0.120
26	44.1	923	1.047	79	91.9	77	0.087
27	50	750	0.851	80	92.1	28466	32.286
28	51	4002	4.539	81	92.2	312	0.354
29	52	2161	2.451	82	92.2	160	0.181
30	53.1	13421	15.222	83	92.6	59	0.067
31	53.1	198	0.225	84	93.1	48960	55.530
32	54.1	1770	2.008	85	93.2	642	0.728
33	55.1	23164	26.273	86	93.3	135	0.153
34	55.1	405	0.459	87	93.5	111	0.126
35	55.2	157	0.178	88	93.6	216	0.245
36	56.1	4595	5.212	89	94.1	7535	8.546
37	57.1	1913	2.170	90	95.1	5322	6.036
38	63	1342	1.522	91	96.1	632	0.717
39	64.1	721	0.818	92	97.1	749	0.850
40	65.1	10376	11.768	93	101.1	117	0.133
41	65.1	163	0.185	94	102.1	1138	1.291
42	66.1	2756	3.126	95	103.1	7022	7.964
43	67.1	11798	13.381	96	104.1	3754	4.258
44	67.1	261	0.296	97	105.1	88168	100.000
45	68.1	2583	2.930	98	105.2	131	0.149
46	69.1	11578	13.132	99	105.5	1044	1.184
47	69.2	155	0.176	100	105.6	680	0.771
48	70.1	2542	2.883	101	106	127	0.144
49	71.1	142	0.161	102	106	231	0.262
50	71.1	505	0.573	103	106.1	14286	16.203
51	73.1	578	0.656	104	106.2	134	0.152
52	75	191	0.217	105	106.3	105	0.119
53	76.1	209	0.237	106	106.4	88	0.100

Table S16. Cont.

Peak#	m/z	Intensity	Relative intensity (%)	Peak#	m/z	Intensity	Relative intensity (%)
107	106.4	63	0.071	160	147.2	3944	4.4733
108	106.4	88	0.100	161	148.2	2103	2.3852
109	107.1	15141	17.173	162	149.2	863	0.9788
110	107.2	254	0.288	163	150.2	491	0.5569
111	107.2	151	0.171	164	153.1	379	0.4299
112	108.1	4207	4.772	165	157.2	386	0.4378
113	109.1	1833	2.079	166	159.2	2807	3.1837
114	110.1	134	0.152	167	160.2	1929	2.1879
115	115.1	10449	11.851	168	161.2	72587	82.3281
116	115.2	307	0.348	169	161.3	124	0.1406
117	116.1	4275	4.849	170	161.4	105	0.1191
118	117.1	13666	15.500	171	161.4	121	0.1372
119	117.2	255	0.289	172	161.7	103	0.1168
120	118.1	5549	6.294	173	161.8	149	0.1690
121	119.1	85046	96.459	174	161.8	133	0.1508
122	119.2	157	0.178	175	161.9	80	0.0907
123	119.3	233	0.264	176	162.2	12364	14.0232
124	119.3	451	0.512	177	162.3	167	0.1894
125	119.6	204	0.231	178	162.4	210	0.2382
126	119.6	342	0.388	179	162.7	323	0.3663
127	119.7	454	0.515	180	163.2	1358	1.5402
128	120	79	0.090	181	175.2	666	0.7554
129	120.1	21800	24.726	182	187	228	0.2586
130	120.2	114	0.129	183	189.2	4222	4.7886
131	120.3	123	0.140	184	190.2	710	0.8053
132	120.5	194	0.220	185	204.3	12689	14.3918
133	120.5	103	0.117	186	205.3	2456	2.7856
134	121.1	13140	14.903	187	206.3	347	0.3936
135	121.3	91	0.103	188	626.6	127	0.1440
136	122.1	2639	2.993				
137	123.2	1495	1.696				
138	124.2	139	0.158				
139	127.1	1868	2.119				
140	128.1	5798	6.576				
141	129.1	6025	6.834				
142	130.1	2982	3.382				
143	131.1	9428	10.693				
144	131.2	115	0.1304				
145	132.1	2607	2.9569				
146	133.1	11834	13.4221				
147	133.2	203	0.2302				
148	134.2	5095	5.7787				
149	134.3	85	0.0964				
150	135.2	2246	2.5474				
151	136.2	7459	8.4600				
152	136.2	309	0.3505				
153	137.2	1068	1.2113				
154	141.1	1430	1.6219				
155	142.1	771	0.8745				
156	143.1	1569	1.7796				
157	144.1	713	0.8087				
158	145.2	5190	5.8865				
159	146.2	2082	2.3614				

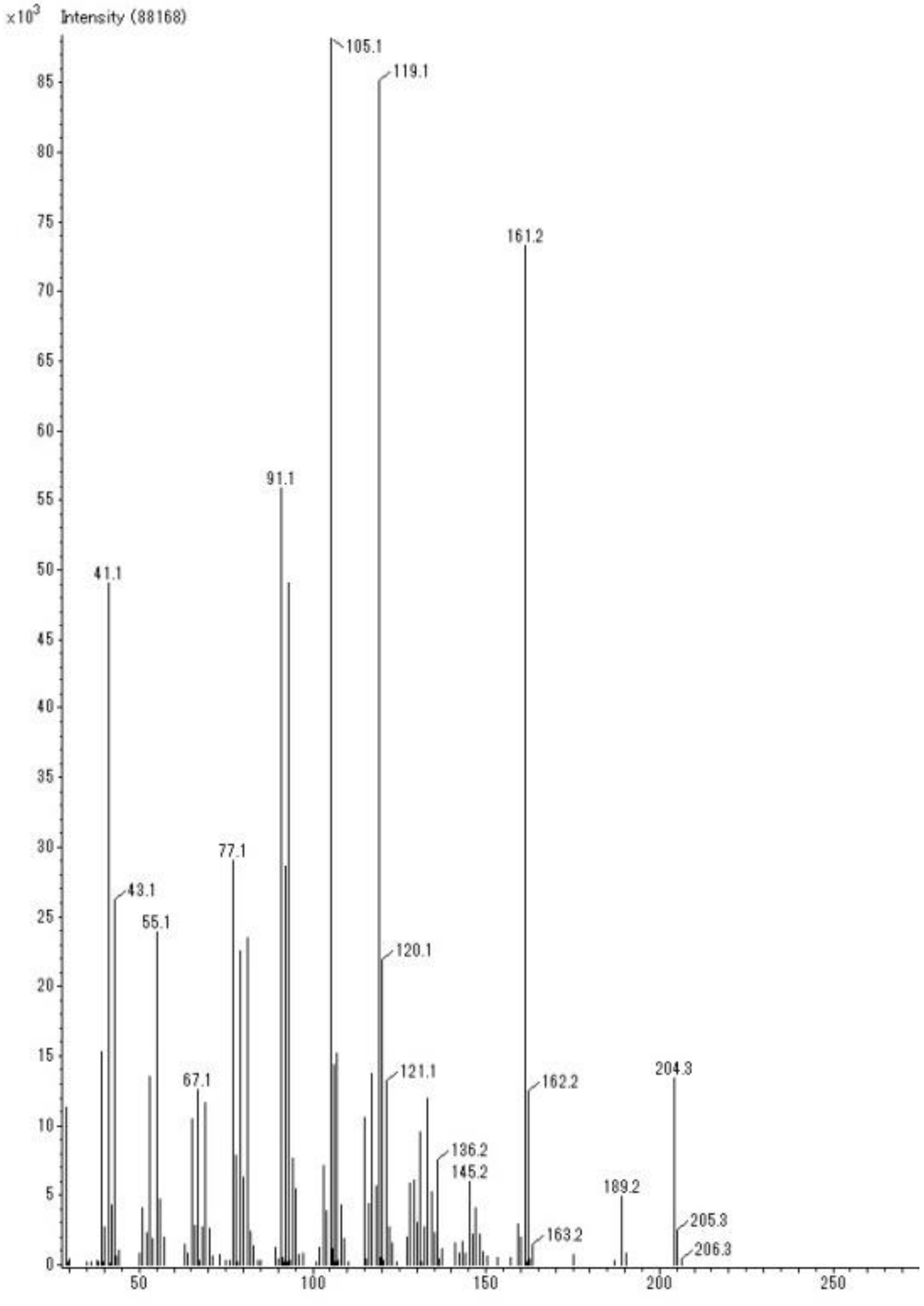

Figure S18a. Mass-spectra of alpha-Copaene (Rt= 10.55) detected in *P. nigrum* essential oil by GC- MS

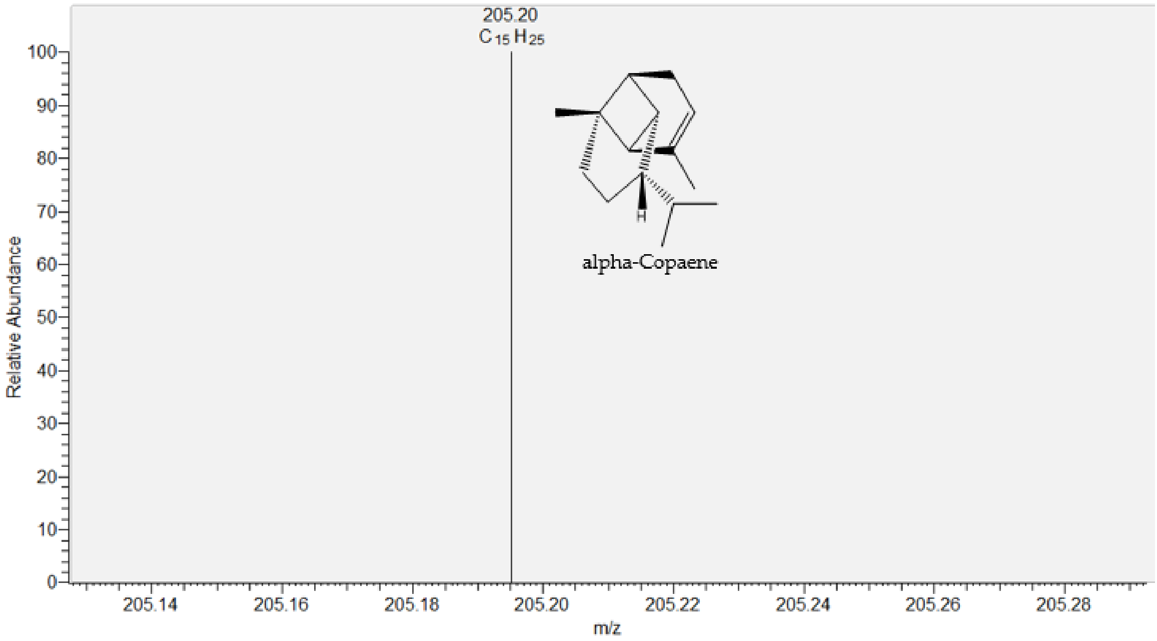

Figure S18b. Spectra of alpha-Copaene in *P. nigrum* essential oil identified by positive ion (H^+) adduction method of LC-ESI-MS

Table S17. Fragmentation pattern of β-Caryophyllene (retention time = 11.23) in *P. nigrum* essential oil detected by GC-MS

Peak#	m/z	Intensity	Relative Intensity (%)	Peak#	m/z	Intensity	Relative Intensity (%)
1	29	101034	18.170	54	41	188	0.034
2	29.1	439	0.079	55	41.1	556044	100.000
3	29.3	352	0.063	56	41.2	1236	0.222
4	29.3	1138	0.205	57	41.2	295	0.053
5	29.3	916	0.165	58	41.3	293	0.053
6	29.3	245	0.044	59	41.3	1089	0.196
7	29.5	151	0.027	60	41.3	5123	0.921
8	29.5	589	0.106	61	41.4	2844	0.511
9	29.5	261	0.047	62	41.5	54	0.010
10	29.7	195	0.035	63	41.6	8262	1.486
11	29.7	296	0.053	64	41.8	59	0.011
12	29.7	211	0.038	65	41.8	5899	1.061
13	29.9	111	0.020	66	41.9	123	0.022
14	30.1	2406	0.433	67	42.1	33715	6.063
15	30.1	43	0.008	68	42.1	266	0.048
16	31.2	54	0.010	69	42.2	56	0.010
17	32	90	0.016	70	42.3	137	0.025
18	32	214	0.038	71	42.3	2152	0.387
19	32.1	33	0.006	72	42.4	31	0.006
20	32.2	46	0.008	73	42.5	145	0.026
21	32.2	70	0.013	74	42.6	1832	0.329
22	35	28	0.005	75	42.6	111	0.020
23	36	162	0.029	76	42.8	558	0.100
24	37	407	0.073	77	42.9	586	0.105
25	38	2558	0.460	78	42.9	126	0.023
26	39	179991	32.370	79	43	373	0.067
27	39.1	292	0.053	80	43.1	58074	10.444
28	39.2	577	0.104	81	43.1	235	0.042
29	39.2	49	0.009	82	43.2	135	0.024
30	39.3	499	0.090	83	43.2	84	0.015
31	39.3	2492	0.448	84	43.3	322	0.058
32	39.5	365	0.066	85	43.4	452	0.081
33	39.6	827	0.149	86	43.4	179	0.032
34	39.6	176	0.032	87	43.4	68	0.012
35	39.8	35	0.006	88	43.6	135	0.024
36	39.8	747	0.134	89	43.6	201	0.036
37	39.8	335	0.060	90	43.6	112	0.020
38	39.9	101	0.018	91	43.9	126	0.023
39	40	167	0.030	92	44	271	0.049
40	40	50467	9.076	93	44.1	2211	0.398
41	40.1	925	0.166	94	44.1	99	0.018
42	40.3	129	0.023	95	44.1	127	0.023
43	40.3	304	0.055	96	50	5309	0.955
44	40.3	325	0.058	97	51	33787	6.076
45	40.3	15	0.003	98	51.1	1093	0.197
46	40.4	250	0.045	99	51.2	104	0.019
47	40.5	39	0.007	100	51.3	135	0.024
48	40.6	193	0.035	101	51.4	45	0.008
49	40.6	33	0.006	102	52	31793	5.718
50	40.8	23	0.004	103	52.1	759	0.136
51	40.8	153	0.028	104	52.4	71	0.013
52	40.8	100	0.018	105	53.1	170317	30.630
53	40.8	49	0.009	106	53.1	134	0.024

Table S17. Cont.

Peak#	m/z	Intensity	Relative Intensity (%)	Peak#	m/z	Intensity	Relative Intensity (%)
107	53.2	444	0.080	160	65.7	261	0.047
108	53.3	311	0.056	161	65.8	230	0.041
109	53.4	843	0.152	162	65.8	71	0.013
110	53.4	951	0.171	163	66.1	30904	5.558
111	53.6	138	0.025	164	66.1	173	0.031
112	53.7	994	0.179	165	66.2	107	0.019
113	53.9	89	0.016	166	66.2	110	0.020
114	54	208	0.037	167	66.4	86	0.015
115	54	196	0.035	168	66.5	63	0.011
116	54.1	23757	4.273	169	67	15	0.003
117	54.1	237	0.043	170	67.1	193759	34.846
118	54.2	226	0.041	171	67.1	175	0.031
119	54.2	168	0.030	172	67.2	351	0.063
120	54.3	20	0.004	173	67.3	39	0.007
121	54.4	38	0.007	174	67.3	238	0.043
122	54.5	48	0.009	175	67.4	468	0.084
123	55.1	176882	31.811	176	67.5	1630	0.293
124	55.1	309	0.056	177	67.5	1119	0.201
125	55.2	863	0.155	178	67.7	93	0.017
126	55.4	491	0.088	179	67.8	536	0.096
127	55.4	1172	0.211	180	67.8	54	0.010
128	55.4	895	0.161	181	68	130	0.023
129	55.5	119	0.021	182	68.1	55901	10.053
130	55.6	19	0.003	183	68.2	231	0.042
131	55.7	996	0.179	184	68.2	192	0.035
132	55.7	44	0.008	185	68.4	385	0.069
133	55.8	30	0.005	186	68.4	564	0.101
134	56	518	0.093	187	68.5	237	0.043
135	56	32	0.006	188	68.5	299	0.054
136	56.1	57570	10.353	189	68.7	74	0.013
137	56.2	479	0.086	190	68.7	82	0.015
138	56.3	215	0.039	191	68.7	462	0.083
139	56.3	89	0.016	192	69	176	0.032
140	56.4	46	0.008	193	69.1	314978	56.646
141	56.5	61	0.011	194	69.3	669	0.120
142	57.1	23732	4.268	195	69.3	45	0.008
143	57.1	580	0.104	196	69.4	126	0.023
144	58.1	1209	0.217	197	69.4	745	0.134
145	58.1	1157	0.208	198	69.5	2252	0.405
146	59.1	1041	0.187	199	69.5	1741	0.313
147	60.1	302	0.054	200	69.8	137	0.025
148	62	877	0.158	201	69.8	3463	0.623
149	63	8539	1.536	202	69.8	336	0.060
150	63.1	70	0.013	203	69.9	76	0.014
151	64.1	3898	0.701	204	70	104	0.019
152	64.6	90	0.016	205	70.1	21250	3.822
153	65.1	89418	16.081	206	70.2	395	0.071
154	65.2	985	0.177	207	70.4	84	0.015
155	65.2	92	0.017	208	70.4	415	0.075
156	65.3	702	0.126	209	70.5	922	0.166
157	65.4	482	0.087	210	70.5	50	0.009
158	65.4	311	0.056	211	70.7	202	0.036
159	65.5	156	0.028	212	70.7	70	0.013

Table S17. Cont.

Peak#	m/z	Intensity	Relative Intensity (%)	Peak#	m/z	Intensity	Relative Intensity (%)
213	70.8	546	0.098	266	81.4	139	0.025
214	71.1	687	0.124	267	81.5	239	0.043
215	71.1	9385	1.688	268	81.5	2259	0.406
216	71.2	186	0.033	269	81.8	402	0.072
217	71.4	60	0.011	270	81.9	1357	0.244
218	71.4	52	0.009	271	81.9	284	0.051
219	71.7	48	0.009	272	82.1	37720	6.784
220	72.1	212	0.038	273	82.2	1073	0.193
221	72.1	513	0.092	274	82.3	141	0.025
222	73.1	163	0.029	275	82.5	213	0.038
223	74.1	325	0.058	276	82.6	322	0.058
224	75	1055	0.190	277	82.6	44	0.008
225	76.1	1582	0.285	278	82.8	65	0.012
226	77.1	201889	36.308	279	82.9	244	0.044
227	77.2	120	0.022	280	82.9	220	0.040
228	77.3	1536	0.276	281	83	253	0.045
229	77.4	136	0.024	282	83.1	11349	2.041
230	77.5	1071	0.193	283	83.2	723	0.130
231	77.5	1080	0.194	284	83.3	82	0.015
232	77.7	102	0.018	285	83.6	63	0.011
233	77.8	1753	0.315	286	84.1	1350	0.243
234	77.9	162	0.029	287	85	119	0.021
235	78.1	54070	9.724	288	85.1	123	0.022
236	78.1	421	0.076	289	87.1	108	0.019
237	78.2	256	0.046	290	89.1	3846	0.692
238	78.4	128	0.023	291	90.1	1462	0.263
239	78.5	1176	0.211	292	91	61	0.011
240	78.5	313	0.056	293	91	109	0.020
241	78.6	79	0.014	294	91.1	343483	61.773
242	78.7	190	0.034	295	91.4	2511	0.452
243	78.8	711	0.128	296	91.4	505	0.091
244	78.9	223	0.040	297	91.5	1890	0.340
245	78.9	58	0.010	298	91.6	1181	0.212
246	79.1	330513	59.440	299	91.6	32	0.006
247	79.2	293	0.053	300	91.8	125	0.022
248	79.4	1403	0.252	301	91.9	2765	0.497
249	79.4	591	0.106	302	92	111	0.020
250	79.5	2330	0.419	303	92.1	86457	15.549
251	79.5	4454	0.801	304	92.2	614	0.110
252	79.8	203	0.037	305	92.3	90	0.016
253	79.8	3681	0.662	306	92.3	150	0.027
254	79.9	190	0.034	307	92.5	204	0.037
255	80.1	73251	13.174	308	92.6	2011	0.362
256	80.2	186	0.033	309	92.7	245	0.044
257	80.3	107	0.019	310	92.9	855	0.154
258	80.4	155	0.028	311	93	36	0.006
259	80.5	79	0.014	312	93.1	327599	58.916
260	80.5	2512	0.452	313	93.3	400	0.072
261	80.8	1210	0.218	314	93.4	257	0.046
262	80.9	230	0.041	315	93.4	154	0.028
263	81.1	139259	25.045	316	93.5	712	0.128
264	81.2	161	0.029	317	93.5	1928	0.347
265	81.3	519	0.093	318	93.6	1567	0.282

Table S17. Cont.

Peak#	m/z	Intensity	Relative Intensity (%)	Peak#	m/z	Intensity	Relative Intensity (%)
319	93.9	604	0.109	372	107.4	24	0.004
320	93.9	1884	0.339	373	107.4	82	0.015
321	94	82	0.015	374	107.5	267	0.048
322	94.1	65486	11.777	375	107.6	346	0.062
323	94.2	491	0.088	376	107.6	500	0.090
324	94.3	177	0.032	377	107.9	142	0.026
325	94.4	161	0.029	378	108	277	0.050
326	94.6	923	0.166	379	108	288	0.052
327	94.7	985	0.177	380	108.1	31358	5.639
328	94.9	61	0.011	381	108.2	275	0.049
329	95	134	0.024	382	108.3	280	0.050
330	95	191	0.034	383	108.3	32	0.006
331	95	239	0.043	384	108.3	336	0.060
332	95.1	65087	11.705	385	108.4	65	0.012
333	95.2	625	0.112	386	109.1	50914	9.156
334	95.3	130	0.023	387	109.2	83	0.015
335	95.4	33	0.006	388	109.3	269	0.048
336	95.5	43	0.008	389	109.6	112	0.020
337	95.5	70	0.013	390	109.7	38	0.007
338	95.6	40	0.007	391	109.9	25	0.004
339	95.9	94	0.017	392	110	30	0.005
340	96.1	9269	1.667	393	110.1	8306	1.494
341	97.1	3752	0.675	394	111.1	86	0.015
342	98.1	208	0.037	395	111.2	8733	1.571
343	101.1	407	0.073	396	111.2	161	0.029
344	102.1	2644	0.476	397	112.2	1392	0.250
345	103.1	21496	3.866	398	113.2	360	0.065
346	103.2	92	0.017	399	115.1	20138	3.622
347	103.2	81	0.015	400	115.2	446	0.080
348	103.3	70	0.013	401	115.2	343	0.062
349	103.4	156	0.028	402	116.1	7866	1.415
350	104.1	10825	1.947	403	117.1	26583	4.781
351	105.1	195479	35.155	404	117.3	277	0.050
352	105.2	318	0.057	405	117.4	73	0.013
353	105.3	188	0.034	406	117.6	33	0.006
354	105.4	228	0.041	407	118.1	7068	1.271
355	105.4	43	0.008	408	118.2	27	0.005
356	105.5	335	0.060	409	119.1	108220	19.462
357	105.6	471	0.085	410	119.3	125	0.022
358	105.6	1815	0.326	411	119.3	108	0.019
359	106	642	0.115	412	119.5	183	0.033
360	106.1	98786	17.766	413	119.6	768	0.138
361	106.2	242	0.044	414	119.7	523	0.094
362	106.3	376	0.068	415	119.7	251	0.045
363	106.3	159	0.029	416	120	487	0.088
364	106.4	75	0.013	417	120.1	117400	21.113
365	106.5	25	0.004	418	120.3	244	0.044
366	106.6	36	0.006	419	120.5	83	0.015
367	106.6	211	0.038	420	120.5	189	0.034
368	107.1	137158	24.667	421	120.6	146	0.026
369	107.2	120	0.022	422	120.6	440	0.079
370	107.3	102	0.018	423	120.7	217	0.039
371	107.3	77	0.014	424	120.7	36	0.006

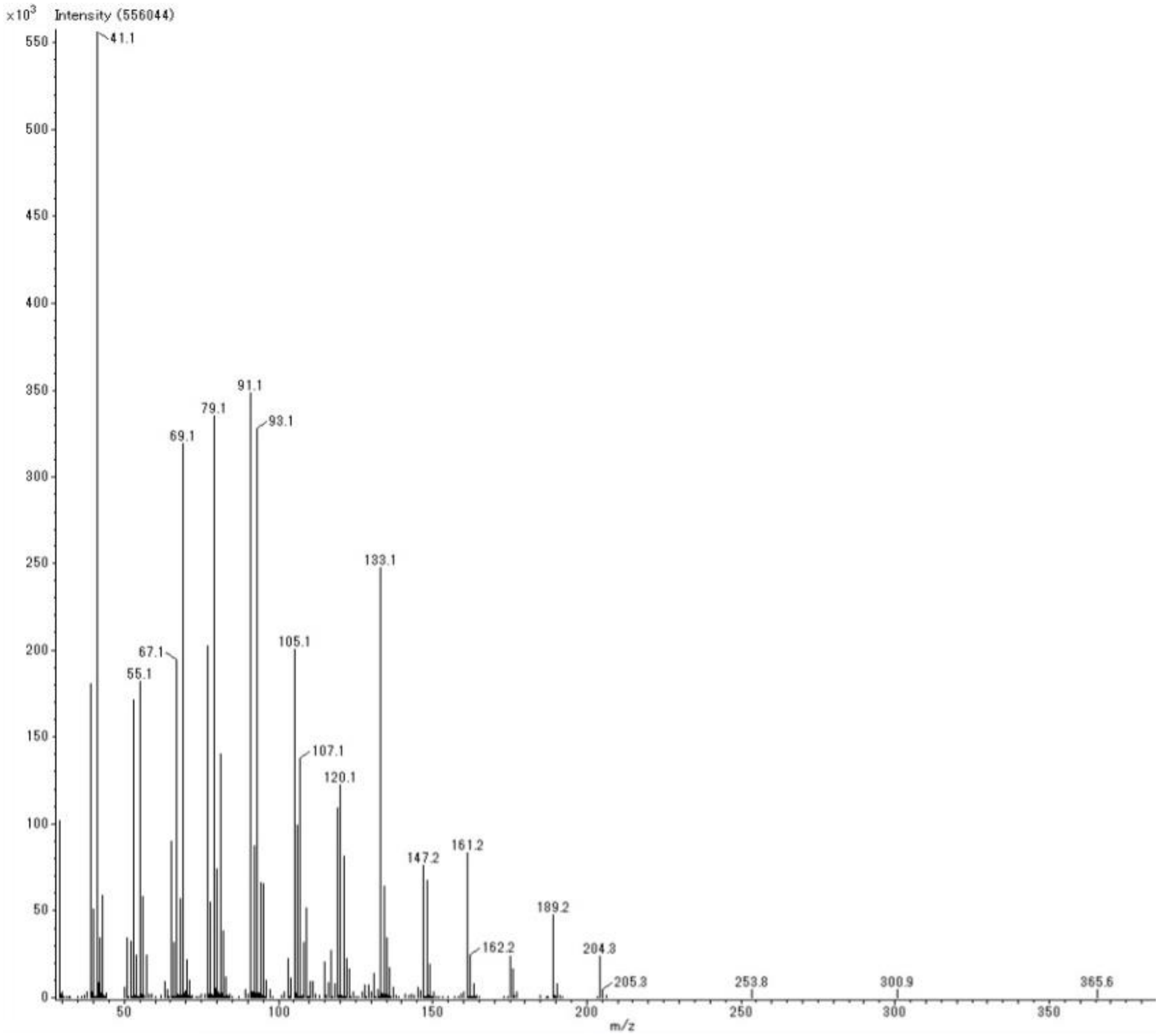

Figure S19a. Mass-spectra of β-Caryophyllene (Rt= 11.23) detected in *P. nigrum* essential oil by GC- MS

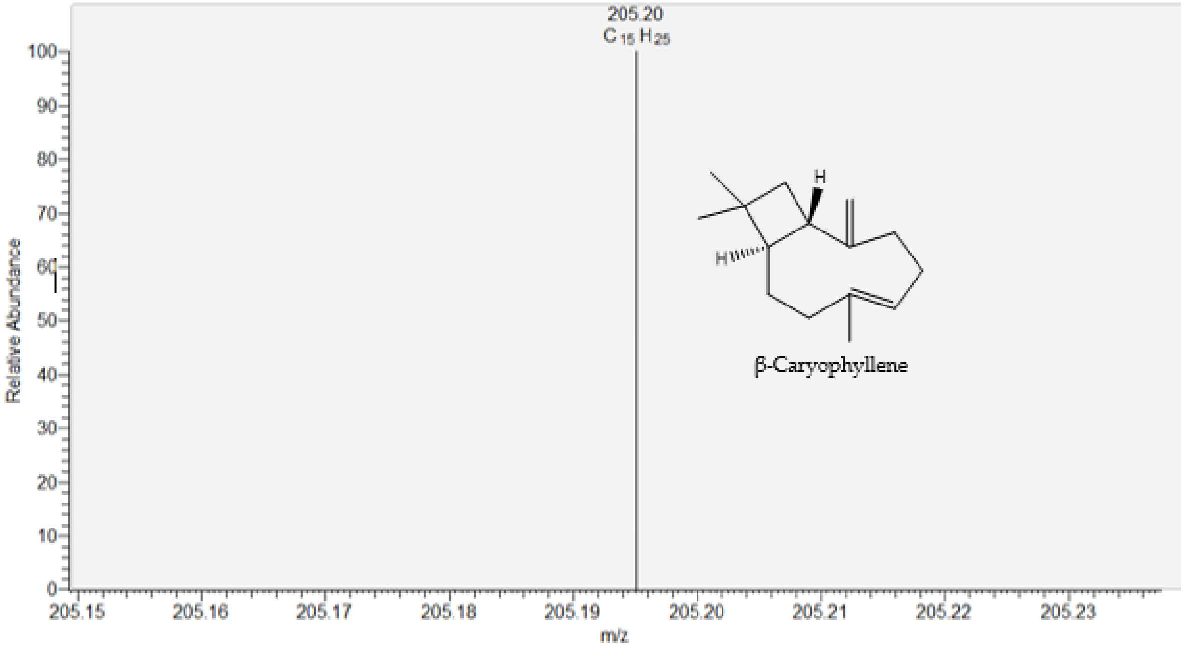

Figure S19b. Spectra of β-Caryophyllene in *P. nigrum* essential oil identified by positive ion (H^+) adduction method of LC-ESI-MS

Table S18. Fragmentation pattern of Humulene (retention time = 11.60) in *P. nigrum* essential oil detected by GC-MS

Peak#	m/z	Intensity	Relative Intensity (%)	Peak#	m/z	Intensity	Relative Intensity (%)
1	29	121	0.022	57	53.1	106540	19.723
2	29	62923	11.649	58	53.2	1262	0.234
3	29.1	448	0.083	59	53.4	337	0.062
4	29.2	180	0.033	60	53.4	177	0.033
5	29.2	83	0.015	61	53.4	408	0.076
6	29.3	239	0.044	62	53.6	48	0.009
7	29.3	434	0.080	63	53.6	213	0.039
8	29.5	288	0.053	64	53.7	338	0.063
9	29.5	49	0.009	65	53.7	215	0.040
10	30.1	1490	0.276	66	53.7	133	0.025
11	36	89	0.016	67	54	50	0.009
12	38	1232	0.228	68	54	63	0.012
13	39	78676	14.565	69	54.1	12593	2.331
14	39.1	732	0.136	70	54.2	38	0.007
15	39.2	115	0.021	71	54.2	43	0.008
16	39.3	338	0.063	72	55.1	86410	15.997
17	39.3	463	0.086	73	55.1	1065	0.197
18	39.3	286	0.053	74	55.2	193	0.036
19	39.4	108	0.020	75	55.4	173	0.032
20	39.5	58	0.011	76	55.4	51	0.009
21	40	25623	4.744	77	55.4	392	0.073
22	40.1	515	0.095	78	55.4	261	0.048
23	40.1	229	0.042	79	55.5	109	0.020
24	41.1	193186	35.764	80	55.7	372	0.069
25	41.2	785	0.145	81	56	112	0.021
26	41.3	320	0.059	82	56.1	7162	1.326
27	41.3	1139	0.211	83	57.1	3914	0.725
28	41.4	744	0.138	84	58	705	0.131
29	41.6	2588	0.479	85	59.1	2708	0.501
30	41.8	312	0.058	86	62	517	0.096
31	41.8	519	0.096	87	63	4166	0.771
32	41.9	252	0.047	88	64.1	1789	0.331
33	41.9	351	0.065	89	65.1	39028	7.225
34	42.1	17672	3.272	90	65.1	826	0.153
35	42.1	516	0.096	91	65.2	370	0.068
36	42.3	52	0.010	92	65.3	265	0.049
37	42.3	62	0.011	93	65.4	83	0.015
38	42.4	45	0.008	94	65.4	68	0.013
39	42.4	58	0.011	95	66.1	14364	2.659
40	43	280	0.052	96	66.1	296	0.055
41	43.1	55038	10.189	97	66.2	87	0.016
42	43.1	959	0.178	98	67.1	121385	22.472
43	43.2	326	0.060	99	67.2	2221	0.411
44	43.3	156	0.029	100	67.4	73	0.014
45	43.4	107	0.020	101	67.4	202	0.037
46	43.4	204	0.038	102	67.4	475	0.088
47	43.6	98	0.018	103	67.5	443	0.082
48	44	74	0.014	104	67.7	117	0.022
49	44.1	2173	0.402	105	67.8	815	0.151
50	45	2616	0.484	106	67.8	221	0.041
51	50	2487	0.460	107	68.1	44185	8.180
52	51	17160	3.177	108	68.2	659	0.122
53	51.1	313	0.058	109	68.4	50	0.009
54	52	11031	2.042	110	68.4	88	0.016
55	52.1	91	0.017	111	68.5	244	0.045
56	52.1	180	0.033	112	69.1	23548	4.359

Table S18. Cont.

Peak#	m/z	Intensity	Relative Intensity (%)	Peak#	m/z	Intensity	Relative Intensity (%)
113	69.2	561	0.104	169	81.9	138	0.026
114	70.1	2522	0.467	170	82	50	0.009
115	71.1	311	0.058	171	82.1	28255	5.231
116	71.1	223	0.041	172	82.2	80	0.015
117	72.1	210	0.039	173	82.2	663	0.123
118	73.1	64296	11.903	174	82.3	164	0.030
119	73.1	207	0.038	175	82.5	36	0.007
120	73.2	294	0.054	176	82.6	58	0.011
121	73.5	175	0.032	177	83	116	0.021
122	74.1	5542	1.026	178	83.1	4165	0.771
123	74.2	87	0.016	179	83.2	127	0.024
124	75.1	225	0.042	180	84	90	0.017
125	75.1	2855	0.529	181	84.1	360	0.067
126	76.1	906	0.168	182	85.1	282	0.052
127	77.1	107955	19.985	183	87.1	459	0.085
128	77.1	432	0.080	184	89.1	1921	0.356
129	77.2	290	0.054	185	90.1	780	0.144
130	77.3	633	0.117	186	91.1	136481	25.266
131	77.4	67	0.012	187	91.2	673	0.125
132	77.5	507	0.094	188	91.3	466	0.086
133	77.5	298	0.055	189	91.4	2765	0.512
134	77.6	55	0.010	190	91.4	435	0.081
135	77.7	89	0.016	191	91.5	1130	0.209
136	77.8	233	0.043	192	91.6	638	0.118
137	77.9	234	0.043	193	91.6	221	0.041
138	78.1	20255	3.750	194	91.7	40	0.007
139	78.1	425	0.079	195	91.9	506	0.094
140	78.2	214	0.040	196	91.9	161	0.030
141	78.5	276	0.051	197	92.1	89395	16.549
142	79.1	126250	23.372	198	92.2	175	0.032
143	79.2	1747	0.323	199	92.3	559	0.103
144	79.4	604	0.112	200	92.3	131	0.024
145	79.4	310	0.057	201	92.5	326	0.060
146	79.5	716	0.133	202	92.6	562	0.104
147	79.5	1449	0.268	203	92.9	24	0.004
148	79.6	125	0.023	204	92.9	365	0.068
149	79.8	214	0.040	205	93	994	0.184
150	79.8	1007	0.186	206	93	164	0.030
151	79.9	245	0.045	207	93.1	540169	100.000
152	80.1	181976	33.689	208	93.3	272	0.050
153	80.2	361	0.067	209	93.4	260	0.048
154	80.3	160	0.030	210	93.4	98	0.018
155	80.4	53	0.010	211	93.5	905	0.168
156	80.4	400	0.074	212	93.5	1461	0.270
157	80.5	1357	0.251	213	93.6	1684	0.312
158	80.5	1216	0.225	214	93.6	137	0.025
159	80.8	38	0.007	215	93.9	59	0.011
160	80.8	1026	0.190	216	93.9	7173	1.328
161	80.9	280	0.052	217	94	128	0.024
162	81.1	45073	8.344	218	94.1	67245	12.449
163	81.2	135	0.025	219	94.2	204	0.038
164	81.5	169	0.031	220	94.3	212	0.039
165	81.5	1671	0.309	221	94.4	84	0.016
166	81.6	251	0.046	222	94.5	25	0.005
167	81.9	93	0.017	223	94.6	196	0.036
168	81.9	257	0.048	224	94.7	1571	0.291

Table S18. Cont.

Peak#	m/z	Intensity	Relative Intensity (%)	Peak#	m/z	Intensity	Relative Intensity (%)
225	94.7	100	0.019	281	117.1	8642	1.600
226	94.9	101	0.019	282	117.2	127	0.024
227	95	700	0.130	283	118.1	1981	0.367
228	95.1	34784	6.439	284	119.1	35774	6.623
229	95.2	470	0.087	285	119.2	446	0.083
230	95.3	322	0.060	286	119.3	180	0.033
231	95.3	303	0.056	287	119.3	81	0.015
232	95.4	514	0.095	288	120.1	8502	1.574
233	95.5	55	0.010	289	121	61	0.011
234	96.1	4225	0.782	290	121.1	151066	27.966
235	96.2	174	0.032	291	121.2	431	0.080
236	97.1	909	0.168	292	121.3	179	0.033
237	98	83	0.015	293	121.5	339	0.063
238	101.1	100	0.019	294	121.7	1759	0.326
239	102.1	1410	0.261	295	122.1	102	0.019
240	103.1	10306	1.908	296	122.1	37277	6.901
241	103.2	212	0.039	297	122.3	92	0.017
242	104.1	3967	0.734	298	122.4	44	0.008
243	105.1	68443	12.671	299	122.5	80	0.015
244	105.2	276	0.051	300	122.5	31	0.006
245	105.3	104	0.019	301	122.5	87	0.016
246	105.5	46	0.009	302	122.6	202	0.037
247	105.5	35	0.006	303	123.2	10061	1.863
248	105.6	65	0.012	304	123.3	59	0.011
249	105.6	180	0.033	305	124.2	1202	0.223
250	106	62	0.011	306	127.1	1259	0.233
251	106.1	18340	3.395	307	128.1	3890	0.720
252	106.2	392	0.073	308	129.1	3275	0.606
253	106.2	70	0.013	309	130.1	1412	0.261
254	107.1	76525	14.167	310	131.1	968	0.179
255	107.2	197	0.036	311	131.1	4079	0.755
256	107.3	412	0.076	312	132.1	1033	0.191
257	107.5	73	0.014	313	133.1	1236	0.229
258	107.6	211	0.039	314	133.1	11503	2.130
259	107.6	268	0.050	315	133.2	210	0.039
260	107.7	128	0.024	316	133.3	103	0.019
261	108	24	0.004	317	134.2	9549	1.768
262	108.1	49	0.009	318	134.2	238	0.044
263	108.1	19554	3.620	319	135.2	9500	1.759
264	108.2	82	0.015	320	136.2	17019	3.151
265	108.3	40	0.007	321	136.3	155	0.029
266	109.1	44133	8.170	322	136.3	40	0.007
267	109.3	605	0.112	323	137.2	2171	0.402
268	109.6	51	0.009	324	141.1	1358	0.251
269	109.6	95	0.018	325	142.1	882	0.163
270	109.6	108	0.020	326	143.1	883	0.163
271	109.7	106	0.020	327	144.1	277	0.051
272	110	84	0.016	328	145.1	2014	0.373
273	110.1	62	0.011	329	146.2	3227	0.597
274	110.1	5721	1.059	330	147.1	19993	3.701
275	111.2	2024	0.375	331	147.2	56455	10.451
276	112.2	82	0.015	332	147.3	304	0.056
277	115.1	8787	1.627	333	147.3	310	0.057
278	115.2	307	0.057	334	147.7	188	0.035
279	116.1	3182	0.589	335	147.8	77	0.014
280	117.1	972	0.180	336	148.1	3444	0.638

Table S18. Cont.

Peak#	m/z	Intensity	Relative Intensity (%)	Peak#	m/z	Intensity	Relative Intensity (%)
337	148.2	14809	2.742	393	326.1	1148	0.213
338	148.3	220	0.041	394	327.1	13948	2.582
339	148.7	101	0.019	395	327.2	101	0.019
340	149.1	1946	0.360	396	328.1	4514	0.836
341	149.2	3375	0.625	397	329.1	3140	0.581
342	150.1	90	0.017	398	330.1	774	0.143
343	150.2	463	0.086	399	341.1	6030	1.116
344	155.2	87	0.016	400	341.3	103	0.019
345	157.2	321	0.059	401	342.1	2303	0.426
346	159.2	677	0.125	402	343.1	1719	0.318
347	160.2	331	0.061	403	344.1	299	0.055
348	161.2	13972	2.587	404	383.1	2827	0.523
349	161.3	298	0.055	405	384.1	1120	0.207
350	162.2	4274	0.791	406	385.1	1709	0.316
351	163.2	1107	0.205	407	386.1	884	0.164
352	173.2	105	0.019	408	399.1	5697	1.055
353	174.2	82	0.015	409	399.3	97	0.018
354	175.2	2576	0.477	410	400.1	2330	0.431
355	176.2	1230	0.228	411	401.1	2015	0.373
356	187	254	0.047	412	402.1	769	0.142
357	187.2	321	0.059	413	403.1	85	0.016
358	189.1	207	0.038	414	415.2	10355	1.917
359	189.2	11609	2.149	415	415.3	114	0.021
360	189.3	421	0.078	416	416.2	4368	0.809
361	190.2	1966	0.364	417	417.2	3071	0.569
362	191.1	975	0.180	418	418.2	1128	0.209
363	193.1	191	0.035	419	419.2	219	0.041
364	204.2	83	0.015	420	487.2	539	0.100
365	204.3	25147	4.655	421	503.3	8666	1.604
366	204.4	146	0.027	422	504.3	4451	0.824
367	204.4	101	0.019	423	504.5	115	0.021
368	205.1	391	0.072	424	505.3	3293	0.610
369	205.3	4318	0.799	425	506.3	1733	0.321
370	206.3	290	0.054	426	507.3	348	0.064
371	207.1	2809	0.520	427	564.1	82	0.015
372	208.1	593	0.110				
373	209.1	310	0.057				
374	221.2	2973	0.550				
375	222.2	561	0.104				
376	223.2	293	0.054				
377	249.1	1460	0.270				
378	250.1	167	0.031				
379	251.1	765	0.142				
380	265.1	1159	0.215				
381	266.1	103	0.019				
382	267.1	797	0.148				
383	281.1	20348	3.767				
384	281.3	578	0.107				
385	282.1	6057	1.121				
386	283.1	4064	0.752				
387	284.1	925	0.171				
388	285.1	125	0.023				
389	295.1	98	0.018				
390	311.1	179	0.033				
391	324.1	118	0.022				
392	325.1	3094	0.573				

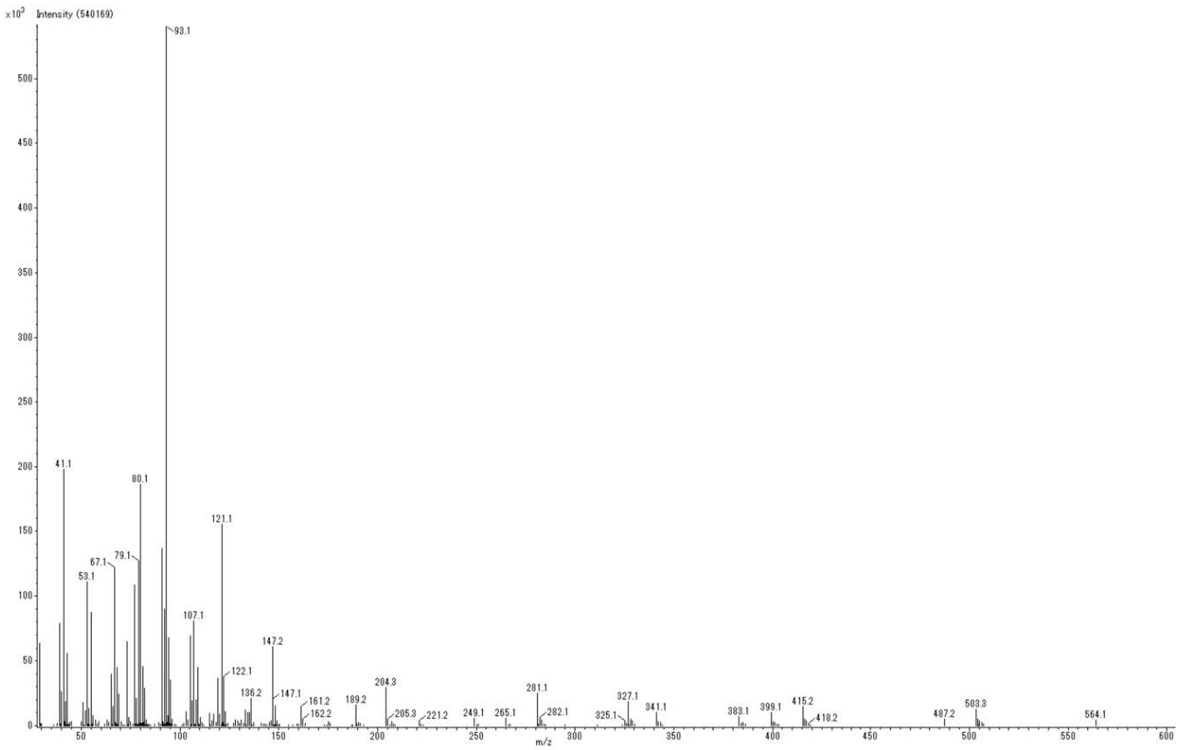

Figure S20a. Mass-spectra of Humelene (Rt= 11.60) detected in *P. nigrum* essential oil by GC- MS

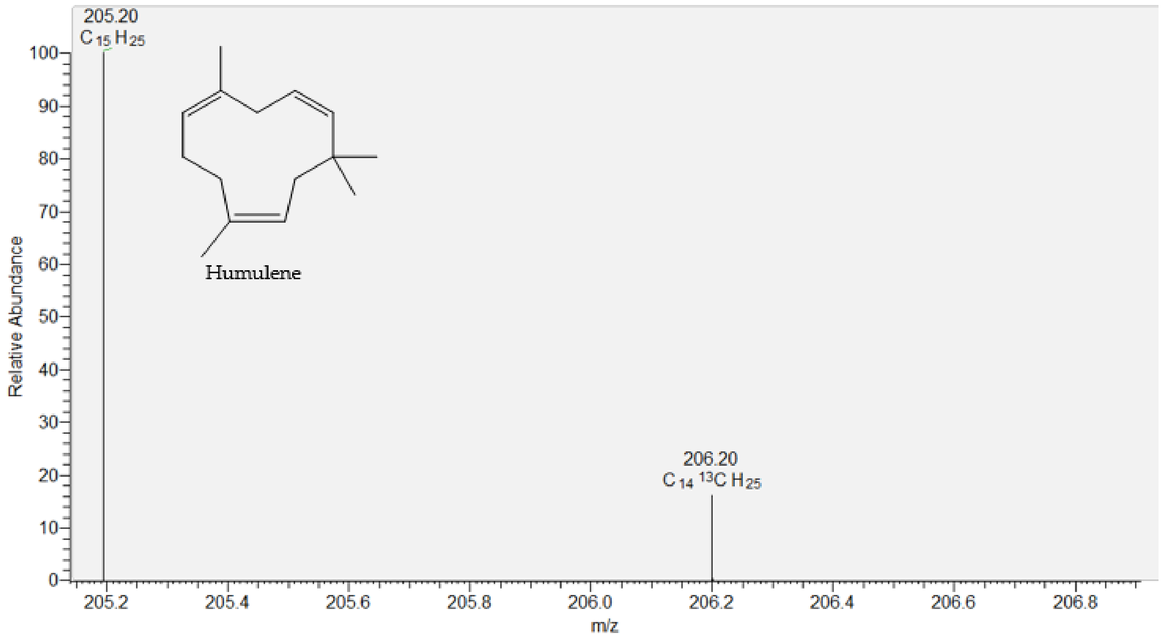

Figure S20b. Spectra of Humulene in *P. nigrum* essential oil identified by positive ion (H^+) adduction method of LC-ESI-MS

Table S19. Fragmentation pattern of β-Selinene (retention time = 11.60) in *P. nigrum* essential oil detected by GC-MS

Peak#	m/z	Intensity	Relative Intensity (%)	Peak#	m/z	Intensity	Relative Intensity (%)
1	29	91	0.067	55	53.4	144	0.106
2	29	37068	27.377	56	54.1	10449	7.717
3	29.1	986	0.728	57	54.1	45	0.033
4	29.1	226	0.167	58	55.1	74217	54.814
5	29.2	42	0.031	59	55.1	660	0.487
6	29.3	264	0.195	60	55.2	240	0.177
7	29.3	32	0.024	61	55.4	378	0.279
8	29.3	154	0.114	62	55.4	178	0.131
9	29.5	164	0.121	63	55.4	260	0.192
10	29.9	57	0.042	64	55.5	123	0.091
11	30	620	0.458	65	55.7	157	0.116
12	31	541	0.400	66	55.7	152	0.112
13	32	85	0.063	67	56.1	5948	4.393
14	32.1	24	0.018	68	57.1	3236	2.390
15	36	79	0.058	69	58.1	23	0.017
16	38	349	0.258	70	59.1	4637	3.425
17	39	49988	36.919	71	60.1	113	0.083
18	39.1	1163	0.859	72	62	142	0.105
19	39.2	162	0.120	73	63	2579	1.905
20	39.3	51	0.038	74	64.1	1406	1.038
21	39.3	278	0.205	75	65.1	27521	20.326
22	39.3	112	0.083	76	65.1	416	0.307
23	40	14636	10.810	77	65.2	333	0.246
24	40.1	292	0.216	78	65.3	69	0.051
25	41.1	135399	100.000	79	65.7	76	0.056
26	41.2	554	0.409	80	66.1	11594	8.563
27	41.3	229	0.169	81	66.1	221	0.163
28	41.3	66	0.049	82	67.1	93262	68.879
29	41.3	932	0.688	83	67.1	215	0.159
30	41.4	642	0.474	84	67.2	619	0.457
31	41.4	51	0.038	85	67.4	51	0.038
32	41.4	134	0.099	86	67.4	107	0.079
33	41.5	129	0.095	87	67.4	289	0.213
34	41.6	958	0.708	88	67.4	324	0.239
35	41.8	71	0.052	89	67.5	324	0.239
36	41.8	254	0.188	90	67.8	47	0.035
37	41.9	443	0.327	91	68.1	36317	26.822
38	42.1	12251	9.048	92	68.2	530	0.391
39	42.1	279	0.206	93	68.4	103	0.076
40	42.4	95	0.070	94	68.5	88	0.065
41	43	3322	2.453	95	69.1	25390	18.752
42	43.1	18406	13.594	96	69.2	631	0.466
43	43.1	581	0.429	97	69.2	28	0.021
44	43.2	240	0.177	98	70.1	2060	1.521
45	43.4	56	0.041	99	71.1	676	0.499
46	44.1	672	0.496	100	71.1	677	0.500
47	50	1294	0.956	101	72.1	125	0.092
48	51	9049	6.683	102	73.1	1029	0.760
49	51.1	75	0.055	103	75.1	198	0.146
50	52	7228	5.338	104	76.1	582	0.430
51	53.1	62731	46.330	105	77.1	64122	47.358
52	53.1	1019	0.753	106	77.1	83	0.061
53	53.2	137	0.101	107	77.2	680	0.502
54	53.4	163	0.120	108	77.2	77	0.057

Table S19. Cont.

Peak#	m/z	Intensity	Relative Intensity (%)	Peak#	m/z	Intensity	Relative Intensity (%)
109	77.3	367	0.271	163	91.1	60	0.044
110	77.4	62	0.046	164	91.2	132	0.097
111	77.4	243	0.179	165	91.3	191	0.141
112	77.5	199	0.147	166	91.4	712	0.526
113	77.8	188	0.139	167	91.4	297	0.219
114	78.1	15113	11.162	168	91.5	308	0.227
115	78.1	356	0.263	169	91.6	555	0.410
116	78.2	156	0.115	170	91.9	260	0.192
117	78.5	86	0.064	171	92.1	20740	15.318
118	79.1	114335	84.443	172	92.2	728	0.538
119	79.2	141	0.104	173	92.2	305	0.225
120	79.2	249	0.184	174	92.3	349	0.258
121	79.4	659	0.487	175	92.6	71	0.052
122	79.4	213	0.157	176	93.1	103530	76.463
123	79.5	911	0.673	177	93.2	126	0.093
124	79.5	688	0.508	178	93.3	393	0.290
125	79.8	268	0.198	179	93.4	100	0.074
126	79.8	298	0.220	180	93.5	721	0.533
127	79.9	301	0.222	181	93.6	327	0.242
128	79.9	68	0.050	182	93.6	236	0.174
129	80.1	32416	23.941	183	93.9	43	0.032
130	80.2	653	0.482	184	93.9	164	0.121
131	80.2	362	0.267	185	94.1	31766	23.461
132	80.5	120	0.089	186	94.2	506	0.374
133	80.5	257	0.190	187	94.2	176	0.130
134	80.5	327	0.242	188	94.3	124	0.092
135	80.6	97	0.072	189	94.6	111	0.082
136	80.8	71	0.052	190	95.1	39972	29.522
137	80.9	86	0.064	191	95.2	606	0.448
138	80.9	77	0.057	192	95.3	317	0.234
139	81.1	75486	55.751	193	95.5	286	0.211
140	81.2	143	0.106	194	96.1	6835	5.048
141	81.3	108	0.080	195	97.1	6751	4.986
142	81.5	189	0.140	196	98.1	598	0.442
143	81.5	307	0.227	197	101.1	74	0.055
144	81.5	295	0.218	198	102.1	1181	0.872
145	81.6	108	0.080	199	103.1	7438	5.493
146	81.8	233	0.172	200	104.1	4328	3.196
147	81.9	294	0.217	201	105.1	93013	68.695
148	81.9	111	0.082	202	105.2	148	0.109
149	82.1	26190	19.343	203	105.3	491	0.363
150	82.2	520	0.384	204	105.5	223	0.165
151	82.3	170	0.126	205	105.5	450	0.332
152	82.5	195	0.144	206	105.6	496	0.366
153	82.6	70	0.052	207	105.6	93	0.069
154	82.9	62	0.046	208	106	359	0.265
155	83	233	0.172	209	106.1	23484	17.344
156	83.1	5085	3.756	210	106.2	401	0.296
157	83.2	62	0.046	211	106.3	157	0.116
158	84.1	338	0.250	212	106.3	193	0.143
159	87.1	453	0.335	213	107.1	87945	64.952
160	89.1	1683	1.243	214	107.2	447	0.330
161	90.1	567	0.419	215	107.3	239	0.177
162	91.1	95004	70.166	216	107.3	209	0.154

Table S19. Cont.

Peak#	m/z	Intensity	Relative Intensity (%)	Peak#	m/z	Intensity	Relative Intensity (%)
163	91.1	60	0.044	217	107.5	230	0.170
164	91.2	132	0.097	218	107.6	213	0.157
165	91.3	191	0.141	219	107.6	327	0.242
166	91.4	712	0.526	220	107.6	440	0.325
167	91.4	297	0.219	221	108	236	0.174
168	91.5	308	0.227	222	108	88	0.065
169	91.6	555	0.410	223	108.1	35	0.026
170	91.9	260	0.192	224	108.1	42817	31.623
171	92.1	20740	15.318	225	108.2	436	0.322
172	92.2	728	0.538	226	108.3	156	0.115
173	92.2	305	0.225	227	108.4	58	0.043
174	92.3	349	0.258	228	109.1	21230	15.680
175	92.6	71	0.052	229	109.2	566	0.418
176	93.1	103530	76.463	230	109.3	216	0.160
177	93.2	126	0.093	231	110.1	3380	2.496
178	93.3	393	0.290	232	111.2	560	0.414
179	93.4	100	0.074	233	112.2	97	0.072
180	93.5	721	0.533	234	115.1	6834	5.047
181	93.6	327	0.242	235	115.2	32	0.024
182	93.6	236	0.174	236	116.1	2878	2.126
183	93.9	43	0.032	237	117.1	10574	7.810
184	93.9	164	0.121	238	118.1	7085	5.233
185	94.1	31766	23.461	239	119.1	45439	33.559
186	94.2	506	0.374	240	119.2	174	0.129
187	94.2	176	0.130	241	119.3	411	0.304
188	94.3	124	0.092	242	119.6	42	0.031
189	94.6	111	0.082	243	120.1	15898	11.742
190	95.1	39972	29.522	244	120.2	132	0.097
191	95.2	606	0.448	245	121.1	58532	43.229
192	95.3	317	0.234	246	121.2	251	0.185
193	95.5	286	0.211	247	121.3	360	0.266
194	96.1	6835	5.048	248	121.4	116	0.086
195	97.1	6751	4.986	249	121.5	65	0.048
196	98.1	598	0.442	250	121.6	28	0.021
197	101.1	74	0.055	251	121.6	264	0.195
198	102.1	1181	0.872	252	121.7	67	0.049
199	103.1	7438	5.493	253	121.7	75	0.055
200	104.1	4328	3.196	254	122.1	52	0.038
201	105.1	93013	68.695	255	122.1	22214	16.406
202	105.2	148	0.109	256	122.2	161	0.119
203	105.3	491	0.363	257	123.2	16925	12.500
204	105.5	223	0.165	258	123.2	381	0.281
205	105.5	450	0.332	259	124.2	2507	1.852
206	105.6	496	0.366	260	127.1	760	0.561
207	105.6	93	0.069	261	128.1	2631	1.943
208	106	359	0.265	262	129.1	2676	1.976
209	106.1	23484	17.344	263	130.1	1541	1.138
210	106.2	401	0.296	264	131.1	5914	4.368
211	106.3	157	0.116	265	132.1	2777	2.051
212	106.3	193	0.143	266	133.1	43681	32.261
213	107.1	87945	64.952	267	133.2	72	0.053
214	107.2	447	0.330	268	133.3	282	0.208
215	107.3	239	0.177	269	133.4	53	0.039
216	107.3	209	0.154	270	133.6	117	0.086

Table S19. Cont.

Peak#	m/z	Intensity	Relative Intensity (%)	Peak#	m/z	Intensity	Relative Intensity (%)
271	133.7	95	0.070	325	176.4	58	0.043
272	134.1	42	0.031	326	177.2	1131	0.835
273	134.2	16381	12.098	327	183.2	352	0.260
274	134.2	158	0.117	328	185	208	0.154
275	134.6	44	0.032	329	187	171	0.126
276	135.2	26183	19.338	330	187.2	694	0.513
277	135.3	382	0.282	331	189.2	33150	24.483
278	135.3	159	0.117	332	189.3	160	0.118
279	136.2	8148	6.018	333	189.4	431	0.318
280	137.1	3471	2.564	334	189.9	81	0.060
281	138.1	94	0.069	335	190.2	5154	3.807
282	141.1	1063	0.785	336	190.3	76	0.056
283	142.1	721	0.533	337	190.3	81	0.060
284	143.1	696	0.514	338	190.5	66	0.049
285	144.1	610	0.451	339	191.2	615	0.454
286	145.1	4433	3.274	340	198.2	239	0.177
287	146.2	2217	1.637	341	202.2	240	0.177
288	147.2	36467	26.933	342	203.2	101	0.075
289	147.2	408	0.301	343	204.3	37946	28.025
290	147.3	385	0.284	344	204.4	190	0.140
291	147.8	127	0.094	345	204.5	48	0.035
292	148.2	20381	15.053	346	204.8	110	0.081
293	148.3	582	0.430	347	205	55	0.041
294	148.7	64	0.047	348	205.3	6593	4.869
295	149.2	9768	7.214	349	205.4	39	0.029
296	150.2	4123	3.045	350	206.3	645	0.476
297	151.2	396	0.292	351	568.3	144	0.106
298	152.1	240	0.177	352	701.2	130	0.096
299	152.2	251	0.185				
300	153.1	212	0.157				
301	155.1	76	0.056				
302	156.2	42	0.031				
303	157.2	197	0.145				
304	159.2	1098	0.811				
305	160.2	1560	1.152				
306	161.2	40188	29.681				
307	161.3	267	0.197				
308	161.3	58	0.043				
309	161.4	131	0.097				
310	161.8	47	0.035				
311	162.1	63	0.047				
312	162.2	16189	11.957				
313	162.3	142	0.105				
314	163.2	4330	3.198				
315	164.2	471	0.348				
316	165.1	78	0.058				
317	166.1	65	0.048				
318	167.2	53	0.039				
319	173.2	157	0.116				
320	174.2	369	0.273				
321	175.2	17992	13.288				
322	175.3	586	0.433				
323	175.8	46	0.034				
324	176.2	8257	6.098				

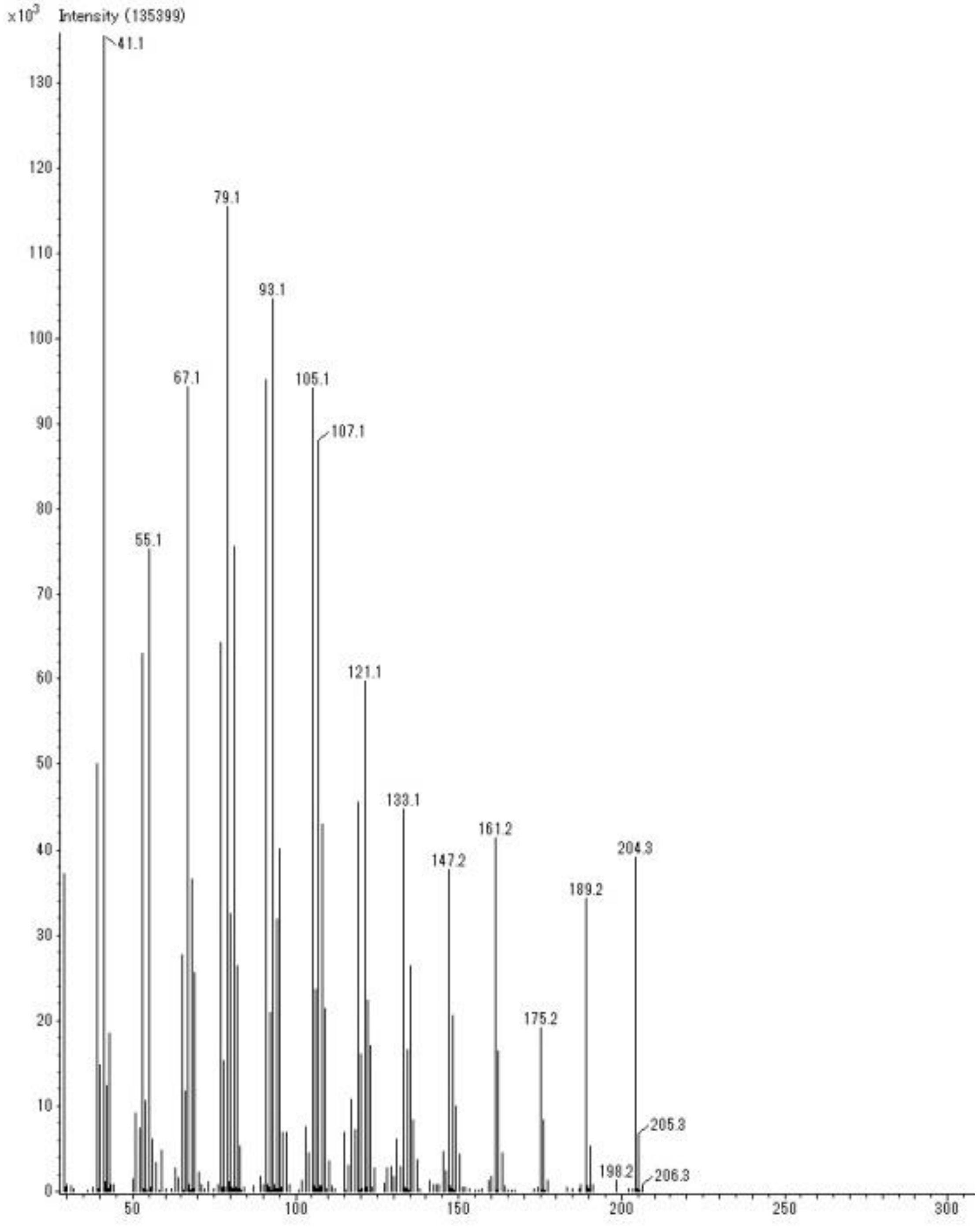

Figure S21a. Mass-spectra of β-Selinene (Rt= 12.03) detected in *P. nigrum* essential oil by GC- MS

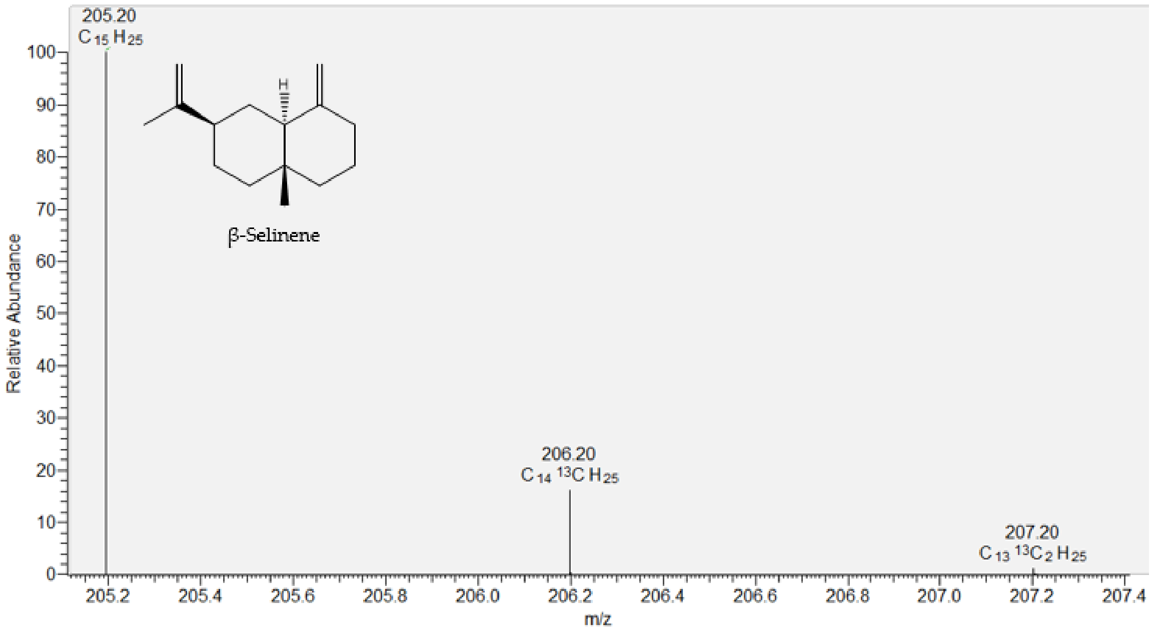

Figure S21b. Spectra of β-Selinene in *P. nigrum* essential oil identified by positive ion (H^+) adduction method of LC-ESI-MS

Table S20. Fragmentation pattern of δ-Cadinene (retention time = 12.33) in *P. nigrum* essential oil detected by GC-MS

Peak#	m/z	Intensity	Relative Intensity (%)	Peak#	m/z	Intensity	Relative Intensity (%)
1	29	121	0.022	57	53.1	106540	19.723
2	29	62923	11.649	58	53.2	1262	0.234
3	29.1	448	0.083	59	53.4	337	0.062
4	29.2	180	0.033	60	53.4	177	0.033
5	29.2	83	0.015	61	53.4	408	0.076
6	29.3	239	0.044	62	53.6	48	0.009
7	29.3	434	0.080	63	53.6	213	0.039
8	29.5	288	0.053	64	53.7	338	0.063
9	29.5	49	0.009	65	53.7	215	0.040
10	30.1	1490	0.276	66	53.7	133	0.025
11	36	89	0.016	67	54	50	0.009
12	38	1232	0.228	68	54	63	0.012
13	39	78676	14.565	69	54.1	12593	2.331
14	39.1	732	0.136	70	54.2	38	0.007
15	39.2	115	0.021	71	54.2	43	0.008
16	39.3	338	0.063	72	55.1	86410	15.997
17	39.3	463	0.086	73	55.1	1065	0.197
18	39.3	286	0.053	74	55.2	193	0.036
19	39.4	108	0.020	75	55.4	173	0.032
20	39.5	58	0.011	76	55.4	51	0.009
21	40	25623	4.744	77	55.4	392	0.073
22	40.1	515	0.095	78	55.4	261	0.048
23	40.1	229	0.042	79	55.5	109	0.020
24	41.1	193186	35.764	80	55.7	372	0.069
25	41.2	785	0.145	81	56	112	0.021
26	41.3	320	0.059	82	56.1	7162	1.326
27	41.3	1139	0.211	83	57.1	3914	0.725
28	41.4	744	0.138	84	58	705	0.131
29	41.6	2588	0.479	85	59.1	2708	0.501
30	41.8	312	0.058	86	62	517	0.096
31	41.8	519	0.096	87	63	4166	0.771
32	41.9	252	0.047	88	64.1	1789	0.331
33	41.9	351	0.065	89	65.1	39028	7.225
34	42.1	17672	3.272	90	65.1	826	0.153
35	42.1	516	0.096	91	65.2	370	0.068
36	42.3	52	0.010	92	65.3	265	0.049
37	42.3	62	0.011	93	65.4	83	0.015
38	42.4	45	0.008	94	65.4	68	0.013
39	42.4	58	0.011	95	66.1	14364	2.659
40	43	280	0.052	96	66.1	296	0.055
41	43.1	55038	10.189	97	66.2	87	0.016
42	43.1	959	0.178	98	67.1	121385	22.472
43	43.2	326	0.060	99	67.2	2221	0.411
44	43.3	156	0.029	100	67.4	73	0.014
45	43.4	107	0.020	101	67.4	202	0.037
46	43.4	204	0.038	102	67.4	475	0.088
47	43.6	98	0.018	103	67.5	443	0.082
48	44	74	0.014	104	67.7	117	0.022
49	44.1	2173	0.402	105	67.8	815	0.151
50	45	2616	0.484	106	67.8	221	0.041
51	50	2487	0.460	107	68.1	44185	8.180
52	51	17160	3.177	108	68.2	659	0.122
53	51.1	313	0.058	109	68.4	50	0.009
54	52	11031	2.042	110	68.4	88	0.016
55	52.1	91	0.017	111	68.5	244	0.045
56	52.1	180	0.033	112	69.1	23548	4.359

Table S20. Cont.

Peak#	m/z	Intensity	Relative Intensity (%)	Peak#	m/z	Intensity	Relative Intensity (%)
113	69.2	561	0.104	169	81.9	138	0.026
114	70.1	2522	0.467	170	82	50	0.009
115	71.1	311	0.058	171	82.1	28255	5.231
116	71.1	223	0.041	172	82.2	80	0.015
117	72.1	210	0.039	173	82.2	663	0.123
118	73.1	64296	11.903	174	82.3	164	0.030
119	73.1	207	0.038	175	82.5	36	0.007
120	73.2	294	0.054	176	82.6	58	0.011
121	73.5	175	0.032	177	83	116	0.021
122	74.1	5542	1.026	178	83.1	4165	0.771
123	74.2	87	0.016	179	83.2	127	0.024
124	75.1	225	0.042	180	84	90	0.017
125	75.1	2855	0.529	181	84.1	360	0.067
126	76.1	906	0.168	182	85.1	282	0.052
127	77.1	107955	19.985	183	87.1	459	0.085
128	77.1	432	0.080	184	89.1	1921	0.356
129	77.2	290	0.054	185	90.1	780	0.144
130	77.3	633	0.117	186	91.1	136481	25.266
131	77.4	67	0.012	187	91.2	673	0.125
132	77.5	507	0.094	188	91.3	466	0.086
133	77.5	298	0.055	189	91.4	2765	0.512
134	77.6	55	0.010	190	91.4	435	0.081
135	77.7	89	0.016	191	91.5	1130	0.209
136	77.8	233	0.043	192	91.6	638	0.118
137	77.9	234	0.043	193	91.6	221	0.041
138	78.1	20255	3.750	194	91.7	40	0.007
139	78.1	425	0.079	195	91.9	506	0.094
140	78.2	214	0.040	196	91.9	161	0.030
141	78.5	276	0.051	197	92.1	89395	16.549
142	79.1	126250	23.372	198	92.2	175	0.032
143	79.2	1747	0.323	199	92.3	559	0.103
144	79.4	604	0.112	200	92.3	131	0.024
145	79.4	310	0.057	201	92.5	326	0.060
146	79.5	716	0.133	202	92.6	562	0.104
147	79.5	1449	0.268	203	92.9	24	0.004
148	79.6	125	0.023	204	92.9	365	0.068
149	79.8	214	0.040	205	93	994	0.184
150	79.8	1007	0.186	206	93	164	0.030
151	79.9	245	0.045	207	93.1	540169	100.000
152	80.1	181976	33.689	208	93.3	272	0.050
153	80.2	361	0.067	209	93.4	260	0.048
154	80.3	160	0.030	210	93.4	98	0.018
155	80.4	53	0.010	211	93.5	905	0.168
156	80.4	400	0.074	212	93.5	1461	0.270
157	80.5	1357	0.251	213	93.6	1684	0.312
158	80.5	1216	0.225	214	93.6	137	0.025
159	80.8	38	0.007	215	93.9	59	0.011
160	80.8	1026	0.190	216	93.9	7173	1.328
161	80.9	280	0.052	217	94	128	0.024
162	81.1	45073	8.344	218	94.1	67245	12.449
163	81.2	135	0.025	219	94.2	204	0.038
164	81.5	169	0.031	220	94.3	212	0.039
165	81.5	1671	0.309	221	94.4	84	0.016
166	81.6	251	0.046	222	94.5	25	0.005
167	81.9	93	0.017	223	94.6	196	0.036
168	81.9	257	0.048	224	94.7	1571	0.291

Table S20. Cont.

Peak#	m/z	Intensity	Relative Intensity (%)	Peak#	m/z	Intensity	Relative Intensity (%)
225	94.7	100	0.019	281	117.1	8642	1.600
226	94.9	101	0.019	282	117.2	127	0.024
227	95	700	0.130	283	118.1	1981	0.367
228	95.1	34784	6.439	284	119.1	35774	6.623
229	95.2	470	0.087	285	119.2	446	0.083
230	95.3	322	0.060	286	119.3	180	0.033
231	95.3	303	0.056	287	119.3	81	0.015
232	95.4	514	0.095	288	120.1	8502	1.574
233	95.5	55	0.010	289	121	61	0.011
234	96.1	4225	0.782	290	121.1	151066	27.966
235	96.2	174	0.032	291	121.2	431	0.080
236	97.1	909	0.168	292	121.3	179	0.033
237	98	83	0.015	293	121.5	339	0.063
238	101.1	100	0.019	294	121.7	1759	0.326
239	102.1	1410	0.261	295	122.1	102	0.019
240	103.1	10306	1.908	296	122.1	37277	6.901
241	103.2	212	0.039	297	122.3	92	0.017
242	104.1	3967	0.734	298	122.4	44	0.008
243	105.1	68443	12.671	299	122.5	80	0.015
244	105.2	276	0.051	300	122.5	31	0.006
245	105.3	104	0.019	301	122.5	87	0.016
246	105.5	46	0.009	302	122.6	202	0.037
247	105.5	35	0.006	303	123.2	10061	1.863
248	105.6	65	0.012	304	123.3	59	0.011
249	105.6	180	0.033	305	124.2	1202	0.223
250	106	62	0.011	306	127.1	1259	0.233
251	106.1	18340	3.395	307	128.1	3890	0.720
252	106.2	392	0.073	308	129.1	3275	0.606
253	106.2	70	0.013	309	130.1	1412	0.261
254	107.1	76525	14.167	310	131.1	968	0.179
255	107.2	197	0.036	311	131.1	4079	0.755
256	107.3	412	0.076	312	132.1	1033	0.191
257	107.5	73	0.014	313	133.1	1236	0.229
258	107.6	211	0.039	314	133.1	11503	2.130
259	107.6	268	0.050	315	133.2	210	0.039
260	107.7	128	0.024	316	133.3	103	0.019
261	108	24	0.004	317	134.2	9549	1.768
262	108.1	49	0.009	318	134.2	238	0.044
263	108.1	19554	3.620	319	135.2	9500	1.759
264	108.2	82	0.015	320	136.2	17019	3.151
265	108.3	40	0.007	321	136.3	155	0.029
266	109.1	44133	8.170	322	136.3	40	0.007
267	109.3	605	0.112	323	137.2	2171	0.402
268	109.6	51	0.009	324	141.1	1358	0.251
269	109.6	95	0.018	325	142.1	882	0.163
270	109.6	108	0.020	326	143.1	883	0.163
271	109.7	106	0.020	327	144.1	277	0.051
272	110	84	0.016	328	145.1	2014	0.373
273	110.1	62	0.011	329	146.2	3227	0.597
274	110.1	5721	1.059	330	147.1	19993	3.701
275	111.2	2024	0.375	331	147.2	56455	10.451
276	112.2	82	0.015	332	147.3	304	0.056
277	115.1	8787	1.627	333	147.3	310	0.057
278	115.2	307	0.057	334	147.7	188	0.035
279	116.1	3182	0.589	335	147.8	77	0.014
280	117.1	972	0.180	336	148.1	3444	0.638

Table S20. Cont.

Peak#	m/z	Intensity	Relative Intensity (%)	Peak#	m/z	Intensity	Relative
337	148.2	14809	2.742	393	326.1	1148	0.213
338	148.3	220	0.041	394	327.1	13948	2.582
339	148.7	101	0.019	395	327.2	101	0.019
340	149.1	1946	0.360	396	328.1	4514	0.836
341	149.2	3375	0.625	397	329.1	3140	0.581
342	150.1	90	0.017	398	330.1	774	0.143
343	150.2	463	0.086	399	341.1	6030	1.116
344	155.2	87	0.016	400	341.3	103	0.019
345	157.2	321	0.059	401	342.1	2303	0.426
346	159.2	677	0.125	402	343.1	1719	0.318
347	160.2	331	0.061	403	344.1	299	0.055
348	161.2	13972	2.587	404	383.1	2827	0.523
349	161.3	298	0.055	405	384.1	1120	0.207
350	162.2	4274	0.791	406	385.1	1709	0.316
351	163.2	1107	0.205	407	386.1	884	0.164
352	173.2	105	0.019	408	399.1	5697	1.055
353	174.2	82	0.015	409	399.3	97	0.018
354	175.2	2576	0.477	410	400.1	2330	0.431
355	176.2	1230	0.228	411	401.1	2015	0.373
356	187	254	0.047	412	402.1	769	0.142
357	187.2	321	0.059	413	403.1	85	0.016
358	189.1	207	0.038	414	415.2	10355	1.917
359	189.2	11609	2.149	415	415.3	114	0.021
360	189.3	421	0.078	416	416.2	4368	0.809
361	190.2	1966	0.364	417	417.2	3071	0.569
362	191.1	975	0.180	418	418.2	1128	0.209
363	193.1	191	0.035	419	419.2	219	0.041
364	204.2	83	0.015	420	487.2	539	0.100
365	204.3	25147	4.655	421	503.3	8666	1.604
366	204.4	146	0.027	422	504.3	4451	0.824
367	204.4	101	0.019	423	504.5	115	0.021
368	205.1	391	0.072	424	505.3	3293	0.610
369	205.3	4318	0.799	425	506.3	1733	0.321
370	206.3	290	0.054	426	507.3	348	0.064
371	207.1	2809	0.520	427	564.1	82	0.015
372	208.1	593	0.110				
373	209.1	310	0.057				
374	221.2	2973	0.550				
375	222.2	561	0.104				
376	223.2	293	0.054				
377	249.1	1460	0.270				
378	250.1	167	0.031				
379	251.1	765	0.142				
380	265.1	1159	0.215				
381	266.1	103	0.019				
382	267.1	797	0.148				
383	281.1	20348	3.767				
384	281.3	578	0.107				
385	282.1	6057	1.121				
386	283.1	4064	0.752				
387	284.1	925	0.171				
388	285.1	125	0.023				
389	295.1	98	0.018				
390	311.1	179	0.033				
391	324.1	118	0.022				
392	325.1	3094	0.573				

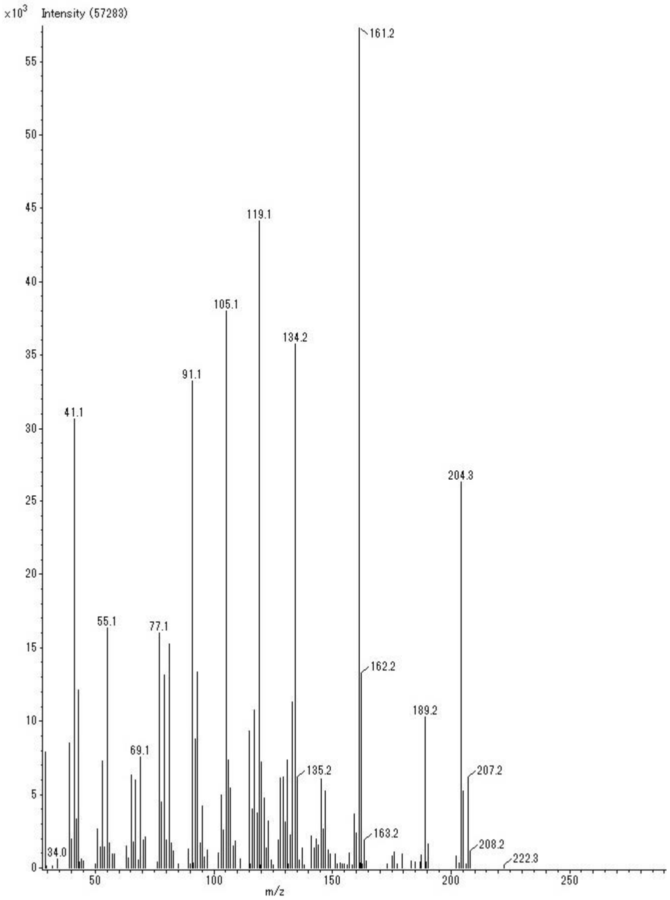

Figure S22a. Mass-spectra of δ-Cadinene (Rt= 12.33) detected in *P. nigrum* essential oil by GC- MS

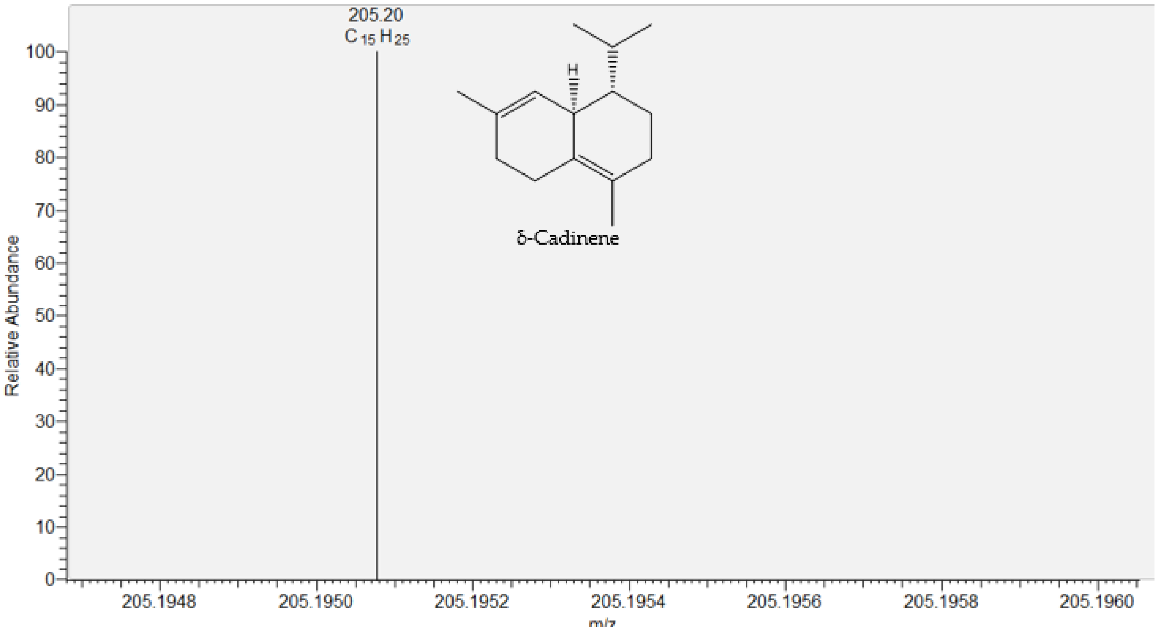

Figure S22b. Spectra of δ-Cadinene in *P. nigrum* essential oil identified by positive ion (H^+) adduction method of LC-ESI-MS

Table S21. Fragmentation pattern of Caryophylene oxide (retention time = 13.16) in *P. nigrum* essential oil detected by GC-MS

Peak#	m/z	Intensity	Relative Intensity (%)	Peak#	m/z	Intensity	Relative Intensity (%)
1	29	1790	2.091	55	55.2	385	0.450
2	29	16660	19.461	56	55.2	139	0.162
3	29.1	539	0.630	57	56	516	0.603
4	30.1	457	0.534	58	56.1	10463	12.222
5	31	963	1.125	59	56.1	115	0.134
6	38	111	0.130	60	57.1	2971	3.471
7	38	339	0.396	61	57.1	2700	3.154
8	39	28491	33.282	62	58.1	2713	3.169
9	39.1	638	0.745	63	59.1	1413	1.651
10	39.1	251	0.293	64	62.3	96	0.112
11	40	29	0.034	65	63	1293	1.510
12	40	6877	8.033	66	64.1	499	0.583
13	40.1	130	0.152	67	65.1	12964	15.144
14	40.1	85	0.099	68	65.1	303	0.354
15	41.1	85606	100.000	69	65.2	158	0.185
16	41.1	547	0.639	70	66.1	5176	6.046
17	41.3	357	0.417	71	67.1	51	0.060
18	41.3	160	0.187	72	67.1	27512	32.138
19	41.4	275	0.321	73	67.1	300	0.350
20	41.4	102	0.119	74	67.2	168	0.196
21	41.6	67	0.078	75	67.5	86	0.100
22	41.6	166	0.194	76	68	386	0.451
23	41.6	138	0.161	77	68.1	7726	9.025
24	41.6	136	0.159	78	69.1	5291	6.181
25	41.9	83	0.097	79	69.1	30738	35.906
26	42	747	0.873	80	69.2	961	1.123
27	42.1	5091	5.947	81	69.2	386	0.451
28	42.1	86	0.100	82	69.5	73	0.085
29	42.1	72	0.084	83	70.1	776	0.906
30	43	65401	76.398	84	70.1	2938	3.432
31	43.1	16347	19.096	85	71.1	9772	11.415
32	43.1	399	0.466	86	71.1	469	0.548
33	43.3	39	0.046	87	72.1	1060	1.238
34	43.3	13	0.015	88	76.1	104	0.121
35	43.3	243	0.284	89	77.1	29509	34.471
36	43.4	308	0.360	90	77.1	186	0.217
37	43.4	65	0.076	91	77.2	88	0.103
38	43.6	398	0.465	92	77.3	44	0.051
39	43.6	95	0.111	93	77.4	53	0.062
40	44	328	0.383	94	78.1	9184	10.728
41	44	3907	4.564	95	78.1	83	0.097
42	44.1	714	0.834	96	79.1	55873	65.268
43	44.1	155	0.181	97	79.2	267	0.312
44	45	2083	2.433	98	79.2	453	0.529
45	50	741	0.866	99	79.2	132	0.154
46	51	4917	5.744	100	79.4	75	0.088
47	52	3856	4.504	101	79.5	271	0.317
48	53.1	22372	26.134	102	79.5	305	0.356
49	53.1	343	0.401	103	80.1	10197	11.912
50	53.2	156	0.182	104	80.1	76	0.089
51	54.1	4645	5.426	105	80.2	364	0.425
52	55	3636	4.247	106	80.5	40	0.047
53	55.1	30569	35.709	107	81.1	675	0.788
54	55.1	648	0.757	108	81.1	20454	23.893

Table S21. Cont.

Peak#	m/z	Intensity	Relative Intensity (%)	Peak#	m/z	Intensity	Relative Intensity (%)
109	81.2	566	0.661	163	110.1	2853	3.333
110	81.2	117	0.137	164	110.1	8323	9.722
111	82.1	1313	1.534	165	111.1	4516	5.275
112	82.1	9699	11.330	166	112.1	415	0.485
113	82.1	194	0.227	167	115.1	2198	2.568
114	82.2	128	0.150	168	116.1	1066	1.245
115	83	182	0.213	169	117.1	3876	4.528
116	83.1	2250	2.628	170	118.1	1975	2.307
117	83.1	8287	9.680	171	119.1	14013	16.369
118	83.2	92	0.107	172	119.2	182	0.213
119	84.1	1886	2.203	173	120.1	8580	10.023
120	84.1	689	0.805	174	120.2	66	0.077
121	85.1	3800	4.439	175	121.1	15045	17.575
122	85.1	279	0.326	176	121.2	550	0.642
123	89.1	195	0.228	177	122.1	787	0.919
124	90.1	91	0.106	178	122.1	4151	4.849
125	91.1	43776	51.137	179	123.1	9045	10.566
126	91.2	1423	1.662	180	123.2	88	0.103
127	91.2	825	0.964	181	124.1	2328	2.719
128	91.3	80	0.093	182	125.1	1398	1.633
129	91.4	232	0.271	183	126.1	451	0.527
130	92.1	8269	9.659	184	127.2	844	0.986
131	92.2	56	0.065	185	128.1	845	0.987
132	92.3	70	0.082	186	129.1	1248	1.458
133	93.1	41030	47.929	187	130.1	489	0.571
134	93.2	581	0.679	188	131.1	5230	6.109
135	93.2	205	0.239	189	132.1	1473	1.721
136	94.1	73	0.085	190	133.1	6254	7.306
137	94.1	10809	12.626	191	134.1	97	0.113
138	95.1	3370	3.937	192	134.2	5242	6.123
139	95.1	20913	24.429	193	135.1	2438	2.848
140	95.2	445	0.520	194	135.2	4298	5.021
141	96.1	9871	11.531	195	136.1	2383	2.784
142	96.1	4863	5.681	196	136.2	2302	2.689
143	97.1	3368	3.934	197	137.1	3148	3.677
144	97.1	756	0.883	198	138.1	4458	5.208
145	98.1	676	0.790	199	139.2	1242	1.451
146	99.1	738	0.862	200	140.2	326	0.381
147	102.1	128	0.150	201	141.1	91	0.106
148	103.1	3050	3.563	202	143.1	235	0.275
149	104.1	1853	2.165	203	144.1	544	0.635
150	105.1	19825	23.158	204	145.1	3219	3.760
151	105.2	229	0.268	205	146.2	1828	2.135
152	105.3	59	0.069	206	147.2	7597	8.874
153	106.1	17428	20.358	207	148.2	2187	2.555
154	106.2	332	0.388	208	149.2	6289	7.346
155	106.3	109	0.127	209	150.2	966	1.128
156	107.1	21325	24.911	210	150.2	861	1.006
157	107.2	498	0.582	211	151.2	1812	2.117
158	108.1	1259	1.471	212	152.2	636	0.743
159	108.1	6961	8.131	213	159.2	3754	4.385
160	109.1	6550	7.651	214	160.2	1634	1.909
161	109.1	11782	13.763	215	161.2	4142	4.838
162	109.2	254	0.297	216	162.2	5047	5.896

Table S21. Cont.

Peak#	m/z	Intensity	Relative Intensity (%)
217	163.2	2934	3.427
218	164.2	2168	2.533
219	165.2	778	0.909
220	173	82	0.096
221	173.2	555	0.648
222	174.2	673	0.786
223	175.1	83	0.097
224	175.2	548	0.640
225	176.2	1263	1.475
226	177.2	3764	4.397
227	178.2	691	0.807
228	187.2	2459	2.872
229	188.2	117	0.137
230	189.2	272	0.318
231	191.2	803	0.938
232	192.2	658	0.769
233	202.2	793	0.926
234	205.2	2040	2.383
235	206.2	107	0.125
236	220.3	1486	1.736
237	221.3	189	0.221

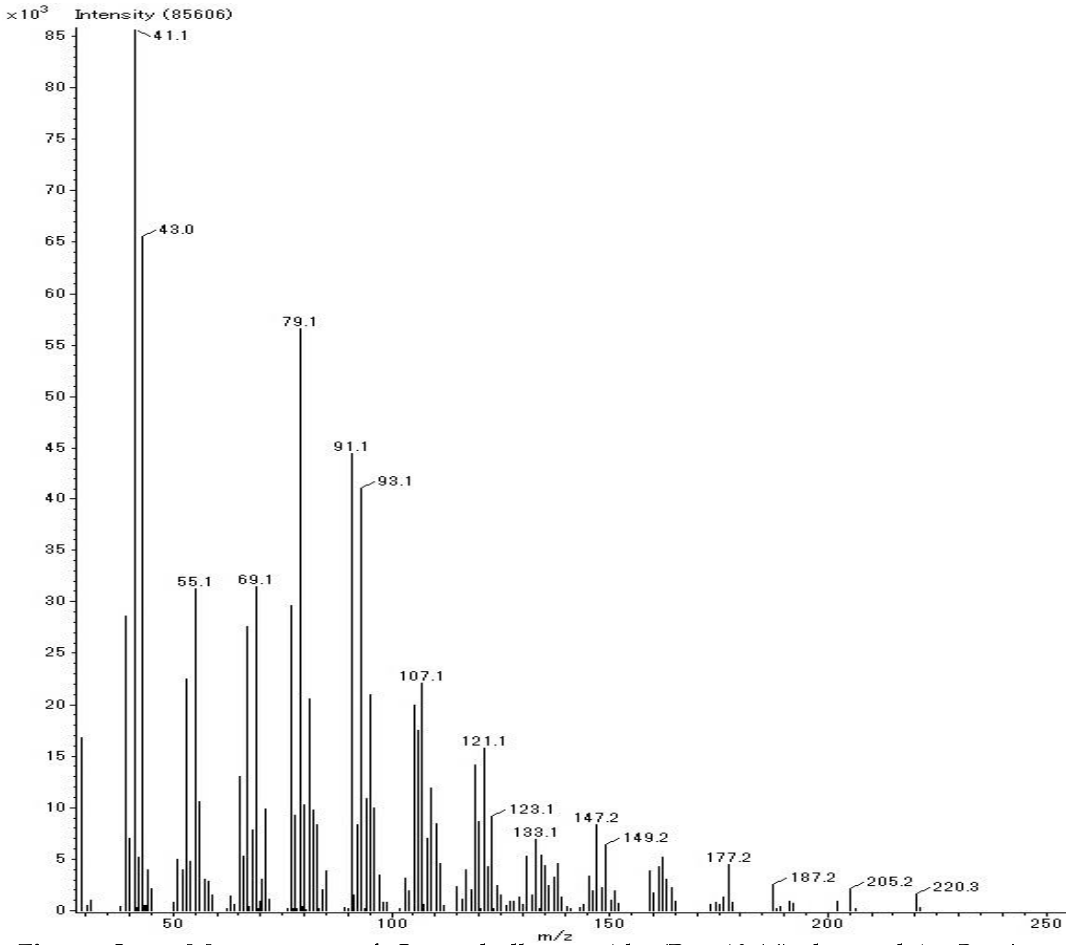

Figure S23a. Mass-spectra of Caryophyllene oxide (Rt= 13.16) detected in *P. nigrum* essential oil by GC- MS

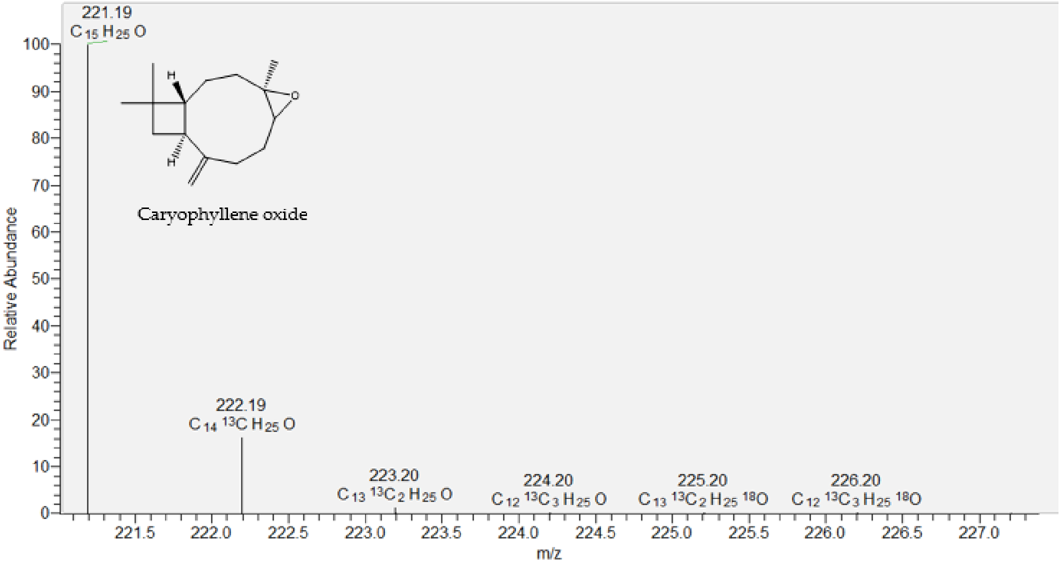

Figure S23b. Spectra of Caryophyllene oxide in *P. nigrum* essential oil identified by positive ion (H^+) adduction method of LC-ESI-MS

Table S22. Fragmentation pattern of Cubenol (retention time = 13.65) in *P. nigrum* essential oil detected by GC-MS

Peak#	m/z	Intensity	Relative Intensity (%)	Peak#	m/z	Intensity	Relative Intensity (%)
1	29	269	1.364	54	89.1	871	4.417
2	29	4038	20.476	55	91.1	15964	80.949
3	30	379	1.922	56	91.2	572	2.900
4	31	586	2.971	57	92.1	3503	17.763
5	32	809	4.102	58	93.1	9937	50.388
6	35	367	1.861	59	94.1	2063	10.461
7	38	469	2.378	60	95.1	2363	11.982
8	39	4509	22.864	61	95.1	3378	17.129
9	40	1773	8.990	62	96.1	569	2.885
10	41.1	16587	84.108	63	96.1	880	4.462
11	41.1	158	0.801	64	97.1	1175	5.958
12	42.1	1614	8.184	65	102.1	412	2.089
13	43	19721	100.000	66	103.1	2593	13.148
14	43.1	8836	44.805	67	104.1	1862	9.442
15	43.1	662	3.357	68	104.2	242	1.227
16	44	46	0.233	69	105.1	11528	58.455
17	44	733	3.717	70	105.2	357	1.810
18	44.1	463	2.348	71	106.1	3434	17.413
19	45	1202	6.095	72	107.1	651	3.301
20	51	1563	7.926	73	107.1	6474	32.828
21	52	836	4.239	74	108.1	595	3.017
22	53.1	5332	27.037	75	108.1	1850	9.381
23	54.1	908	4.604	76	109.1	2532	12.839
24	55	1572	7.971	77	109.1	1759	8.919
25	55.1	7676	38.923	78	110.1	1453	7.368
26	56.1	1107	5.613	79	111.1	1214	6.156
27	57.1	1712	8.681	80	115.1	2635	13.361
28	57.1	1246	6.318	81	116.1	1276	6.470
29	58.1	3443	17.459	82	117.1	5218	26.459
30	59.1	212	1.075	83	117.1	341	1.729
31	63	489	2.480	84	118.1	1976	10.020
32	64.1	291	1.476	85	119.1	18961	96.146
33	65.1	3355	17.012	86	120.1	3261	16.536
34	66.1	1396	7.079	87	121.1	4063	20.602
35	67.1	5353	27.144	88	122.1	361	1.831
36	68.1	1120	5.679	89	122.1	2492	12.636
37	69.1	1208	6.125	90	123.1	1821	9.234
38	69.1	4637	23.513	91	123.2	952	4.827
39	70.1	574	2.911	92	124.1	402	2.038
40	71.1	3619	18.351	93	125.1	770	3.904
41	71.1	413	2.094	94	126.1	525	2.662
42	72.1	892	4.523	95	127.1	514	2.606
43	77.1	7580	38.436	96	128.1	1444	7.322
44	78.1	2356	11.947	97	129.1	1698	8.610
45	79.1	9617	48.765	98	130.1	673	3.413
46	80.1	1914	9.705	99	131.1	3985	20.207
47	81.1	5580	28.295	100	132.1	883	4.477
48	82.1	653	3.311	101	133.1	2532	12.839
49	82.1	3160	16.024	102	134.2	2891	14.660
50	83.1	952	4.827	103	135.2	2928	14.847
51	83.1	1328	6.734	104	136.2	899	4.559
52	84.1	686	3.479	105	137.1	488	2.475
53	85.1	2089	10.593	106	141.1	649	3.291

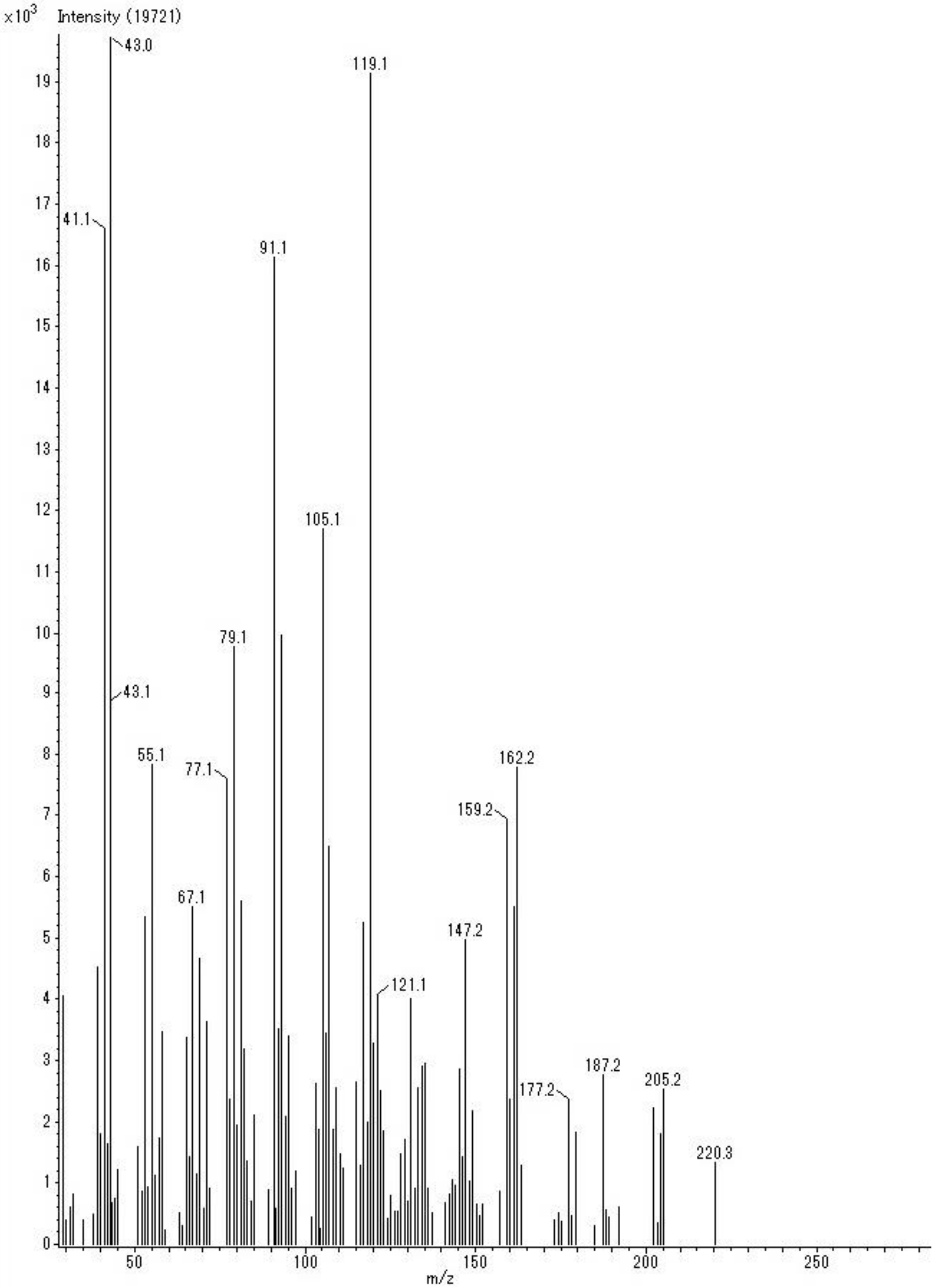

Figure S24a. Mass-spectra of Cubenol (Rt= 13.65) detected in *P. nigrum* essential oil by GC- MS

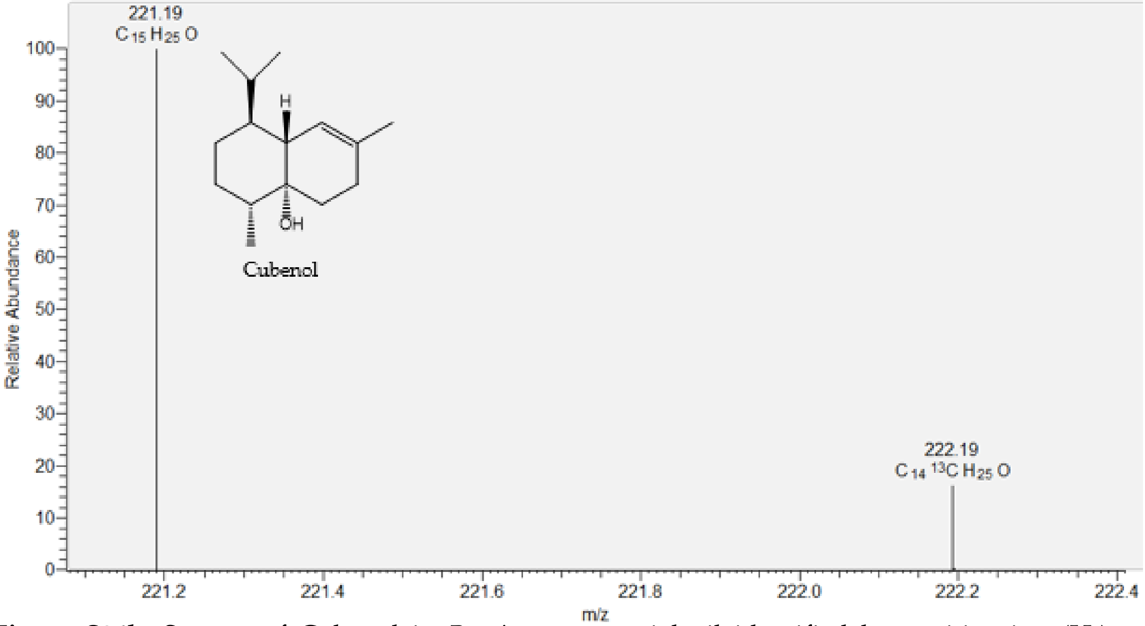

Figure S24b. Spectra of Cubenol in *P. nigrum* essential oil identified by positive ion (H^+) adduction method of LC-ESI-MS
